# Controlling Electronic Events Through Rational Structural Design in Subphthalocyanine–Corrole Dyads: Synthesis, Characterization, and Photophysical Properties

**DOI:** 10.1002/chem.202201552

**Published:** 2022-09-01

**Authors:** Víctor Mariñas, Benedikt Platzer, Jorge Labella, Fabrizio Caroleo, Sara Nardis, Roberto Paolesse, Dirk M. Guldi, Tomás Torres

**Affiliations:** ^1^ Department of Chemical Science and Technologies University of Rome Tor Vergata Via della Ricerca Scientifica 00133 Rome Italy; ^2^ Department of Organic Chemistry Universidad Autónoma de Madrid Campus de Cantoblanco C/ Francisco Tomás y Valiente 7 28049 Madrid Spain; ^3^ IMDEA – Nanociencia C/ Faraday 9, Campus de Cantoblanco 28049 Madrid Spain; ^4^ Institute for Advanced Research in Chemical Sciences (IAdChem) Universidad Autónoma de Madrid Madrid Spain; ^5^ Department of Chemistry and Pharmacy Interdisciplinary Center for Molecular Materials (ICMM) Friedrich-Alexander-Universität Erlangen-Nürnberg Egerlandstr. 3 91058 Erlangen Germany

**Keywords:** corroles, electron transfer, organic photovoltaics, porphyrinoids, subphthalocyanines

## Abstract

Porphyrinoids are considered perfect candidates for their incorporation into electron donor–acceptor (D–A) arrays due to their remarkable optoelectronic properties and low reorganization energies. For the first time, a series of subphthalocyanine (SubPc) and corrole (Cor) were covalently connected through a short‐range linkage. SubPc axial substitution strategies were employed, which allowed the synthesis of the target molecules in decent yields. In this context, a qualitative synthetic approach was performed to reverse the expected direction of the different electronic events. Consequently, in‐depth absorption, fluorescence, and electrochemical assays enabled the study of electronic and photophysical properties. Charge separation was observed in cases of electron‐donating Cors, whereas a quantitative energy transfer from the Cor to the SubPc was detected in the case of electron accepting Cors.

## Graphical abstract

**A series of covalently linked subphthalocyanine–corrole** (SubPc‐Cor) dyads have been prepared. In four of the reported examples, Cor⋅^+^‐SubPc⋅^−^ charge‐separated states have been observed, whereas in the case of the electron‐rich SubPc and the electron‐deficient Cor dyad, only energy transfer has been detected.

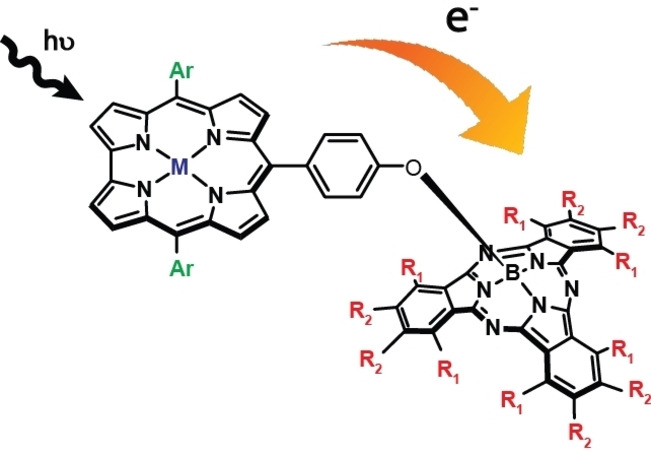

## Introduction

Over the last years, photochemical conversion of solar energy has become a prominent topic, fostered by the progressive decrease of the fossil fuels reserves.^[1]^ In this regard, electron donor‐acceptor (D–A) systems have experienced a growing interest for their ability to generate long‐lived charge‐separation states (CSS) upon capture of photons which, in turn, may be transformed into useful forms of energy.^[2]^ According to Marcus’ theory of charge transfer,^[3]^ the rates of charge separation (CS) and charge recombination (CR) in photoinduced electron transfer (PeT) processes can be quantitatively measured by means of several physical and chemical parameters, as described in equation 1:

(1) $${\;k}_{eT}=v_{N}\kappa \exp([\left(-\Delta G^{o}+\;{\lambda )}^{2}/4\lambda \right]/RT)$$

Marcus’ theory model provides a counterintuitive statement: as the driving force (−ΔG^o^) becomes more negative, the rate constant (*K_eT_)* increases. When the magnitude of ΔG^o^ equals the reorganization energy (λ), the reaction rate reaches a maximum, displaying a parabolic response and entering into the area referred as the Marcus *inverted* region, in which higher values of −ΔG^o^ give rise to lower constant rates. Marcus defined λ as the energy required for the electron donor and the electron acceptor to structurally reorganize with their solvation spheres upon electron transfer (eT). Low values of λ translate into high CS rates, whereas higher values of −ΔG^o^ slow down the CR process, which are ultimately desired.^[4]^ Molecules with π‐extended electronic distributions and rigid structures present low reorganization energy values, because of the small structural differences between their neutral and charged species. As a consequence, porphyrinoids play an important role in nature, being involved as porphyrins (Pors), chlorins or bacteriochlorins in diverse photosynthetic eT processes (e.g., photosynthesis).^[5]^

In artificial photosynthetic systems, broadening the overall absorption spectrum with high extinction coefficient values is required for efficiently use the photon energy.^[6]^ In an attempt to mimic nature, porphyrinoids have been largely employed as photosensitizers in D–A systems, such as Pors,^[7]^ corroles (Cors),^[8]^ phthalocyanines (Pcs),^[9]^ subphthalocyanines (SubPcs),^[10]^ in the form of dyads or myriads^[11]^ and in combination with a wide number of aromatic carbon nanostructures and other electroactive units, either covalently or through supramolecular interactions.^[12]^ As electron donors, Pors have been preferred over the rest of porphyrinoids due to their appropriate photophysical properties and synthetic accessibility.^[13]^ In this regard, Cors,^[14]^ contracted analogs of Pors, are selected as better suitable donors. Following identical substitution patterns, they display enhanced optoelectronic properties, such as easiness of oxidation, extended absorption ranges and high molar absorption coefficients.^[15]^ At the same time, fullerenes have prevailed as the main acceptor counterparts for D–A systems due to their excellent photophysical features, like low reorganization energies and low reduction potentials.^[16]^ However, they present several drawbacks, such as low intensity absorption spectra in the visible region and lack of synthetic flexibility, reducing the possibilities of optical and structural tuning of the molecule.

The field of artificial photosynthetic systems has registered a rising interest in the design of novel non‐fullerene acceptor (NFA) molecules. SubPcs^[17]^ are non‐planar aromatic macrocycles composed by three 1,3‐diiminoisoindole units N‐fused around a central boron atom. The particular bowl‐shaped geometry of SubPcs comes mainly from the tetrahedral coordination imposed by the boron, which presents a characteristic axial position, and from the intrinsic contraction of the ligand that, in comparison with Pcs, would not be able to adopt a planar disposition. They present a 14 π‐electron system largely around its inner cavity, displaying high excitation and low reorganization energy values, along with high molar absorption coefficients and redox potentials that can be finely tailored by concrete substitution patterns. In combination with Cors, they are able to cover a wide area of the solar radiation spectra, with absorption bands located between 300–460 and 500–650 nm, coming from their S_0_–S_2_ and S_0_–S_1_ electronic transitions, respectively. Moreover, the modulation of their electronic behavior via synthetic derivatizations allows for a better control of their corresponding energy levels.

**Figure 1:**
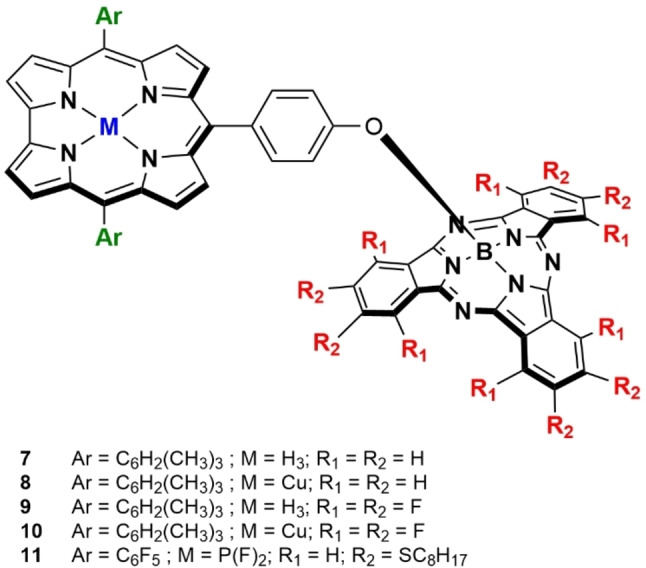
Molecular structures of SubPc‐Cor electron D–A conjugates.

In this work, we report the synthesis of SubPc‐Cor conjugates by axial substitution reaction of the SubPc by Cor derivatives through a phenoxy‐ bridge located at their *meso*‐ position, which enabled in‐depth study of its electronic and photophysical properties (Figure 1). To the best of our knowledge, this is the first report of Cors and SubPcs covalently connected through a short‐range linker. To study the electronic versatility of this family of dyads, we considered two different approaches. First, the insertion of copper into free‐base Cor conjugates was tackled in order to probe the introduction of additional reduction potentials. Although copper corroles (CuCor) are better known for their behavior as non‐innocent ligands,^[18]^ their role as electron acceptor molecules in D–A schemes was recently highlighted by D'Souza and co‐workers.^[19]^ Second, the fewer reports in the literature of Cors acting as electron acceptor ligands in D–A systems led us to further explore their possibilities by swapping their electronic features, including a highly electro‐deficient phosphorus corrole (PCor) as the acceptor and an electron‐rich SubPc as the donor.

By means of rational electrochemical and photophysical experiments, we were able to determine CSS in all the D–A conjugates comprising electron donor Cors. For the conjugate including PCor, however, only energy transfer (ET) from the PCor to the SubPc was demonstrated, enhancing his role as an antenna.

## Results and Discussion

### Synthesis

In the present study, the anchoring point of the SubPc is always related to its axial position. One of the most studied reactions for SubPcs axial substitution employs phenoxy‐ fragments and requires high excess of the nucleophile, high temperatures and long reaction times, thus limiting the preparation of different phenoxy‐derivatives. Recently, a mild activation methodology based on the use of triflate anions was reported by our group, significantly reducing the number of nucleophile equivalents, temperature and reaction times.^[20]^ On the other hand, multiple synthetic approaches can be chosen for the Cor subunit, involving the *beta*‐, *meso*‐ or, if properly complexed, axial positions. Among the different synthetic strategies, array expansion from the *meso*–position for D–A conjugates is preferred for several reasons: (1) *meso*‐substituted Cors can be obtained from commercially available reagents, in few reaction steps and satisfying yields; (2) it allows for the straightforward introduction of a variety of anchoring points and functionalities into Cor scaffold; (3) theoretical studies have long indicated that Cors should follow Gouterman's four‐orbital model,^[21]^ being their molecular orbitals qualitatively similar to those of Pors. For Pors, it has been determined that the position of the HOMO (a_2u_ in Pors, b_1_ in Cors) involved in the energy and electron transfer processes is mainly located at the C_meso_ position, thus favoring the connection among the D–A conjugates.^[12]^

**Scheme 1:**
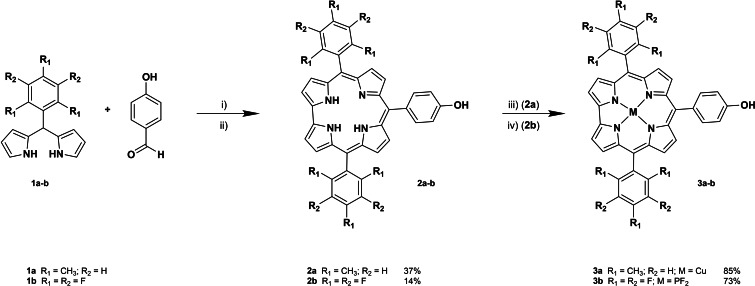
Synthetic route for the obtention of Cors **2 a**–**b** and **3 a**–**b**. i) MeOH+HCl 0.55 M, dark, RT, 2 h; ii) CHCl_3_, *p*‐chloranil (1.25 equiv), dark, RT, 16 h; iii) Cu(OAc)_2_, CHCl_3_, 40 °C, 15 min.; iv) a) Pyridne, PCl_3_, reflux, 30 min. b) CH_2_Cl_2_, HF 50 %, dark, RT, 16 h.

We synthesized a series of *trans*‐A_2_B Cors bearing a phenoxy unit at their 10‐*meso*‐ position. For the synthesis of *trans*‐A_2_B Cors, the use of dipyrromethanes (DPMs) is generally required. Mesityl‐dipyrromethane (Mes‐DPM) was synthesized following a mild methodology developed by Dolenský and co‐workers.^[22]^ For the synthesis of the *trans*‐A_2_B Cors, a specific approach reported in the literature, particularly efficient for bulky DPMs, was followed (Scheme 1).^[23]^ As reflected by the author, the ratio within the reaction components is imperative for the preparation of the desired Cors, due to the many consecutive condensation side reactions happening concurrently. With this in mind, different conditions were investigated to optimize the synthetic procedure for the obtention of the free‐base Cor **2 a** (Table 1).

**Table 1:** Reaction conditions and isolated yields [%] for the obtention of Cor **2 a**.

Entry	DPM^[c]^	Aldehyde^[c]^	MeOH^[d]^	HCl^[d]^	CHCl_3_^[d]^	Yield
1^[a]^	0.5	0.25	50	26.3	125	15
2^[a]^	1	0.5	100	52.5	250	15
3^[a]^	1	0.5	50	52.5	250	37
4^[b]^	1	0.5	50	52.5	250	22
5^[b]^	1	0.5	75	52.5	250	13
6^[b]^	1	0.5	100	52.5	250	–
7^[b]^	2	1	100	105	500	13

[a] *p*‐Chloranil as oxidant. [b] DDQ as oxidant. [c] in mmol. [d] in mL.

As depicted in Table 1, the relationship between the different reagents, as well as the HCl/CHCl_3_ ratio, was kept constant. Scaling the reaction did not influence in the yield (entries 1–2). However, half‐reduction of the MeOH volume dramatically increased the yield of **2 a** up to 37 % (entry 3). The use of a stronger oxidant such as DDQ led to cleaner reactions, obtaining less side‐products, yet reducing the yield (entry 4). Increasing the ratio of MeOH reduced the amount of **1 a**, or even avoided for its obtention (entries 5–6). Scaling of the reaction by keeping the conditions of entry 4 led also to reduced yields (entry 7). CuCor **3 a** was obtained in 85 % yield by treatment of the free‐base Cor **2 a** with copper acetate. We considered the insertion of copper into **2 a** not only to modify its electronic properties, but also as a synthetic strategy: due to their high electron rich and constrained core, free‐base Cors are prone to deprotonation and exposed to different decomposition pathways. Copper allows for straightforward metalation and demetallation processes, thus increasing the overall yield of the subsequent reactions while protecting the core.

**Scheme 2:**
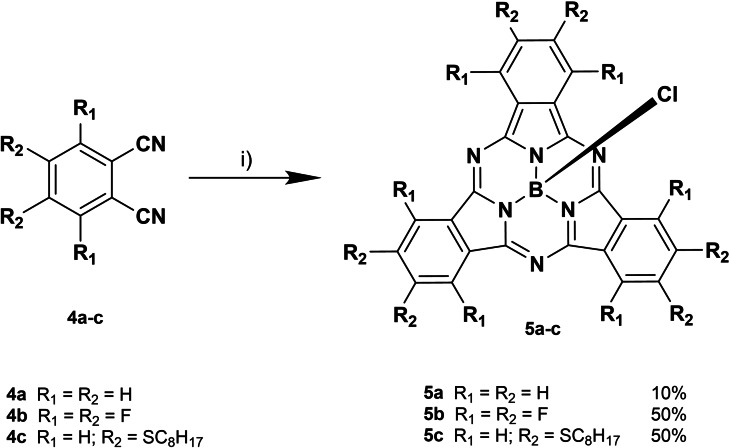
Synthetic route for the obtention of SubPcs **5 a**–**c**. i) BCl_3_ (1.0 M in *p*‐xylene), 120–145 °C, 20–120 min.

**Scheme 3:**
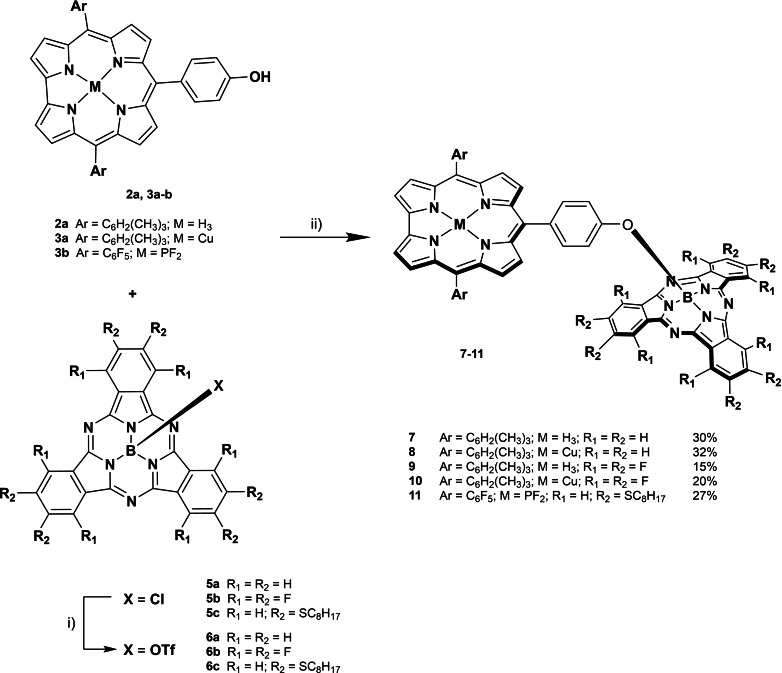
Synthetic route for the obtention of SubPc‐Cor conjugates **7**–**11**. i) AgOTf (1.25 equiv), toluene, 25–60 °C, 10 min–6 h. ii) DIPEA (1.25–2.50 equiv), toluene, 40–80 °C, 18–24 h.

SubPcs **5 a** and **5 b** were synthesized using standard cyclotrimerization conditions of the selected phthalonitriles in the presence of BCl_3_ (Scheme 2). SubPc‐Cor conjugates **7**–**10** were prepared by axial activation of SubPcs **5 a** and **5 b** and subsequent addition of **2 a** and **3 a**, in the presence of diisopropylethylamine (DIPEA), in moderate yields (Scheme 3).

From a global point of view, the reaction took place in milder conditions and faster for **8**>**7**>**10**>**9**, as followed by TLC. The differences in reactivity among the molecular subunits have a direct influence in the yield. For copper derivatives **8** and **10**, the reaction proved to be cleaner than for the free‐base Cor dyads **7** and **9**, without detectable traces of radical or cationic species. In contrast, whereas in the case of the dyads **7** and **8** reactions were completed in a few hours at room temperature, for the dyads **9** and **10** an increase of temperature and higher reaction times were needed. In particular, in the case of dyad **9**, an additional amount of Cor and DIPEA were required in order to complete the reaction. This is a direct reflection of the combination of the minor reactivity presented by the SubPc **5 b** and the decreased availability of the free‐base Cor **2 a**. The latter may be attributed to the protonation of its inner imine nitrogen by the presence of triflic acid in the medium, released during axial substitution reaction of the activated SubPc.

The structures of conjugates **7**–**10** were confirmed by means of nuclear magnetic resonance (NMR), UV‐Vis spectroscopy and mass spectrometry (MS) experiments. For the dyads **7** and **9** comprising free‐base Cors, ^1^H NMR spectra returned a set of signals at δ 8.0‐9.0 ppm corresponding to the β‐pyrrole protons, whereas the two singlets at 2.6 and 1.9 ppm correspond to the *meso‐*aryl‐CH_3_ protons. Additionally, the doublets around 7.6 and 5.7 ppm belong to the phenoxy‐ bridge of the conjugates. In comparison with Cor **2 a**, the phenoxy‐ signals appear upfield, which is a characteristic feature of the direct connection of phenols into SubPc boron atom, due to the presence of an anisotropic ring current coming from the SubPc 14 π‐electron delocalized system. In the case of dyad **7**, multiplets arising from the AA'BB’ system at 9.0 and 8.0 ppm, corresponding to the α*‐* and β*‐*phenyl SubPc protons, are detected. For the CuCor dyads **8** and **10**, the antiferromagnetic coupling developed by those non‐innocent ligands leads to a paramagnetic behavior of the CuCor.^[24]^

The spin coupling effect induced by the complex increases the relaxation rate of the protons, thus not making them visible in our acquisition times. Even though its presence does not allow to record Cor β‐pyrrolic protons, the structure of the conjugates can still be determined from the external phenoxy ring and the *meso*‐aryl‐CH_3_ protons. In the case of dyads **9** and **10** comprising perfluorinated SubPcs, the presence of two multiplets at −136.5 and −147.2 ppm in ^19^F NMR spectra confirmed its inclusion.

On the other hand, we synthesized an additional D–A conjugate switching their electronic roles, by combination of the high electron deficient PCor **3 b** with the electron‐rich thioether SubPc **5 c**, which has been demonstrated to conduct efficient charge transfer in combination with an electronically‐suited partner.^[10a]^ Both PCor **3 b** and SubPc **5 c** were prepared following similar procedures to those described above for their homologues (Scheme 3). In particular for PCor **3 b**, the insertion of phosphorus into Cor **2 b** and subsequent axial substitution with fluoride was performed following a specific approach reported in the literature by Gross and co‐workers.^[25]^ In the same way, SubPc‐Cor **11** was obtained in 27 % yield. Although the reactivity of SubPc **5 c** is quite remarkable, activation with silver triflate was carried out in order to maintain the same conditions and ensure the success of the reaction. Full characterization of **11** by means of NMR, UV‐Vis spectroscopy and MS was performed, revealing similar features as those observed for dyads **7**–**10**.

### Electrochemistry and Spectroelectrochemistry

Cyclic as well as differential pulse voltammograms of the Cor and SubPc references were recorded in dichloromethane with ferrocene as an internal standard (Table 2 and Figure S6.1). **2 a** features three reversible reductions at −0.65, −1.51, and −1.78 V versus Fc/Fc^+^ as well as one oxidation at +0.10 V versus Fc/Fc^+^. A set of two reductions and oxidations are discernable for **3 a** at −0.76 and −1.32 V versus Fc/Fc^+^ as well as +0.21 and +0.93 V versus Fc/Fc^+^. PCor **3 b** features a single reduction and two reversible oxidations at −1.60, +0.64, and +0.94 V versus Fc/Fc^+^. For **5 a**, three reversible reductions are noted at −1.56, −2.02, and −2.27 V versus Fc/Fc^+^. The oxidation sets in at +0.57 V versus Fc/Fc^+^. **5 b** features reductions at −1.05, −1.34, and −1.74 V versus Fc/Fc^+^ complemented by an oxidation at +0.98 V vs. Fc/Fc^+^. Finally, two reversible reductions and a single oxidation are noted for **5 c** at −1.89, −1.45, and +0.56 V versus Fc/Fc^+^. From the aforementioned data, we derive energies for the corresponding charge‐separated states based on the assumption that the Cors are oxidized and the SubPcs are reduced of about 1.66, 1.77, 1.15, 1.26, and 2.09 eV for **7**, **8**, **9**, **10**, and **11** in dichloromethane, respectively. In the case of a reversed Cor^−^⋅‐SubPc^+^⋅ CSS, the energy is 2.16 eV for **11** (Table 2).

**Table 2:** Redox potentials of Cor and SubPc references (in V vs. Fc/Fc^+^) in de‐aerated dichloromethane (0.1 M TBAPF_6_ as electrolyte) at room temperature.

	E_3rd Red_	E_2nd Red_	E_1st Red_	E_1st Ox_	E_2nd Ox_
**2 a**	−1.78	−1.51	−0.65	+0.10	
**3 a**		−1.32	−0.76	+0.21	+0.93
**3 b**			−1.60	+0.64	+0.94
**5 a**	−2.27	−2.02	−1.56	+0.57	
**5 b**	−1.74	−1.34	−1.05	+0.98	
**5 c**		−1.89	−1,45	+0.56	

Differential absorption spectra of the oxidized and/or reduced forms were obtained upon application of a bias voltage to a solution of the respective Cor or SubPc references (Figure S6.2). The corresponding spectra are dominated by the respective ground state bleaching around 420 and 570 nm for Cors and SubPcs, respectively. The one‐electron oxidized form of **2 a** features differential maxima at 394, 454, 600, 634, and 670, followed by minima at 415, 433, 567, and 610 nm. For **3 a**, the one‐electron oxidized form reveals 389, 480, 590, and 660 nm maxima as well as 433 and 532 nm minima. **3 b** features maxima at 378, 394, 430, and 607 nm, next to minima at 409 and 580 nm upon oxidation. Comparable signals arise upon reduction except for a shifted 400 nm maximum, which replaces the former at 394 nm. One‐electron reduction of **5 a** is accompanied by maxima and minima at 598 and 510/564 nm, respectively. For the one‐electron reduced form of **5 b** we noted 600 and 650 nm maxima. Minima in this particular case were found at 455, 522, and 570 nm. Finally, maxima at 634 and 687 nm, and minima at 384, 594, 727, and 773 nm are found upon reduction of **5 c**. Here, one‐electron oxidation yields maxima around 460 and 660 nm as well as minima at 376, 600, 727, and 773 nm.

### Steady‐State Absorption & Fluorescence Spectroscopy

**Figure 2:**
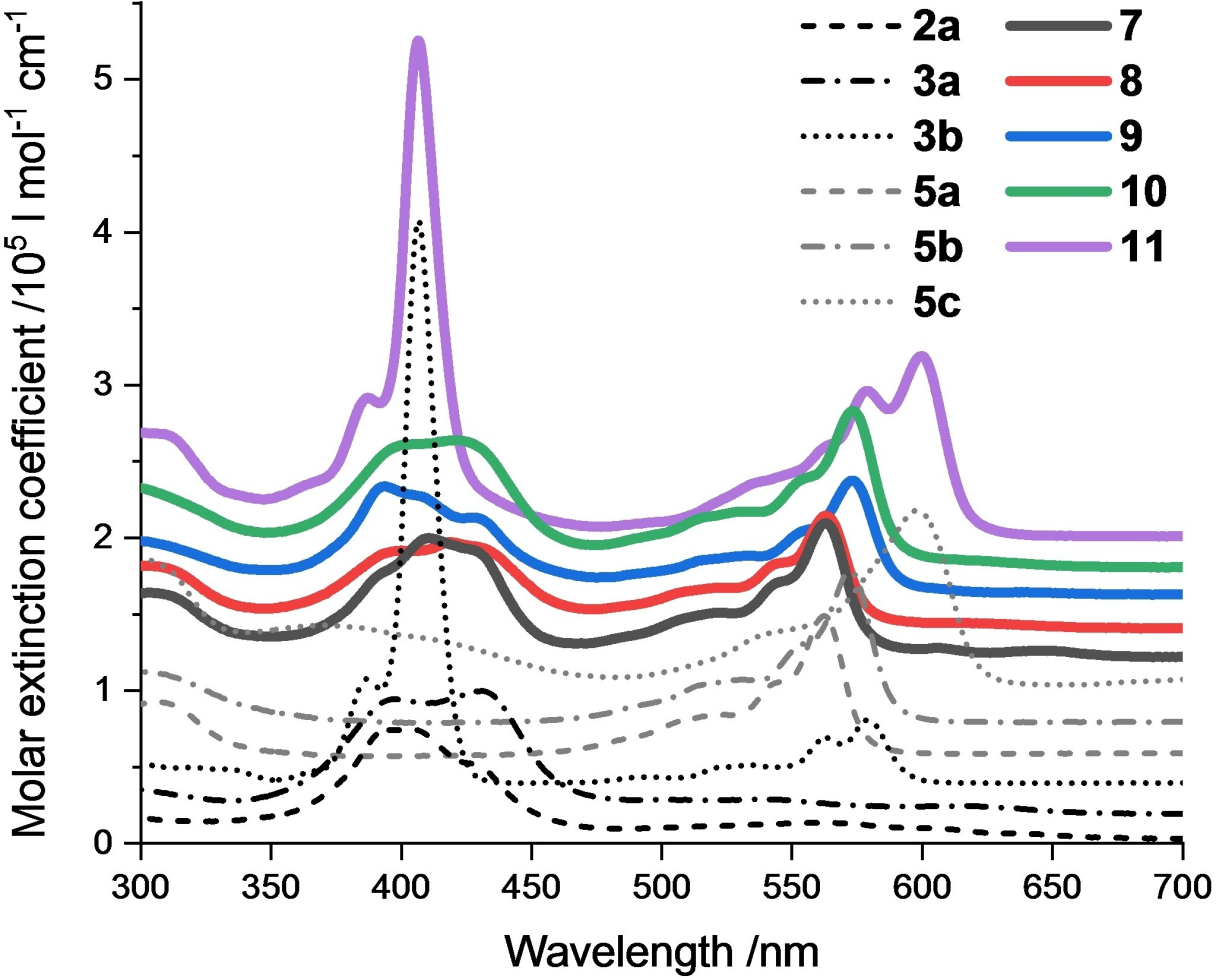
Steady‐state absorption spectra of the Cor and SubPc references, as well as the conjugates in toluene at room temperature (spectra shifted by 2×10^4^ l mol^−1^ cm^−1^ for visibility).

Steady‐state absorption spectra have been recorded in toluene and benzonitrile (Figures 2 and S7.1). The absorption spectra of the Cor references consist of Soret‐band absorptions with maxima at 395/403, 398/431, and 387/406 nm for **2 a**, **3 a**, and **3 b**, respectively, and weaker Q‐band absorptions between 500 and 650 nm. For **3 b**, the lowest‐energy absorption is at shorter wavelengths, that is, at 579 nm. For SubPcs, the Soret‐band absorptions are typically found below ∼350 nm, while the Q‐band absorptions are noted between 450 and 600 nm. The latter maximizes at 562, 572, and 598 nm for **5 a**, **5 b**, and **5 c**, respectively.

When turning to the conjugates, their absorptions are best described as close matches of the corresponding references both in terms of intensity and maxima. For example, the Q‐band absorption maxima for **7** and **8** as well as for **9** and **10** are at 563 and 573 nm, respectively. Hence, little to no ground state interactions are assumed between the different constituents in the conjugates.

**Figure 3:**
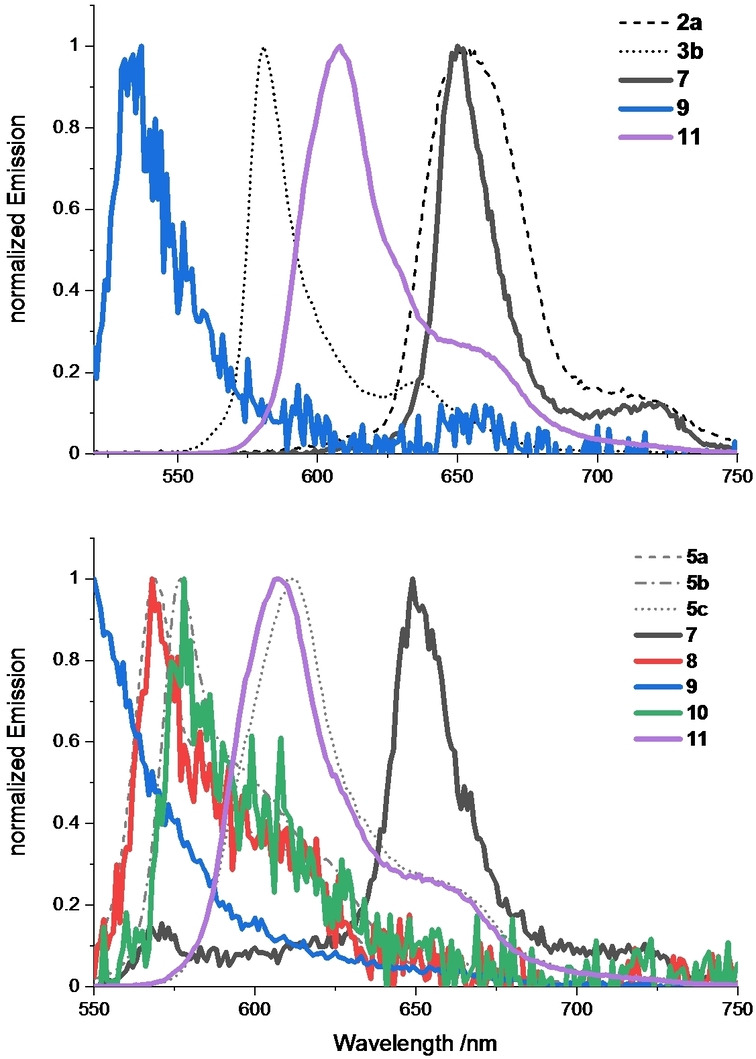
Normalized steady‐state fluorescence spectra of the Cor and SubPc references as well as the conjugates in toluene at room temperature upon 420 (top) or 540 nm (bottom) photoexcitation. H_2_TPP and **5 b** were used as references for Cor‐ and SubPc‐centered fluorescence, respectively.[^27^, ^28^]

Fluorescence spectra were recorded in toluene and benzonitrile (Figures 3 and S7.2). Here, maxima were found at 652/581 and 569/577/611 nm for the Cor references, that is, **2 a**/**3 b** and for the SubPc references, that is, **5 a/5 b**/**5 c** while **3 a** is strictly non‐fluorescent. The fluorescence of the conjugates is generally much weaker relative to their respective references. For example, **7** shows mostly Cor‐based fluorescence regardless of the excitation wavelength. Only a fraction of SubPc‐centered fluorescence yet occurs upon SubPc photoexcitation and it is stronger in benzonitrile than in toluene. As such, an energy transfer towards the Cor is likely to take place. In **9**, emission occurs at 534 nm and is stronger upon excitation of the SubPc. The maximum appears blue‐shifted versus the corresponding SubPc absorption at 573 nm and therefore cannot be assigned clearly. Both CuCor conjugates, that is, **8** and **10**, show exclusively SubPc‐centered fluorescence upon photoexcitation into the corresponding Q‐bands.

Overall, the fluorescence quantum yields of the conjugates **7**–**10** were much lower with values at around 0.01 relative to the references with 0.1–0.2 (Table 3). Alternative singlet excited state deactivation pathways are likely to be involved. In contrast, **11** features solely SubPc‐centered fluorescence regardless of the excitation wavelength, indicating energy transfer towards the SubPc. Also, no quenching was observed in this case.

**Table 3:** Fluorescence maxima λ_max_ (in nm) and quantum yields φ_Fl_ of references and dyads in toluene at room temperature.

	λ_max, 420 nm Exc._	Φ_Fl, 420 nm Exc._	λ_max, 540 nm Exc_	Φ_Fl, 540 nm Exc._
**2 a**	652	0.10		
**3 a**	–	–		
**3 b**	581	0.33		
**5 a**			569	0.17
**5 b**			577	0.17
**5 c**			611	0.09
**7**	650	0.02	570/650	0.01
**8**	–	–	570	<0.01
**9**	534	<0.01	<550	<0.03
**10**	–	–	578	<0.01
**11**	608	0.13	608	0.14

### Femtosecond Transient Absorption Spectroscopy

Femtosecond transient absorption spectroscopy of the different Cor and SubPc references was performed in toluene upon photoexcitation at 420 and 550 nm, respectively. For **2 a**, the initial population of the second singlet excited state (S_2_) is followed by internal conversion and vibrational relaxation with 6.1 and 74 ps, respectively (Figure S8.1). Ultimately, this relaxed first singlet excited state (S_1_) undergoes intersystem crossing (ISC) to populate the triplet excited state (T_1_) within 1.4 ns. The transient spectra are mostly defined by a strong ground state bleaching around 420 nm as well as two maxima at 1100 and 1200 nm. Placing a heavy atom with an open shell character drives a much faster singlet‐to‐triplet conversion in **3 a** (Figure S8.2). S_2_, which is characterized by a broad 600 nm maximum, decays with 1.9 ps to form S_1_, which undergoes ISC with 11 ps. A final species emerges with 159 ps lacking any ground‐state bleaching. Hence, we propose the population of a solely Cu‐centered species as Cor does not seem to be excited. In contrast to the other Cors, spectra of **3 b** are mostly defined by the Q‐band bleaching at 550–600 nm (Figure S8.3). The transient species, which relate to the free‐base Cor, give rise to lifetimes are 0.5 and 14 ps, as well as 2.0 ns for the S_2_, unrelaxed S_1_, and relaxed S_1_, respectively. Once photo‐excited, **5 a** undergoes vibrational relaxation from the unrelaxed to the relaxed S_1_. Both states are characterized by strong ground state bleaching around 560 nm, a maximum around 620 nm, and shoulders at 950 and 1100 nm. Their lifetimes are 9.1 ps and 1.2 ns, respectively, and the corresponding product is T_1_ (Figure S8.4).

A similar deactivation was noted for **5 b** with its ground state bleaching at around 570 nm. Here, the lifetimes are 14 ps and 1.0 ns (Figure S8.5). Finally, **5 c** initially undergoes vibrational relaxation followed by ISC to afford T_1_, which is defined by its 598 nm bleaching as well as 483 and 630–655 nm maxima. Lifetimes are 33 and 781 ps for the S_1_s, which feature 470, 692, and 910 nm maxima along with the 600 nm bleaching (Figure S8.6).

**Figure 4:**
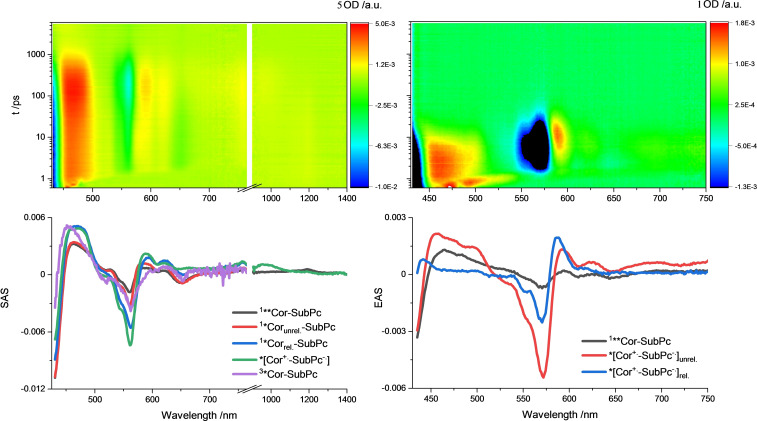
Differential absorption of **7** upon femtosecond flash photolysis with excitation at 420 nm in toluene (top left) and corresponding species associated spectra (bottom left), and differential absorption of **9** upon excitation at 420 nm in benzonitrile (top right) and corresponding evolution associated spectra (bottom right).

**Figure 5:**
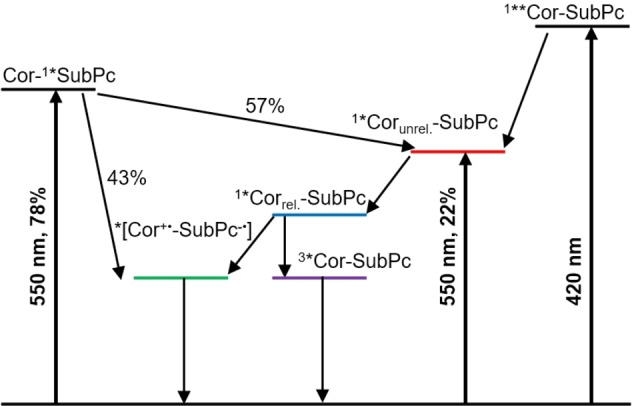
Kinetic models used for the analysis of femtosecond‐resolved differential absorption of **7** in toluene.

Next, the four conjugates were photo‐excited into the Cor Soret‐band at 420 nm and the SubPc Q‐bands at 550 nm either in toluene or benzonitrile. Co‐excitation of both constituents was considered upon 550 nm photoexcitation. The Cor‐centered S_2_ is initially populated upon photoexcitation of **7** at 420 nm in toluene. It features characteristic maxima at 460 and 1200 nm as well as 420 nm ground state bleaching (Figure 4). Subsequent conversion into the unrelaxed S_1_ and relaxed S_1_ occurs with 6.1 and 74 ps, respectively. Two states, that is, the Cor‐centered T_1_ and charge‐separated state (CSS), are formed in parallel from the relaxed S_1_ with 161 ps (Table 4). Population of a charged species is supported by ground‐state bleaching at 420 and 560 nm as well as maxima at 590 and 620 nm, which fit the fingerprints of the one‐electron oxidized **2 a** obtained in the spectroelectrochemical measurements (see above). The lifetime is 493 ps. Photoexcitation of **7** at 550 nm leads to 78 % population of the SubPc S_1_ and 22 % population of the unrelaxed Cor S_1_ (Figure S8.7). This ratio was calculated from the relative extinction coefficients at 550 nm. The SubPc‐centered S_1_, with its 560 nm bleaching and broad 600–630 nm maximum, decays, on one hand, into the CSS and transfers energy, on the other hand, to the unrelaxed Cor S_1_ with 1.5 ps. Here, the relative ratio is 43 % to 57 %. The unrelaxed Cor S_1_ then undergoes relaxation with 74 ps and finally decays either into the CSS or Cor‐centered T_1_ within 180 ps. The lifetime of 534 ps is similar to that derived in the 420 nm experiments. The models used in toluene are summarized in Figure 5. In benzonitrile rather than toluene, an analysis of the measurements suggests population of the CSS from the unrelaxed Cor S_1_ of 30 % as well as 80 % from the relaxed Cor S_1_. The remaining 20 % transform into the Cor‐centered T_1_ (Figures S8.9 & S8.10). Lifetimes of 6.1, 5.3, 24 and 90 ps were obtained upon excitation at 420 nm for the Cor‐based S_2_, unrelaxed S_1_, CSS, and relaxed S_1_, respectively. In the 550 nm experiments, going through a SubPc‐centered S_1_ instead of the Cor‐centered S_2_, the lifetimes are 1.8, 5.3, 24, and 104 ps. Please note the additional population of the CSS from the SubPc S_1_ on the order of 43 %.

**Table 4:** Excited state lifetimes of singlet species preceding the CSSs and the CSSs (in ps) upon excitation of **7**–**10** with 420 and 550 nm (in brackets).

	Toluene		Benzonitrile	
	Singlet(s)	CSS	Singlet(s)	CSS(s)
**7**	161	493	5.3/90	24
	(1.5/180)	(534)	(1.8/5.3/104)	(24)
**8**	1.8	22	1.4	4.7/20
	(2.2)	(20)	(3.0/1.0)	(7.2/35)
**9**	3.0	22	2.6	6.1/21
	(1.3/5.3)	(24)	(1.1)	(8.3/21)
**10**	1.7	23	0.9	4.0/20
	(0.9/5.6)	(22)	(0.9/4.0)	(3.6/20)

Compound **9** shows a much more rapid deactivation in toluene upon 420 nm photoexcitation, likely due to a higher/lower driving force for charge separation/recombination compared to **7**. The CSS decays, for example, with 22 ps after being formed directly from the Cor S_2_ with 3.0 ps (Figure S8.12). Notable is the fact that the SubPc‐centered S_1_ populates the CSS with a 90 % efficiency upon 550 nm photoexcitation. 10 % go into an energy transfer channel to the co‐excited Cor S_1_, which, in turn, yields the CSS. Upon charge recombination, only the SubPc‐centered T_1_ is detected. The corresponding lifetimes are 1.3, 5.3, and 24 ps for the SubPc‐centered S_1_, Cor‐centered S_1_ and CSS, respectively (Figure S8.13). In benzonitrile, three species are found for **9**. The short lifetimes are 2.6, 6.4, and 21 ps (Figure 4). The initially populated Cor S_2_ decays into a CSS, featuring both ground‐state bleachings.

A third and final species – defined by the intense SubPc bleaching as well as an even more pronounced maximum at 587 nm – emerges in benzonitrile in contrast to toluene. Hence, we conclude the formation of a second CSS, which becomes visible due to the slowed down relaxation of the solvent. Here, the model is summarized as follows: Cor photoexcitation results in the population of its S_2_, which transforms via the solvent‐unrelaxed CSS to the solvent‐relaxed CSS. Upon 550 nm photoexcitation the S_1_ lifetime is 1.1 ps, while the two CSSs decay with 8.2 and 21 ps (Figure S8.15). Minor amounts of Cor and SubPc T_1_s are also noted upon charge‐recombination. They are exclusively populated from the solvent‐relaxed CSS, and they are populated in parallel. Models of experiments upon 550 nm excitation consider co‐excitation into both the Cor and SubPc S_1_. However, these two species had to be treated as a single state in the model as their short lifetimes proved indistinguishable.

When photoexcited at 420 nm, the CuCor‐based **8** forms initially its Cor‐centered S_2_ state. It is characterized by ground state bleaching as well as a 600 nm maximum. This is followed by the CSS, which, in turn, populates both T_1_s, namely that of Cor and SubPc (Figure S8.17). Charge‐separation and recombination occur in **8** at the picosecond timescale with 1.8 and 22 ps. Finally, Cor T_1_ lives for 284 ps, a value that is in sound agreement with the lifetime of the reference. Analysis of the data obtained upon 550 nm photoexcitation yields both the Cor and SubPc S_1_, but due to their indistinguishable lifetimes these again had to be treated as a single species in the model. From this, the CSS and the two T_1_s, which are formed in parallel, follow in succession. The respective lifetimes are 2.2, 20, and 223 ps, respectively (Figure S8.18). Similar models were utilized for the measurements in benzonitrile. Here, the CSS was replaced by the solvent unrelaxed CSS and relaxed CSS (Figures S8.20 & S8.21). Lifetimes of 1.4, 4.7, 20, and 383 ps were obtained for the Cor S_2_, unrelaxed CSS, relaxed CSS, and Cor T_1_, respectively, upon 420 nm photoexcitation. Upon 550 nm photoexcitation, the unrelaxed CSS is formed from the Cor‐centered S_1_ with 3.0 ps and from the SubPc S_1_ with 1.0 ps. On longer timescales, the unrelaxed CSS, the relaxed CSS, and the Cor T_1_, which were formed step‐by‐step, feature lifetimes of 7.2, 35 and 257 ps, respectively.

Compound **10** reveals deactivations similar to those summarized for all the other conjugates (Figures S8.23–28). As a matter of fact, in toluene and upon 420 nm photoexcitation, lifetimes of 1.7, 23, and 294 ps relate to Cor S_2_, CSS and Cor T_1_, respectively. Analysis of the 550 nm photoexcitation data resulted in lifetimes of 0.9, 5.6, 22, and 346 ps. Responsible for these lifetimes are the Cor S_1_ (as combined S_1_ in model), SubPc S_1_, CSS and Cor T_1_. Early on, the S_1_ states could not be separated. As such, the combined S_1_ populates the CSS with 16 % and the SubPc S_1_ with 84 %. It is the latter, which turns into the CSS. Measurements in benzonitrile necessitate the replacement of one CSS with two CSSs, namely an unrelaxed CSS and a relaxed CSS. T_1_ signatures were invisible upon 420 nm photoexcitation. Lifetimes of 0.9, 4.0, and 20 ps correspond in **10** to the Cor S_2_, unrelaxed CSS, and relaxed CSS. 0.9, 4.0, 3.6, and 20 ps were the dynamics, which were obtained for Cor S_1_ (as combined S_1_ in model), SubPc S_1_, unrelaxed CSS, and relaxed CSS upon 550 nm photoexcitation. For the minor amounts of T_1_s in **10** a 332 ps lifetime was derived (Table 4).

Finally, **11** initially forms the unrelaxed SubPc‐centered S_1_ regardless of the excitation wavelength or solvent, which supports the notion of a quantitative energy transfer from the Cor to the SubPc (Figures S8.29–32). Subsequent species are the relaxed SubPc S_1_ and, ultimately, the SubPc T_1_. The S_1_‐lifetimes are 3.8 ps/1.5 ns and 3.3 ps/1.0 ns upon excitation at 420 and 550 nm in toluene, respectively. Experiments in benzonitrile yield comparable lifetimes.

## Conclusion

Five different electron donor–acceptor conjugates involving corroles (Cors) and subphthalocyanines (SubPcs) connected through a short molecular linker have been prepared. A synthetic route has been developed to covalently link Cors and SubPcs via the Cors *meso‐*position and prior activation of the SubPcs axial position. For Cors decorated with mesityl units, an effective charge separation has been detected, generating different Cor^+^‐SubPc^−^ charge‐separated states (CSS). Although copper was incorporated into Cors to enhance the resulting acceptor strength, similarly short CSS lifetimes were observed. Remarkably, by using the free base Cor and the hydrogen‐substituted SubPc, a 25‐fold higher relaxation time was found compared to the rest. It is most likely that the driving force, which is located in the Marcus inverted region, dictates this acceleration. Finally, **11** does not feature a separation of charges upon excitation in contrast to **7**–**10**. However, absorption and fluorescence allow for a significant overlap with each other, so the energy absorbed by the Cor is emitted and fully reabsorbed by the SubPc. This and the extended absorption range, which resulted from the combination of both chromophores, enhances its role as an antenna, especially in combination with structurally and energetically complementary electroactive building blocks such as fullerenes.

## Experimental Section

### Synthetic procedures

Dipyrromethanes **1 a**–**b**,^[22]^ corroles **2 a**–**b**,^[23]^ **3 b**,[^25^, ^26^] phthalonitrile **4 c** ^[29]^ and subphthalocyanines **5 a**–**c**[^29^, ^30^] were prepared as described in the literature.

### General procedure for the synthesis of *trans*‐A_2_B corroles

To a 250 mL round‐bottom flask equipped with a magnetic stirrer, 4‐Hydroxybenzaldehyde (0.50 mmol) and dipyrromethane (1.00 mmol) were dissolved in MeOH (50 mL). Once upon homogenization, a HCl solution (52.5 mL, 0.55 M) was added dropwise and stirred in dark conditions at 25 °C for 2 h. The mixture was extracted with CHCl_3_ and the organic phase was washed with H_2_O (2×50 mL), brine (1×50 mL) and dried over Na_2_SO_4_. After filtration and solvent removal by vacuum distillation, CHCl_3_ (250 mL) and *p*‐chloranil (1.20 mmol) were added, and the solution was stirred in dark conditions at 25 °C for 16 h. Hydrazine (0.1 mL) was added and solvent was removed by vacuum distillation. The resulting solid was subjected to column chromatography on silica gel.

**10‐(4‐hydroxyphenyl)‐5,15‐bis(1,3,5‐trimethylphenyl)corrole (2 a)**: CH_2_Cl_2_ as eluent. Yield: 37 %, purple solid. ^1^H NMR (400 MHz, CDCl_3_): *δ* (ppm)=8.87 (d, ^3^ *J*_H‐H_= 4.4 Hz, 2H), 8.48 (m, 4H), 8.31 (d, ^3^ *J*_H‐H_=4.0 Hz, 2H), 8.01 (d, ^3^ *J*_H‐H_=8.4 Hz, 2H), 7.18 (d, ^3^ *J*_H‐H_=8.4 Hz, 2H), 2.60 (s, 6H), 1.91 (s, 12H). ^13^C NMR (126 MHz, CDCl_3_): *δ* (ppm)=143.3, 142.7, 140.9, 140.1, 139.3, 135.6, 135.1, 131.6, 130.0, 128.3, 128.1, 126.7, 126.3, 125.6, 120.6, 118.9, 115.1, 114.2, 22.7, 21.5. MS (MALDI‐TOF, DCTB): *m/z*=625.4 [M−H]^+^, 626.4 [M]^+^. HRLSI‐MS (APCI+): *m/z* Calcd for [(C_43_H_38_N_4_O)H]: 627.3118; Found: 627.3126 [M]H^+^+626.3057 [M]^+^ . UV‐Vis (Toluene): *λ*_max_ (nm) (log *ϵ* (dm^3^ mol^−1^ cm^−1^))=641 (3.61), 607 (3.83), 566 (4.12), 428 (4.78), 410 (4.69), 410 (4.87).

**5,15‐bis(pentafluorophenyl)‐10‐(4‐hydroxyphenyl)corrole (2 b)**: CH_2_Cl_2_ as eluent. Yield: 14 %, red solid. ^1^H NMR (400 MHz, CDCl_3_): *δ* (ppm)=9.12 (d, ^3^ *J*_H‐H_=4.4 Hz, 2H), 8.71 (m, 4H), 8.57 (d, ^3^ *J*_H‐H_=4.0 Hz, 2H), 8.05 (d, ^3^ *J*_H‐H_=8.4 Hz, 2H), 7.22 (d, ^3^ *J*_H‐H_=8.4 Hz, 2H). MS (ESI+): *m/z*=723.6 [M+H]^+^. UV/Vis (CHCl_3_) *λ*_max_ (nm)=412, 560, 613, 639.

### Complexation of corroles

**10‐(4‐hydroxyphenyl)‐5,15‐bis(1,3,5‐trimethylphenyl)corrolato‐Cu (3 a)**: To a 50 mL round‐bottom flask equipped with a magnetic stirrer, Cor **2 a** (0.069 mmol) was added over CHCl_3_ (10 mL) and stirred at 40 °C. Cu(AcO)_2_ (0.550 mmol) dissolved in hot MeOH (5 mL) was added and the solution was further stirred at 40 °C for 15 minutes. The solvent was removed by vacuum distillation and the resulting solid was subjected to column chromatography on silica gel using a mixture of CH_2_Cl_2_/Heptane 5 : 1 as eluent. Yield: 85 %, brown solid. ^1^H NMR (300 MHz, CDCl_3_): *δ* (ppm)=7.96 &bk>(d, ^3^ *J*_H‐H_=8.4 Hz, 2H), 7.14 (d, ^3^ *J*_H‐H_=8.4 Hz, 2H), 2.41 (s, 6H), 2.01 (s, 12H). MS (MALDI‐TOF, DCTB): *m/z*=686.3 [M]^+^. HRLSI‐MS (APCI+): *m/z* Calcd for [(C_43_H_35_N_4_OCu)H]: 687.2180; Found: 628.2200. UV‐Vis (Toluene): *λ*_max_ (nm) (log *ϵ* (dm^3^ mol^−1^ cm^−1^))=621 (3.45), 538 (3.80), 421 (4.76), 392 (4.69).

**5,15‐bis(pentafluorophenyl)‐10‐(4‐hydroxyphenyl)corrolato‐P(fluorine)_2_ (3 b)**: To a 50 mL round‐bottom flask equipped with a magnetic stirrer, Cor **2 b** (0.2 mmol) was added over pyridine (11 mL), degassed and warmed up to reflux. An excess of PCl_3_ (20 mmol) was added and the solution was stirred at reflux for 30 minutes. Upon complete consumption of the starting material, water was added dropwise till the formation of a precipitate. The resulting solid was filtered, washed with water, recovered with CHCl_3_ and evaporated to dryness. The mixture was dissolved in CH_2_Cl_2_ (30 mL) and added to a plastic flask equipped with a magnetic stirrer. HF 50 % (11 mL) was added and the mixture was stirred vigorously in dark conditions for 16 h. The mixture was extracted with CHCl_3_ and the organic phase was washed with H_2_O (2×50 mL), NaHCO_3_ (1×50 mL) and dried over Na_2_SO_4_. After filtration and solvent removal by vacuum distillation, the resulting solid was subjected to column chromatography on silica gel using a mixture of CHCl_3_/MeOH 9 : 1 as eluent. Yield: 73 %. Bright red solid. ^1^H NMR (400 MHz, CDCl_3_) *δ* (ppm): 9.46 (dd, ^4^ *J*_H‐P_=3.2 Hz, ^3^ *J*_H‐H_=4.4 Hz, 2H), 9.06 (t, ^3^ *J*_H‐H/H‐P_=4.4 Hz, 2H), 9.01 (m, 4H), 8.07 (d, ^3^ *J*_H‐H_=8.4 Hz, 2H), 7.25 (d, *J*=8.4 Hz, 2H). ^19^F NMR (376 MHz, CDCl_3_) *δ* (ppm): −37.5 (d, ^1^ *J*_P‐F_=810 Hz 2F,), −136.3 (m, 4F), −151.7 (m, 2F), −161.1 (m, 4F). ^31^P NMR (162 MHz, CDCl_3_) *δ* (ppm): −182.1 (t, ^1^ *J*_P‐F_=810 Hz, 1P). HRLSI‐MS (APcI): m/z Calcd for [(C_37_H_13_F_12_N_4_OP)]: 788.0635; Found: 788.0635. UV‐Vis (CHCl_3_): *λ*_max_ (nm) (log *ϵ* (dm^3^ mol^−1^ cm^−1^))=582 (4.62), 565 (4.51), 536 (sh), 407 (5.58), 386 (4.83).

### Synthesis of chloro‐substituted subphthalocyanines

**Chloro‐subphthalocyaninateboron (5 a)**: To a 25 ml round‐bottom two‐neck flask equipped with a reflux condenser and magnetic stirrer, a 1.0 M solution of BCl_3_ in *p*‐xylene (8 mL) was added to 3,4,5,6‐tetrafluorophthalonitrile (8 mmol) under argon atmosphere. The mixture was stirred and heated to reflux (145 °C) for 40 mins. The crude was cooled down to room temperature and flushed with argon. After evaporation of *p*‐xylene under reduced pressure, the reaction flask was filled with heptane and sonicated for 30 mins. The dark solid was vacuum filtrated and washed with heptane until no color was observed, and then was further washed with methanol. The resulting solid was recovered with chloroform and dried. Yield: 15 %, dark purple solid. ^1^H NMR (300 MHz, CDCl_3_): *δ* (ppm)=8.68 (s, 6H), 7.83 (s. 6H). UV‐vis (CHCl_3_): *λ*_max_ (nm) (log *ϵ* (dm^3^ mol^−1^ cm^−1^))=565 (4.4), 529 (sh), 308 (4.1).

**Chloro‐1,2,3,4,8,9,10,11,15,16,17,18‐dodecafluorosubphthalocyani‐nateboron (5 b)**: To a 25 ml round‐bottom two‐neck flask equipped with a reflux condenser and magnetic stirrer, a 1.0 M solution of BCl_3_ in *p*‐xylene (5 mL) was added to 3,4,5,6‐tetrafluorophthalonitrile (5 mmol) under argon atmosphere. The mixture was stirred and heated to reflux (145 °C) for 2 h. The crude was cooled down to room temperature and flushed with argon. After evaporation of *p*‐xylene under reduced pressure, the resulting dark solid was subjected to column chromatography on silica gel using toluene/heptane 1 : 1 as eluent. Yield: 50 %, bright magenta solid. ^19^F NMR (470 MHz, CDCl_3_): *δ* (ppm)=−137.0 (6F), −147.7 (6F). ^13^C NMR (75.5 MHz, CDCl_3_): *δ* (ppm)=146.9, 144.5 (m), 141.0 (m), 115.0. MS (MALDI‐TOF, DCTB): *m/z*=646.0 [M]^+^. UV‐vis (CHCl_3_): *λ*_max_ (nm) (log *ϵ* (dm^3^ mol^−1^ cm^−1^))=574 (4.6), 556 (sh), 530 (sh), 311 (4.3), 277 (4.0).

**Chloro‐2,3,9,10,16,17‐hexa(octylthio)subphthalocyaninateboron (5 c)**: To a 25 ml round‐bottom two‐neck flask equipped with a reflux condenser and magnetic stirrer, a 1.0 M solution of BCl_3_ in *p*‐xylene (2.4 mL) was added to 4,5‐bis(octylthio)phthalonitrile (0.24 mmol) under argon atmosphere. The mixture was stirred and heated (120 °C) for 20 mins. The crude was cooled down to room temperature and flushed with argon. After evaporation of *p*‐xylene under reduced pressure, the resulting dark solid was subjected to a short column chromatography on silica gel using heptane/ethyl acetate 3 : 1 as eluent. Yield: 60 %, dark blue viscous solid. ^1^H NMR (300 MHz, CDCl_3_): δ (ppm)=8.08 (s, 6H), 3.25 (m, 12H), 1.86 (m, 12H), 1.60 (m, 12H), 1.33 (m, 48H), 0.87 (m, 18H). UV‐vis (CHCl_3_): *λ*_max_ (nm) (log *ϵ* (dm^3^ mol^−1^ cm^−1^))=603 (4.7), 555 (sh), 413 (4.1), 3.87 (4.1), 304 (4.2).

### General procedure for the synthesis of subphthalocyanine‐corrole dyads

To a 10 mL Schlenk flask equipped with a magnetic stirrer, dry toluene (3 mL) was added to a mixture of the corresponding SubPc (0.116 mmol) and AgOTf (0.144 mmol), under argon atmosphere. The mixture was stirred at 25–60 °C for 2–6 h (10 min for SubPc‐Cor dyad **11**). The corresponding Cor (0.116 mmol) and DIPEA (0.144 mmol for SubPc‐Cor dyads **7**, **8**, **9** and **11**; 0.174 mmol for SubPc‐Cor dyad **10**) were then added and the suspension stirred at 40–80 °C for 24 h (18 h for SubPc‐Cor dyad **11**). The solvent was removed by vacuum distillation and the resulting solid was subjected to column chromatography on silica gel. Each compound was further purified by size exclusion chromatography in CHCl_3_.

**Subphthalocyanine‐corrole dyad 7**: Axial activation temperature and reaction time: 40 °C, 2 h. Axial substitution temperature: 40 °C. Toluene/THF 20 : 1 as eluent. Yield: 30 %, dark purple solid. ^1^H NMR (300 MHz, CDCl_3_): δ (ppm)=8.95 (m, 6H), 8.85 (d, ^3^ *J*_H‐H_= 4.2 Hz, 2H), 8.42 (d, ^3^ *J*_H‐H_=4.5 Hz, 2H), 8.29 (d, ^3^ *J*_H‐H_=4.2 Hz, 2H), 8.25 (d, ^3^ *J*_H‐H_=4.8 Hz, 2H), 7.96 (m, 6H), 7.54 (d, ^3^ *J*_H‐H_=8.1 Hz, 2H), 5.75 (d, ^3^ *J*_H‐H_=8.1 Hz, 2H), 2.61 (s, 6H), 1.91 (s, 12H). ^11^B NMR (96 MHz, CDCl_3_): δ (ppm)=−14.53. ^13^C NMR (75 MHz, CDCl_3_): δ (ppm)=152.2, 151.5, 139.3, 137.6, 135.7, 135.0, 134.8, 131.1, 131.0, 129.9, 129.9, 128.0, 126.3, 122.3, 122.2, 117.7, 115.0, 109.6, 22.7, 21.1. MS (APCI+): *m/z*=1021.43 [M]^+^, 627.31 [Cor+H]^+^. HRLSI‐MS (APCI+): *m/z* Calcd for [(C_67_H_49_BN_10_O)H]: 1021.4267; Found: 1021.4291. UV‐Vis (Toluene): *λ*_max_ (nm) (log *ϵ* (dm^3^ mol^−1^ cm^−1^))=640 (3.87), 606 (3.99), 563 (4.95), 544 (sh), 518 (4.49), 427 (4.84), 410 (4.92), 303 (4.67).

**Subphthalocyanine‐corrole dyad 8**: Axial activation temperature and reaction time: 40 °C, 2 h. Axial substitution temperature: 40 °C. Toluene/THF 20 : 1 as eluent. Yield: 32 %, brown solid. ^1^H NMR (300 MHz, CDCl_3_): δ (ppm)=8.87 (m, 6H), 7.91 (m, 6H), 7.31 (d, ^3^ *J*_H‐H_=8.4 Hz, 2H), 5.49 (d, ^3^ *J*_H‐H_=8.4 Hz, 2H), 2.40 (s, 6H), 2.03 (s, 12H). ^11^B NMR (96 MHz, CDCl_3_): δ (ppm)=−14.73. ^13^C NMR (75 MHz, CDCl_3_): δ (ppm)=153.4, 151.4, 137.5, 131.0, 129.9, 129.0, 128.9, 128.2, 128.1, 122.3, 120.9, 118.3, 21.2, 19.8. MS (MALDI‐TOF, DCTB): *m/z*=1080.3 [M]^+^. HRLSI‐MS (APCI+): *m/z* Calcd for [(C_67_H_46_BCuN_10_O)H]: 1081.3329; Found: 1081.3339. UV‐Vis (Toluene): *λ*_max_ (nm) (log *ϵ* (dm^3^ mol^−1^ cm^−1^))=622 (3.52), 5.63 (4.87), 545 (4.61), 516 (4.41), 416 (4.76), 3.99 (4.71), 300 (4.64).

**Subphthalocyanine‐corrole dyad 9**: Axial activation temperature and reaction time: 60 °C, 6 h. Axial substitution temperature: 80 °C. Toluene/heptane 2 : 1 as eluent. Yield: 15 %, dark magenta solid. ^1^H NMR (300 MHz, CDCl_3_): δ (ppm)=8.86 (d, ^3^ *J*_H‐H_=4.2 Hz, 2H), 8.44 (d, ^3^ *J*_H‐H_=4.8 Hz, 2H), 8.30 (d, *J*=4.2 Hz, 2H), 8.21 (d, ^3^ *J*_H‐H_=4.8 Hz, 2H), 7.59 (d, ^3^ *J*_H‐H_=8.4 Hz, 2H), 5.69 (d, ^3^ *J*_H‐H_=8.4 Hz, 2H), 2.60 (s, 6H), 1.89 (s, 12H). ^11^B NMR (160 MHz, CDCl_3_): δ (ppm)=−14.74. ^13^C NMR (126 MHz, CDCl_3_): δ (ppm)=152.2, 152.0, 144.3 (m), 141.1 (m), 139.3, 137.6, 135.7, 135.0, 134.8, 131.2, 131.0, 129.9, 126.4, 122.3, 117.7, 115.1 (m), 115.0, 109.6, 22.7, 21.1. ^19^F NMR (471 MHz, CDCl_3_): δ (ppm)=−136.50 (6F), −147.24 (6F). MS (MALDI‐TOF, DCTB): *m/z*=1235.3 [M−H]^+^, 1236.3 [M]^+^, 625.4 [Cor‐H]^+^, 626.4 [Cor]^+^. HRLSI‐MS (APCI+): *m/z* Calcd for [(C_67_H_37_BF_12_N_10_O)H]: 1237.3137; Found: 1237.3143. UV‐Vis (Toluene): *λ*_max_ (nm) (log *ϵ* (dm^3^ mol^−1^ cm^−1^))=638 (3.28), 606 (3.67), 573 (4.80), 553 (sh), 528 (3.32), 428 (4.79), 408 (4.87), 394 (sh).

**Subphthalocyanine‐corrole dyad 10**: Axial activation temperature and reaction time: 60, 6 h. Axial substitution temperature: 65 °C. Toluene/heptane 2 : 1 as eluent. Yield: 20 %, dark magenta solid. ^1^H NMR (300 MHz, CDCl_3_): δ (ppm)=7.33 (d, ^3^ *J*_H‐H_=8.4 Hz, 2H), 5.50 (d, ^3^ *J*_H‐H_=8.4 Hz, 2H), 2.41 (s, 6H), 2.04 (s, 12H). ^11^B NMR (96 MHz, CDCl_3_): δ (ppm)=−15.02. ^13^C NMR (75 MHz, CDCl_3_): δ (ppm)=152.0, 148.9, 148.6, 144.3, 141.1, 137.8, 131.1, 129.2, 128.3, 121.2, 118.1, 115.1, 21.4, 19.9. ^19^F NMR (282 MHz, CDCl_3_): δ (ppm)=−136.51 (6F), −147.31 (6F). MS (APCI+): *m/z*=1297.22 [M−H]^+^, 687.22 [Cor]^+^. HRLSI‐MS (APCI+): *m/*z Calcd for [(C_67_H_34_BCuF_12_N_10_O)H]: 1297.2198; Found: 1297.2223. UV‐Vis (Toluene): *λ*_max_ (nm) (log *ϵ* (dm^3^ mol^−1^ cm^−1^))=619 (3.67), 573 (5.03), 554 (sh), 530 (4.58), 514 (sh), 417 (4.98), 399 (4.91).

**Subphthalocyanie‐corrole dyad 11**: Axial activation temperature and reaction time: 25 °C, 10 min. Axial substitution temperature: 60 °C. Toluene/heptane 2 : 1 as eluent. Yield: 27 %, dark magenta solid. ^1^H NMR (300 MHz, CDCl_3_): δ (ppm)=9.45 (dd, ^4^ *J*_H‐P_=3.0 Hz, ^3^ *J*_H‐H_=4.5 Hz, 2H), 8.98 (t, ^3^ *J*_H‐H/H‐P_=4.4 Hz, 2H), 8.91 (t, ^3^ *J*_H‐H/H‐P_=4.4 Hz, 2H), 8.73 (s, 6H), 8.62 (m, 2H), 7.59 (d, ^3^ *J*_H‐H_=8.4 Hz, 2H), 5.85 (d, ^3^ *J*_H‐H_=8.4 Hz, 2H), 3.29 (m, 12H), 1.88 (m, 12H), 1.59 (m, 12H), 1.30 (m, 48H), 0.85 (m, 18H). ^11^B NMR (160 MHz, CDCl_3_): δ (ppm)=−14.34. ^13^C NMR (126 MHz, CDCl_3_): δ (ppm)=153.6, 150.7, 140.9, 139.1, 134.5, 129.7, 128.4, 127.1, 124.8, 123.8, 119.7, 118.2, 115.8, 33.7, 31.8, 29.2, 28.5, 22.6, 14.1. ^19^F NMR (282 MHz, CDCl_3_): δ (ppm)=−37.76 (d, ^1^ *J*_P‐F_=809.3 Hz, 2F), −136.40 (4F), −152.00 (2F), −161.23 (4F). ^31^P NMR (122 MHz, CDCl_3_): δ (ppm)=182.20 (t, ^1^ *J*_P‐F_=815.0 Hz, 1P). MS (APCI+): *m/z*=2047.5 [M]^+^, 1276.6 [SubPcOH]^+^. HRLSI‐MS (MALDI‐TOF, DCTB): Calcd for [(C_109_H_120_BF_12_N_10_OPS_6_)]: 2047.7642; Found: 2047.7661. UV‐Vis (Toluene): *λ*_max_ (nm) (log *ϵ* (dm^3^ mol^−1^ cm^−1^))=600 (5.07), 582 (4.99), 565 (sh), 536 (sh), 407 (5.54), 387 (4.91), 309 (4.83).

## Conflict of interest

The authors declare no conflict of interest.

1

## Supporting information

As a service to our authors and readers, this journal provides supporting information supplied by the authors. Such materials are peer reviewed and may be re‐organized for online delivery, but are not copy‐edited or typeset. Technical support issues arising from supporting information (other than missing files) should be addressed to the authors.

Supporting Information

## Data Availability

The data that support the findings of this study are available in the supplementary material of this article.
