# Supplementary material for: Controlling Electronic Events Through Rational Structural Design in Subphthalocyanine–Corrole Dyads: Synthesis, Characterization, and Photophysical Properties
Source: Chemistry. 2022 Sep 1;28(60):e202201552. doi: 10.1002/chem.202201552 (PMC9804354; doi:10.1002/chem.202201552)
Supplement: Supplementary file 1 — Supporting Information [file CHEM-28-0-s001.pdf]

Chemistry–A European Journal

Supporting Information

Controlling Electronic Events Through Rational Structural Design in Subphthalocyanine–Corrole Dyads: Synthesis, Characterization, and Photophysical Properties

Víctor Mariñas, Benedikt Platzer, Jorge Labella, Fabrizio Caroleo, Sara Nardis,*
Roberto Paolesse,* Dirk M. Guldi,* and Tomás Torres.*

Table of content

1. Instrumentation, materials and general methods S0
2. Synthetic procedures and compound data S2
3. Mass spectra S9
4. Purity analysis by HPLC of dyads 7-11 .... S18
5. NMR characterization S21
6. Electrochemistry and Spectroelectrochemistry S34
7. Steady-state absorption and Fluorescence spectroscopy S35
8. Femtosecond Transient Absorption spectroscopy S36

1. Instrumentation, materials and general methods

All chemical reagents were purchased from Merck, TCI Europe N.V., Alfa Aesar Acros Organics or Fluka Chemie and used without further purification. Solvents were purchased from Carlo Erba Reagents, Scharlab, Carl Roth and Sigma Aldrich. "Synthetic grade" solvents were used for chemical reactions and column chromatography purifications and "anhydric grade" for reactions under dry conditions. Additionally, some solvents were further dried by distillation with Na/benzophenone (THF), or with previously activated molecular sieves (3 or 4 Å), or with a solvent purifying system by Innovative Technology Inc. HPLC grade solvents were employed for the spectroscopic characterization. Dipyrromethanes **1a-b**,[1] phthalonitrile **4c**,[2] and subphthalocyanines (SubPc) **5a-c**, [2,3] have been previously reported and were prepared as described in the literature. Corroles (Cor) **2a-b**,[4] **3b**,[5,6] were prepared following methodologies described in the literature.

The monitoring of the reactions was carried out by TLC, employing aluminum sheets coated with silica gel type 60 F_{254} (0.2 mm thick, E. Merck). The analysis of the TLCs was carried out with a UV lamp of 254 and 365 nm. Purification and separation of the synthesized products was performed by column chromatography, using silica gel (230-400 mesh, 0.040-0.063 mm, Merck). Size exclusion chromatography was performed using Bio-Beads S-X1 (200-400 mesh, Bio-Rad).

Nuclear magnetic resonance spectra (^{1}H-, ^{11}B-, ^{13}C-, ^{19}F-, ^{31}P-NMR) were recorded on a Bruker AC-300 (300 MHz), a Bruker Advance III-HD Nanobay (300 MHz) and a Bruker DRX-500 (500 MHz) spectrometers either in the Organic Chemistry Department or in the Servicio Interdepartamental de Investigación (SIdI, Interdepartmental Investigation Service) at the Universidad Autónoma de Madrid. Deuterated solvent employed in each case is indicated in brackets, and its residual peak was used to calibrate the spectra using literature reference δ ppm values.[7]

Mass spectra (MS) and high-resolution mass spectra (HR-MS) were recorded in Servicio Interdepartamental de Investigación (SIdI, Interdepartmental Investigation Service) at the Universidad Autónoma de Madrid, employing Atmospheric-Pressure Chemical Ionization (APcI) or Matrix-Assisted Laser Desorption/Ionization Time-Of-Flight (MALDI-TOF), using a MAXIS-II Bruker spectrometer for APcI, and a Bruker Ultraflex-III spectrometer with a Nd:YAG laser operating at 355 nm, for MALDI-TOF. The different matrixes employed are indicated for each spectrum.

Differential pulse and cyclic voltammetry were performed on a three-electrode setup (WE: C, CE: Pt, RE: Ag) with Fc/Fc^{+} as an internal standard. Solvents were de-aerated using Ar. For the

spectroelectrochemistry, a three electrode-setup (WE: Pt-mesh, CE: Pt, RE: Ag) was used together with a custom-made glass cell. Absorption was measured through the transparent WE with an Agilent CARY 5000 UV-Vis-NIR spectrophotometer. Solvents were de-aerated using Ar.

For steady-state absorption & fluorescence, 10x10 mm quartz cuvettes were used. Absorption was measured at a Perkin Elmer Lambda 2 spectrometer, a Horiba Jobin Yvon Fluoromax 3 was used for Fluorescence experiments. Transient absorption spectra were obtained with a Ti:sapphire laser system CPA-2101 (Clark-MXR, Inc.) paired with an Ultrafast Inc. Helios TAPPS-transient absorption pump probe spectroscopy detection unit (pump: 1 kHz repetition, 150 fs pulse width). 2x10 mm quartz cells were used while spectra were acquired with an Ultrafast Systems HELIOS transient absorption spectrometer.

HPLC analysis were performed using an Agilent 1200 equipment with a semi-preparative Daicel Chiralpak IC column (10 mm ø x 20 mmL) coupled with a UV-Vis detector. The conditions employed are indicated for each compound.

2. Synthetic Procedures and Compound Data

Synthesis and characterization of corroles **2a-b**

General method for the synthesis of trans-A_2B-Corroles:

To a 250 mL round-bottom flask equipped with a magnetic stirrer, 4-hydroxybenzaldehyde (0.50 mmol) and dipyrromethane (1.00 mmol) were dissolved in MeOH (50 mL). Once upon homogenization, a HCl solution (52.5 mL, 0.55 M) was added dropwise and stirred at 25 °C for 2 hours. The mixture was extracted with $CHCl_3$ and the organic phase was washed with H_2O (2x50 mL), brine (1x50 mL) and dried over Na_2SO_4. After filtration and solvent removal by vacuum distillation, $CHCl_3$ (250 mL) and *p*-chloranil (1.20 mmol) were added, and the solution stirred at 25 °C for 16 hours. Hydrazine (0.1 mL) was added, and solvent was removed by vacuum distillation. The resulting residue was purified by column chromatography on silica gel using a suitable eluent, as specified in each case.

10-(4-hydroxyphenyl)-5,15-bis(1,3,5-trimethylphenyl)corrole **2a**

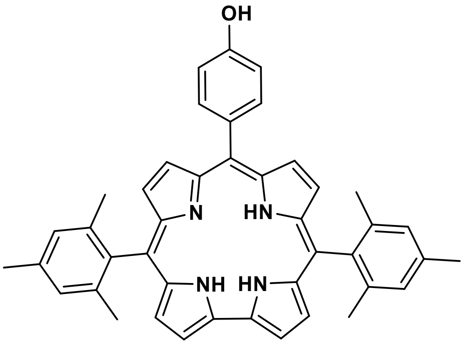

Cor **2a** was prepared following the general method described above and purified by column chromatography on silica gel using DCM as eluent. The product obtained from the column was further purified by precipitation with DCM/Hexane. **2a** was isolated as a purplish-green solid in 37% yield. **^{1}H-NMR** (400 MHz, $CDCl_3$): δ(ppm) = 8.87 (d, $^3J_{H\text{-}H}$= 4.4 Hz, 2H), 8.48 (m, 4H), 8.31 (d, $^3J_{H\text{-}H}$ = 4.0 Hz, 2H), 8.01 (d, $^3J_{H\text{-}H}$ = 8.4 Hz, 2H), 7.18 (d, $^3J_{H\text{-}H}$ = 8.4 Hz, 2H), 2.60 (s, 6H), 1.91 (s, 12H). **^{13}C-NMR** (126 MHz, $CDCl_3$): δ(ppm) = 143.3, 142.7, 140.9, 140.1, 139.3, 135.6, 135.1, 131.6, 130.0, 128.3, 128.1, 126.7, 126.3, 125.6, 120.6, 118.9, 115.1, 114.2, 22.7, 21.5. **MS** (MALDI-TOF, DCTB): *m/z* = 625.4 $[M-H]^+$, 626.4 $[M]^+$. **HRLSI-MS** (APcI+): *m/z* Calcd for $[(C_{43}H_{38}N_4O)H]$: 627.3118; Found: 627.3126 $[M+H]^+$ + 626.3057 $[M]^+$. **UV-Vis** (Toluene): λ_{max} (nm) (log ε (dm^3 mol^{-1} cm^{-1})) = 641 (3.6), 607 (3.8), 566 (4.1), 428 (4.8), 410 (4.7), 410 (4.9).

5,15-bis(pentafluorophenyl)-10-(4-hydroxyphenyl)corrole **2b**

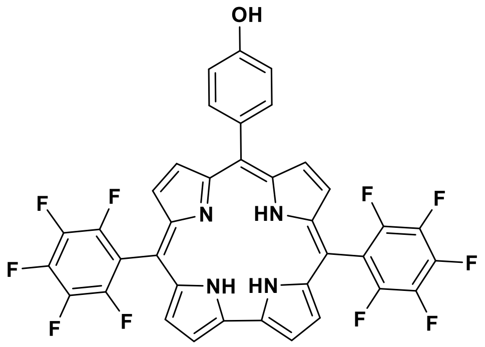

Cor **2b** was prepared following the general method described above and purified by column chromatography on silica gel using DCM as eluent. **2b** was isolated as a red solid in 14% yield. **^{1}H-NMR** (400 MHz, $CDCl_3$): δ (ppm) = 9.12 (d, $^3J_{H-H}$ = 4.4 Hz, 2H), 8.71 (m, 4H), 8.57 (d, $^3J_{H-H}$ = 4.0 Hz, 2H), 8.05 (d, $^3J_{H-H}$ = 8.4 Hz, 2H), 7.22 (d, $^3J_{H-H}$ = 8.4 Hz, 2H). **MS** (ESI+): *m/z* = 723.6 $[M+H]^+$. **UV/Vis** ($CHCl_3$): λ_{max} (nm) (log ε (dm^3 mol^{-1} cm^{-1}) = 639 (3.4), 613 (3.6), 560 (4.0), 412 (4.8).

Synthesis and characterization of corroles **3a-b**

10-(4-hydroxyphenyl)-5,15-bis(1,3,5-trimethylphenyl)corrolato-Cu **3a**

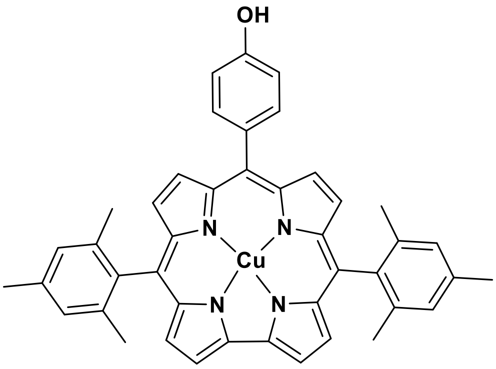

To a 50 mL round-bottom flask equipped with a magnetic stirrer, Cor **2a** (0.07 mmol) was added over $CHCl_3$ (10 mL) and stirred at 40 °C for 5 minutes. $Cu(AcO)_2$ (0.55 mmol) dissolved in hot MeOH (5 mL) was added and the solution was further stirred at 40 °C for 15 minutes. The solvent was removed by vacuum distillation and the resulting solid was subjected to column chromatography on silica gel using a mixture of DCM/Heptane 5:1 as eluent. The product obtained from the column was further purified by precipitation with DCM/Hexane. **3a** was isolated as a brown solid in 85% yield. **^{1}H-NMR** (300 MHz, $CDCl_3$): δ (ppm) = 7.96 (d, $^3J_{H-H}$ = 8.4 Hz, 2H), 7.14 (d, $^3J_{H-H}$ = 8.4 Hz, 2H), 2.41 (s, 6H), 2.01 (s, 12H). **MS** (MALDI-TOF, DCTB): *m/z* = 686.3 $[M]^+$. **HRLSI-MS** (APcI+): *m/z* Calcd for $[(C_{43}H_{35}N_4OCu)H]$: 687.2180; Found: 687.2200. **UV-Vis** (Toluene): λ_{max} (nm) (log ε (dm^3 mol^{-1} cm^{-1})) = 621 (3.45), 538 (3.80), 421 (4.76), 392 (4.69).

5,15-bis(pentafluorophenyl)-10-(4-hydroxyphenyl)corrolato-P(fluorine)$_2$ **3b**

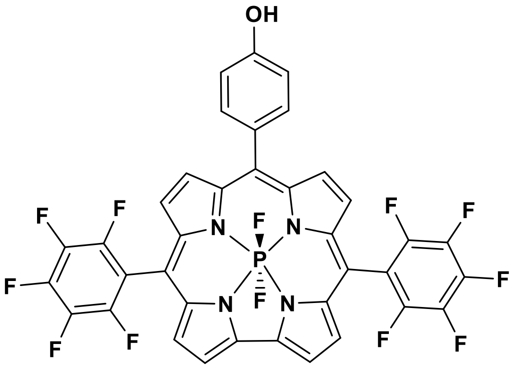

To a 50 mL round-bottom flask equipped with a magnetic stirrer, Cor **2b** (0.20 mmol) was added over pyridine (11 mL), degassed and warmed up to reflux. An excess of PCl_3 (20.00 mmol) was added and the solution was stirred at reflux for 30 minutes. Upon complete consumption of the starting material, water was added dropwise till the formation of a precipitate. The resulting solid was filtered, washed with water, recovered with $CHCl_3$ and evaporated to dryness. The mixture was dissolved in DCM (30 mL) and added to a plastic flask equipped with a magnetic stirrer. HF 50% (11 mL) was added and the mixture was stirred vigorously in dark conditions for 16h. The mixture was extracted with $CHCl_3$ and the organic phase was washed with H_2O (2x50 mL), $NaHCO_3$ (1x50 mL) and dried over Na_2SO_4. After filtration and solvent removal by vacuum distillation, the resulting solid was subjected to column chromatography on silica gel using a mixture of $CHCl_3$/MeOH 9:1 as eluent. **3b** was isolated from the column as a bright red solid in 73% yield. **^{1}H-NMR** (400 MHz, $CDCl_3$) δ (ppm): 9.46 (dd, $^4J_{H\text{-}P}$ = 3.2 Hz, $^3J_{H\text{-}H}$ = 4.4 Hz, 2H), 9.06 (t, $^3J_{H\text{-}H/H\text{-}P}$ = 4.4 Hz, 2H), 9.01 (m, 4H), 8.07 (d, $^3J_{H\text{-}H}$ = 8.4 Hz, 2H), 7.25 (d, J = 8.4 Hz, 2H). **^{19}F-NMR** (376 MHz, $CDCl_3$) δ (ppm): -37.5 (d, $^1J_{P\text{-}F}$ = 810 Hz 2F,), -136.3 (m, 4F), -151.7 (m, 2F), -161.1 (m, 4F**).** **^{31}P-NMR** (162 MHz, $CDCl_3$) δ (ppm): -182.1 (t, $^1J_{P\text{-}F}$ = 810 Hz, 1P). **HRLSI-MS** (APcI): *m/z* Calcd for [($C_{37}H_{13}F_{12}N_4OP$)]: 788.0635; Found: 788.0635. **UV-Vis** ($CHCl_3$): λ_{max} (nm) (log ε (dm^3 mol^{-1} cm^{-1})) = 582 (4.62), 565 (4.51), 536 (sh), 407 (5.58), 386 (4.83).

Synthesis and characterization of SubPc-Cor dyads **7-11**

General method for the synthesis of SubPc-Cor dyads:

To a 10 mL Schlenk flask equipped with a magnetic stirrer, dry toluene (3 mL) was added to a mixture of the corresponding SubPc (0.12 mmol) and AgOTf (0.14 mmol), under argon atmosphere. The mixture was stirred at 25-60 ^{o}C for 2-6 hours (10 min for SubPc **5c**). The corresponding Cor (0.12 mmol) and DIPEA (0.144 mmol for SubPc-Cor dyads **7**, **8**, **9** and **11**; 0.174 mmol for SubPc-Cor dyad **10**) were then added and the suspension stirred at 40-80 ^{o}C for 24 hours (18 hours for SubPc-Cor dyad **11**). The solvent was removed by vacuum distillation and the resulting solid was subjected to column chromatography on silica gel using a suitable eluent, as specified in each case. Each compound was further purified by size exclusion chromatography in $CHCl_3$.

SubPc-Cor dyad **7**

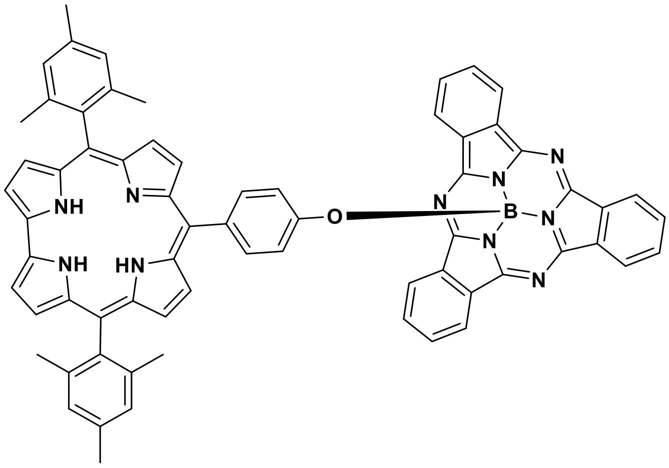

SubPc-Cor **7** was prepared following the general method described above. Axial activation temperature and reaction time: 40 ^{o}C, 2h. Axial substitution temperature and reaction time: 40 ^{o}C, 24h. The compound was purified by column chromatography on silica gel using toluene/THF 20:1 (v/v) as eluent, and the residue was further purified by size exclusion chromatography in $CHCl_3$. SubPc-Cor **7** was isolated as a dark purple solid in 30% yield. **^{1}H-NMR** (300 MHz, $CDCl_3$): δ (ppm) = 8.95 (m, 6H), 8.85 (d, $^{3}J_{H\text{-}H}$= 4.2 Hz, 2H), 8.42 (d, $^{3}J_{H\text{-}H}$ = 4.5 Hz, 2H), 8.29 (d, $^{3}J_{H\text{-}H}$ = 4.2 Hz, 2H), 8.25 (d, $^{3}J_{H\text{-}H}$ = 4.8 Hz, 2H), 7.96 (m, 6H), 7.54 (d, $^{3}J_{H\text{-}H}$ = 8.1 Hz, 2H), 5.75 (d, $^{3}J_{H\text{-}H}$ = 8.1 Hz, 2H), 2.61 (s, 6H), 1.91 (s, 12H). **^{11}B-NMR** (96 MHz, $CDCl_3$): δ (ppm) = -14.53. **^{13}C-NMR** (75 MHz, $CDCl_3$): δ (ppm) = 152.2, 151.5, 139.3, 137.6, 135.7, 135.0, 134.8, 131.1, 131.0, 129.9, 129.9, 128.0, 126.3, 122.3, 122.2, 117.7, 115.0, 109.6, 22.7, 21.1. **MS** (APCI+): *m/z* = 1021.43 $[M]^{+}$, 627.31 $[Cor+H]^{+}$. **HRLSI-MS:** (APCI+): *m/z* Calcd for $[(C_{67}H_{49}BN_{10}O)H]$: 1021.4267; Found: 1021.4291. **UV-Vis** (Toluene): λ_{max} (nm) (log ε (dm^{3} mol^{-1} cm^{-1})) = 640 (3.87), 606 (3.99), 563 (4.95), 544 (sh), 518 (4.49), 427 (4.84), 410 (4.92), 303 (4.67).

SubPc-Cor dyad **8**

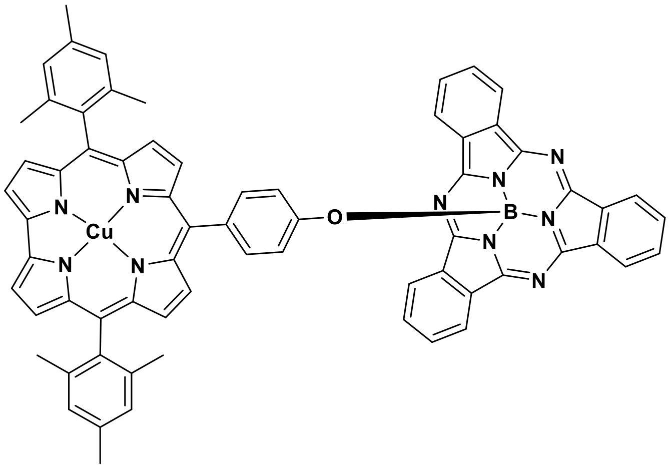

SubPc-Cor **8** was prepared following the general method described above. Axial activation temperature and reaction time: 40 ^{o}C, 2h. Axial substitution temperature and reaction time: 40 ^{o}C, 24h. The compound was purified by column chromatography on silica gel using toluene/THF 20:1 (v/v) as eluent, and the residue was further purified by size exclusion chromatography in $CHCl_3$.

SubPc-Cor **8** was isolated as a brown solid in 32% yield. **^{1}H-NMR** (300 MHz, $CDCl_3$): δ (ppm) = 8.87 (m, 6H), 7.91 (m, 6H), 7.31 (d, $^{3}J_{H\text{-}H}$ = 8.4 Hz, 2H), 5.49 (d, $^{3}J_{H\text{-}H}$ = 8.4 Hz, 2H), 2.40 (s, 6H), 2.03 (s, 12H). **^{11}B-NMR** (96 MHz, $CDCl_3$): δ (ppm) = -14.73. **^{13}C-NMR** (75 MHz, $CDCl_3$): δ (ppm) = 153.4, 151.4, 137.5, 131.0, 129.9, 129.0, 128.9, 128.2, 128.1, 122.3, 120.9, 118.3, 21.2, 19.8. **MS** (MALDI-TOF, DCTB): *m/z* = 1080.3 $[M]^+$. **HRLSI-MS** (APCI+): *m/z* Calcd for $[(C_{67}H_{46}BCuN_{10}O)H]$: 1081.3329; Found: 1081.3339. **UV-Vis** (Toluene): λ_{max} (nm) (log ε (dm^3 mol^{-1} cm^{-1})) = 622 (3.52), 5.63 (4.87), 545 (4.61), 516 (4.41), 416 (4.76), 3.99 (4.71), 300 (4.64).

<u>SubPc-Cor dyad **9**</u>

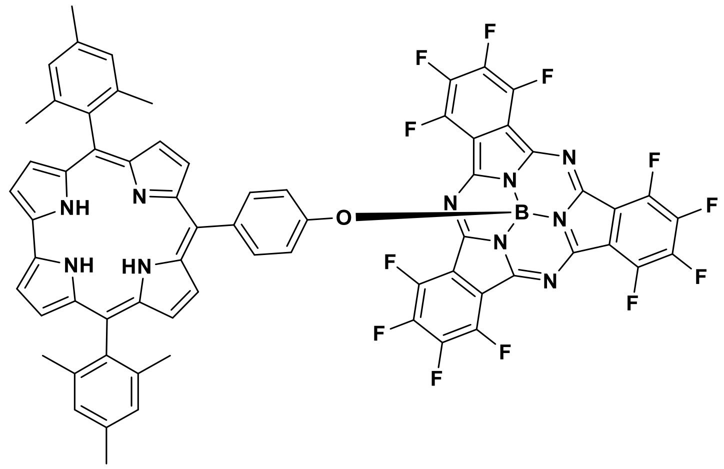

SubPc-Cor **9** was prepared following the general method described above. Axial activation temperature and reaction time: 60 ^{o}C, 6h. Axial substitution temperature and reaction time: 80 ^{o}C, 24h. The compound was purified by column chromatography on silica gel using toluene/heptane 2:1 (v/v) as eluent, and the residue was further purified by size exclusion chromatography in $CHCl_3$. SubPc-Cor **9** was isolated as a dark magenta solid in 15% yield. **^{1}H-NMR** (300 MHz, $CDCl_3$): δ (ppm) = 8.86 (d, $^{3}J_{H\text{-}H}$ = 4.2 Hz, 2H), 8.44 (d, $^{3}J_{H\text{-}H}$ = 4.8 Hz, 2H), 8.30 (d, *J* = 4.2 Hz, 2H), 8.21 (d, $^{3}J_{H\text{-}H}$ = 4.8 Hz, 2H), 7.59 (d, $^{3}J_{H\text{-}H}$ = 8.4 Hz, 2H), 5.69 (d, $^{3}J_{H\text{-}H}$ = 8.4 Hz, 2H), 2.60 (s, 6H), 1.89 (s, 12H). **^{11}B-NMR** (160 MHz, $CDCl_3$): δ (ppm) = -14.74. **^{13}C-NMR** (126 MHz, $CDCl_3$): δ (ppm) = 152.2, 152.0, 144.3 (m), 141.1 (m), 139.3, 137.6, 135.7, 135.0, 134.8, 131.2, 131.0, 129.9, 126.4, 122.3, 117.7, 115.1 (m), 115.0, 109.6, 22.7, 21.1. **^{19}F-NMR** (471 MHz, $CDCl_3$): δ (ppm) = -136.50 (6F); -147.24 (6F). **MS** (MALDI-TOF, DCTB): *m/z* = 1235.3 $[M\text{-}H]^+$, 1236.3 $[M]^+$, 625.4 $[Cor\text{-}H]^+$, 626.4 $[Cor]^+$. **HRLSI-MS** (APCI+): *m/z* Calcd for $[(C_{67}H_{37}BF_{12}N_{10}O)H]$: 1237.3137; Found: 1237.3143. **UV-Vis** (Toluene): λ_{max} (nm) (log *e* (dm^3 mol^{-1} cm^{-1})) = 638 (3.28), 606 (3.67), 573 (4.80), 553 (sh), 528 (3.32), 428 (4.79), 408 (4.87), 394 (sh).

SubPc-Cor dyad **10**

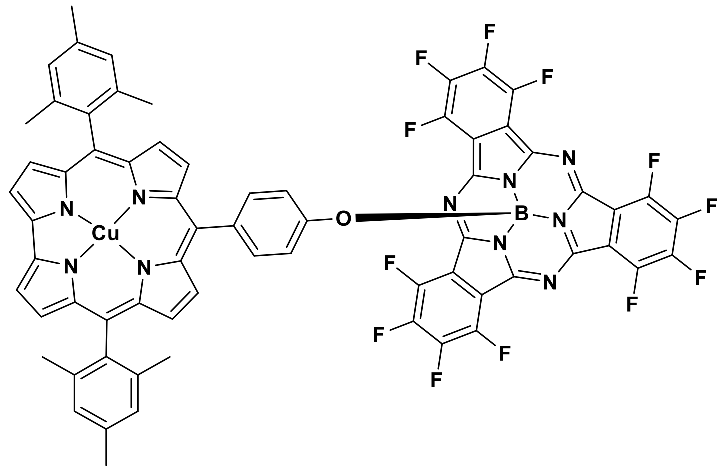

SubPc-Cor **10** was prepared following the general method described above. Axial activation temperature and reaction time: 60 ^{o}C, 6h. Axial substitution temperature and reaction time: 65 ^{o}C, 24h. The compound was purified by column chromatography on silica gel using toluene/heptane 2:1 (v/v) as eluent, and the residue was further purified by size exclusion chromatography in $CHCl_3$. SubPc-Cor **10** was isolated as a dark magenta solid in 20% yield. **^{1}H-NMR** (300 MHz, $CDCl_3$): δ (ppm) = 7.33 (d, $^{3}J_{H\text{-}H}$ = 8.4 Hz, 2H), 5.50 (d, $^{3}J_{H\text{-}H}$ = 8.4 Hz, 2H), 2.41 (s, 6H), 2.04 (s, 12H). **^{11}B-NMR** (96 MHz, $CDCl_3$): δ (ppm) = -15.02. **^{13}C-NMR** (75 MHz, $CDCl_3$): δ (ppm) = 152.0, 148.9, 148.6, 144.3, 141.1, 137.8, 131.1, 129.2, 128.3, 121.2, 118.1, 115.1, 21.4, 19.9. **^{19}F-NMR** (282 MHz, $CDCl_3$): δ (ppm) = -136.51 (6F), -147.26 (6F). **MS** (APCI+): *m/z* = 1297.22 $[M]^+$, 687.22 $[Cor]^+$. **HRLSI-MS** (APCI+): *m/*z Calcd for $[(C_{67}H_{34}BCuF_{12}N_{10}O)H]$: 1297.2198; Found: 1297.2223. **UV-Vis** (Toluene): λ_{max} (nm) (log ε (dm^3 mol^{-1} cm^{-1})) = 619 (3.67), 573 (5.03), 554 (sh), 530 (4.58), 514 (sh), 417 (4.98), 399 (4.91).

SubPc-Cor dyad **11**

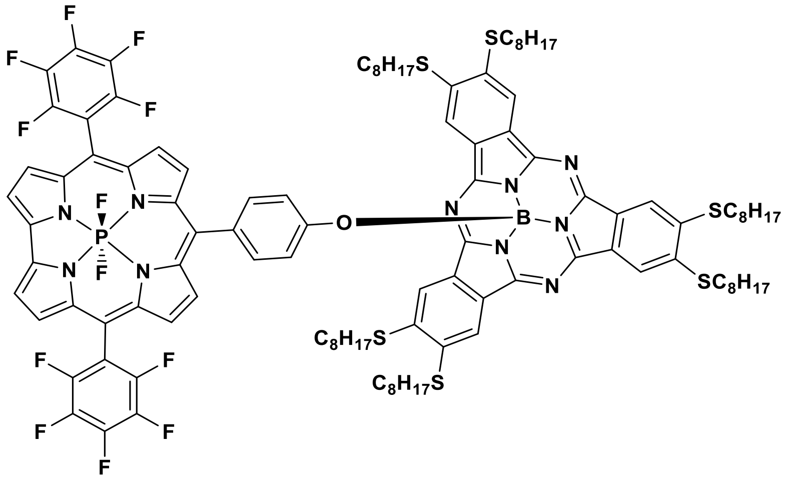

SubPc-Cor **11** was prepared following the general method described above. Axial activation temperature and reaction time: 25 °C, 10 min. Axial substitution temperature and reaction time: 60 °C, 18h. The compound was purified by column chromatography on silica gel using toluene/heptane 2:1 (v/v) as eluent, and the residue was further purified by size exclusion chromatography in $CHCl_3$. SubPc-Cor **11** was isolated as a dark magenta solid in 27% yield. **^{1}H-NMR** (300 MHz, $CDCl_3$): δ (ppm) = 9.45 (dd, $^4J_{H-P}$ = 3.0 Hz, $^3J_{H-H}$ = 4.5 Hz, 2H), 8.98 (t, $^3J_{H-H/H-P}$ = 4.4 Hz, 2H), 8.91 (t, $^3J_{H-H/H-P}$ = 4.4 Hz, 2H), 8.73 (s, 6H), 8.62 (m, 2H), 7.59 (d, $^3J_{H-H}$ = 8.4 Hz, 2H), 5.85 (d, $^3J_{H-H}$ = 8.4 Hz, 2H), 3.29 (m, 12H), 1.88 (m, 12H), 1.59 (m, 12H), 1.30 (m, 48H), 0.85 (m, 18H). **^{11}B-NMR** (160 MHz, $CDCl_3$): δ(ppm) = -14.34. **^{13}C-NMR** (126 MHz, $CDCl_3$): δ(ppm) = 153.6, 150.7, 140.9, 139.1, 134.5, 129.7, 128.4, 127.1, 124.8, 123.8, 119.7, 118.2, 115.8, 33.7, 31.8, 29.2, 28.5, 22.6, 14.1. **^{19}F-NMR** (282 MHz, $CDCl_3$): δ (ppm) = -37.76 (d, $^1J_{P-F}$ = 809.3 Hz, 2F), -136.40 (4F), -152.00 (2F), -161.23 (4F). **^{31}P-NMR** (122 MHz, $CDCl_3$): δ (ppm) = 182.20 (t, $^1J_{P-F}$ = 815.0 Hz, 1P). **MS** (MALDI-TOF, DCTB): *m/z* = 2047.7 $[M]^+$, 1276.6 $[SubPcOH]^+$. **HRLSI-MS** (MALDI-TOF, DCTB): *m/z* Calcd for $[(C_{109}H_{120}BF_{12}N_{10}OPS_6)]$: 2047.7642; Found: 2047.7661. **UV-Vis** (Toluene): λ_{max} (nm) (log ε (dm^3 mol^{-1} cm^{-1})) = 600 (5.07), 582 (4.99), 565 (sh), 536 (sh), 407 (5.54), 387 (4.91), 309 (4.83).

3. Mass Spectra

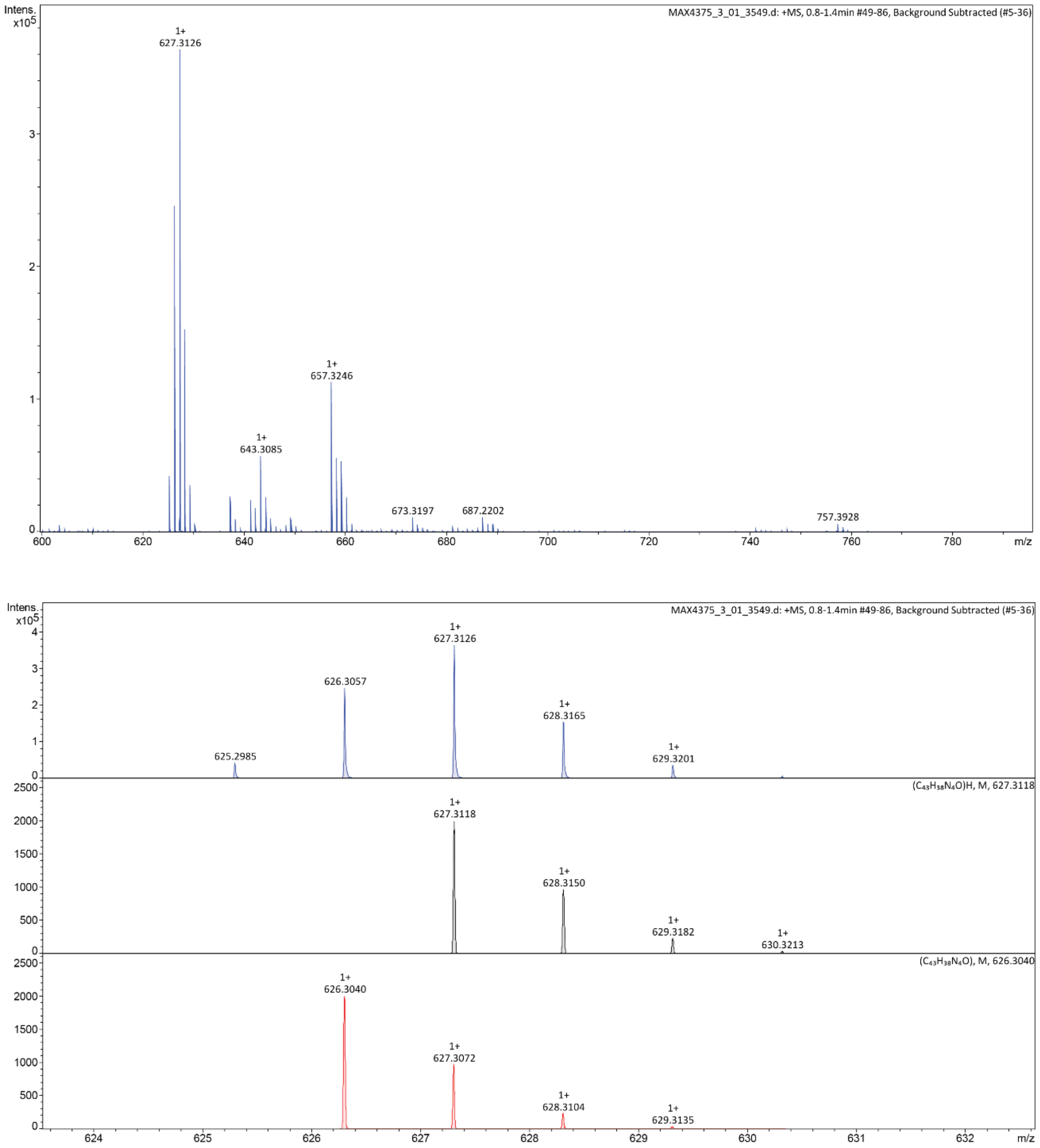

Figure S3.1. Mass spectra of **2a**.

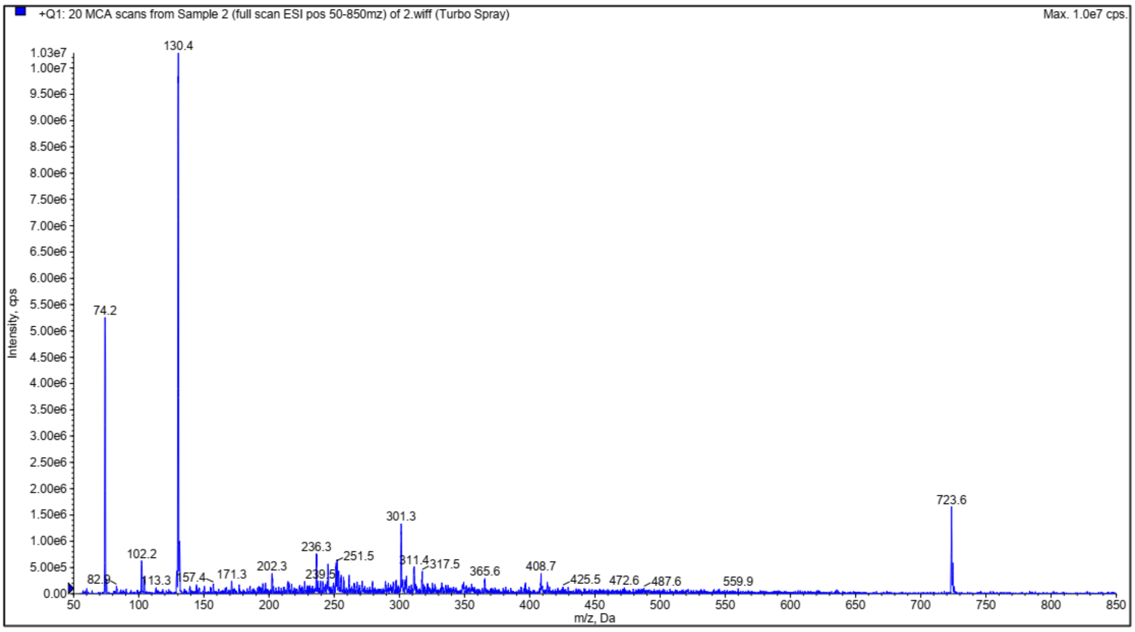

Figure S3.2. Mass spectrum of **2b**.

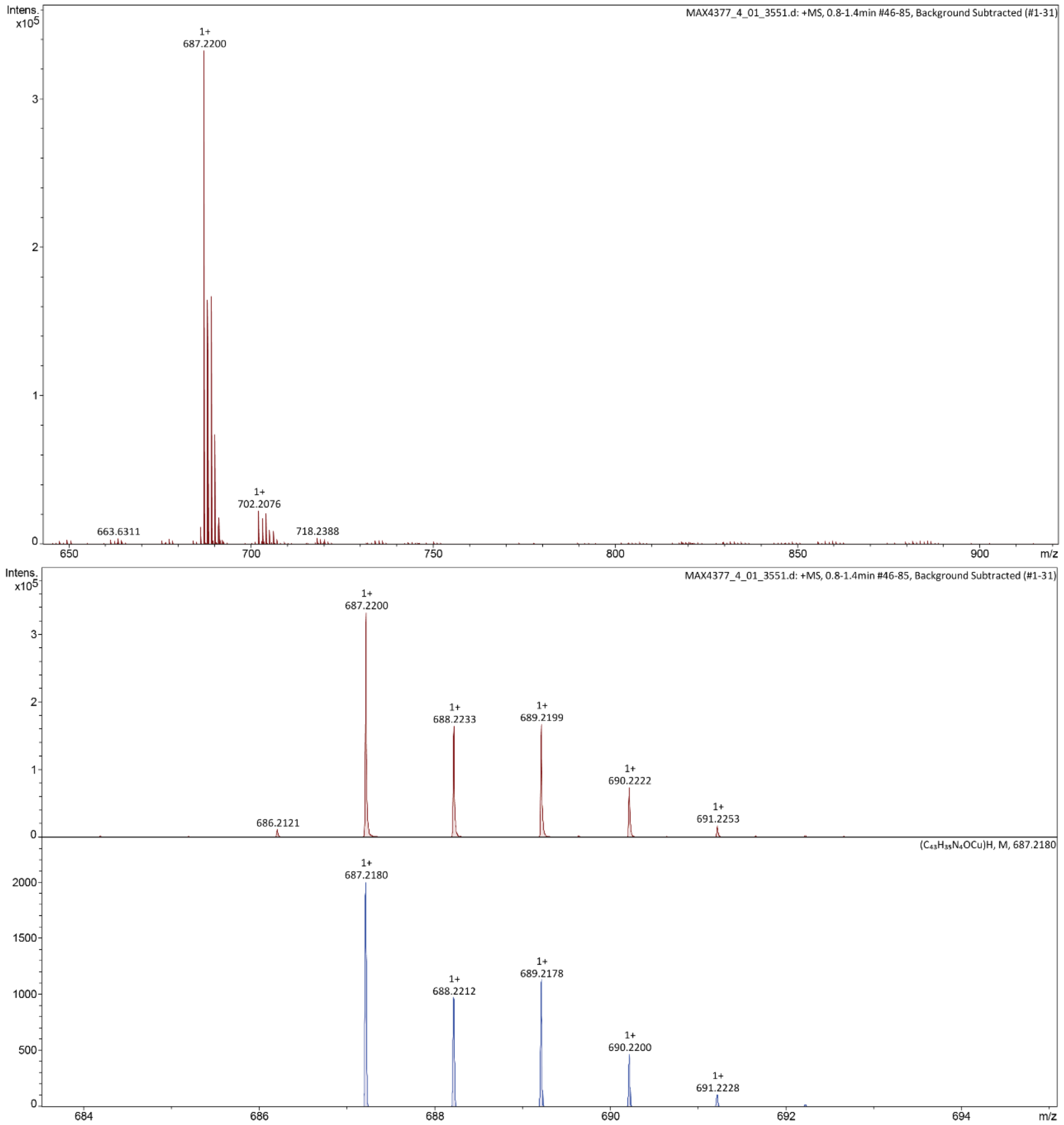

Figure S3.3. Mass spectra of **3a**.

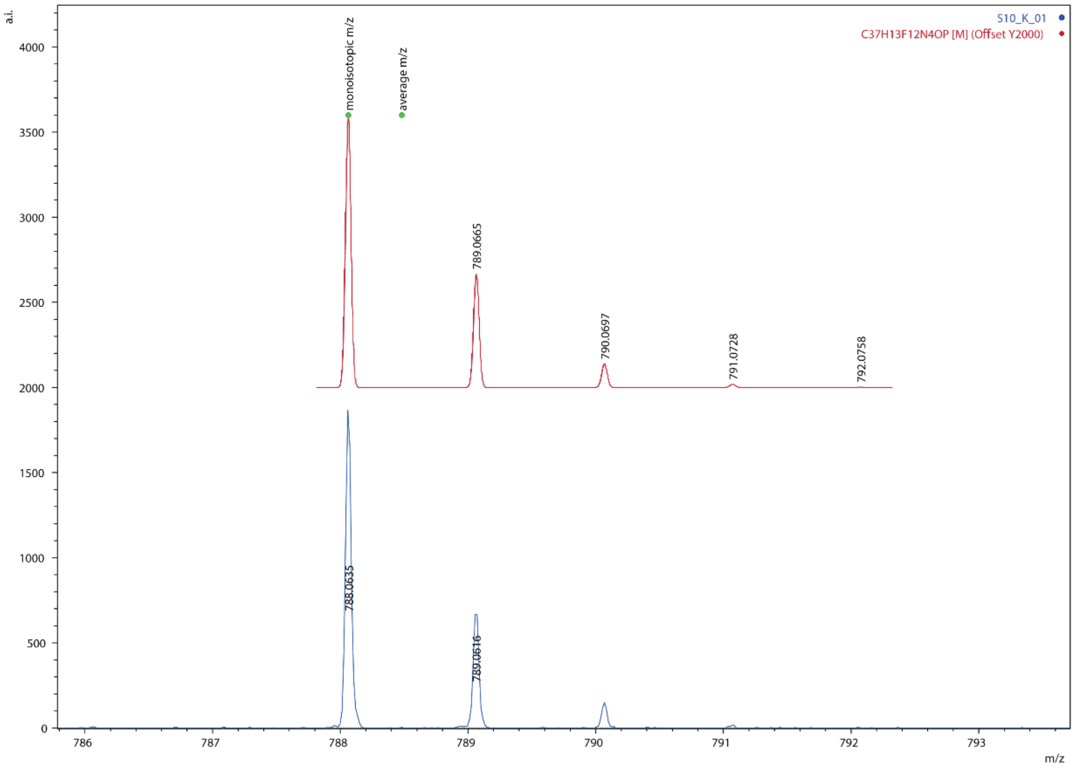

Figure S3.4. Mass spectrum of **3b**.

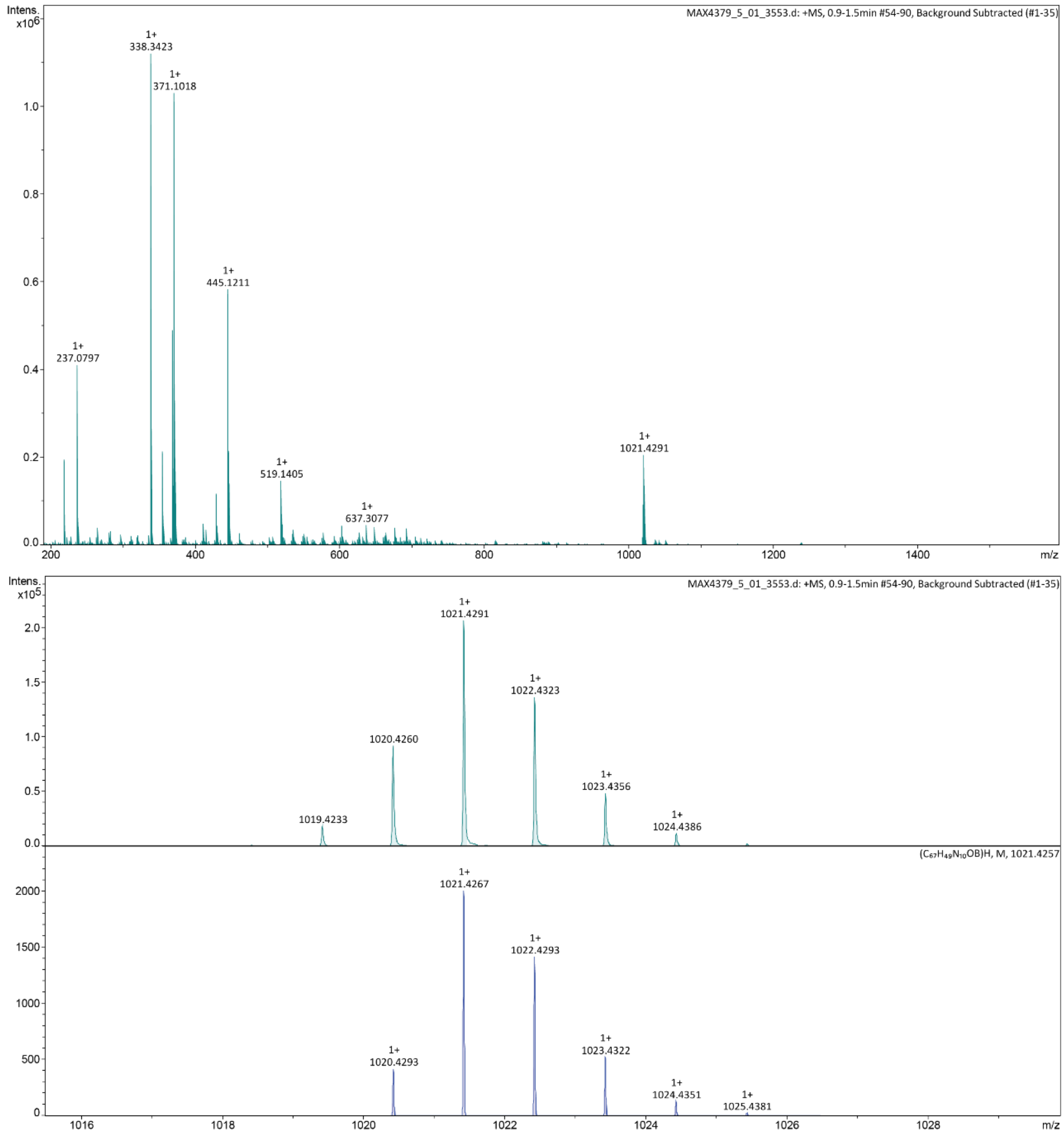

Figure S3.5. Mass spectra of **7**.

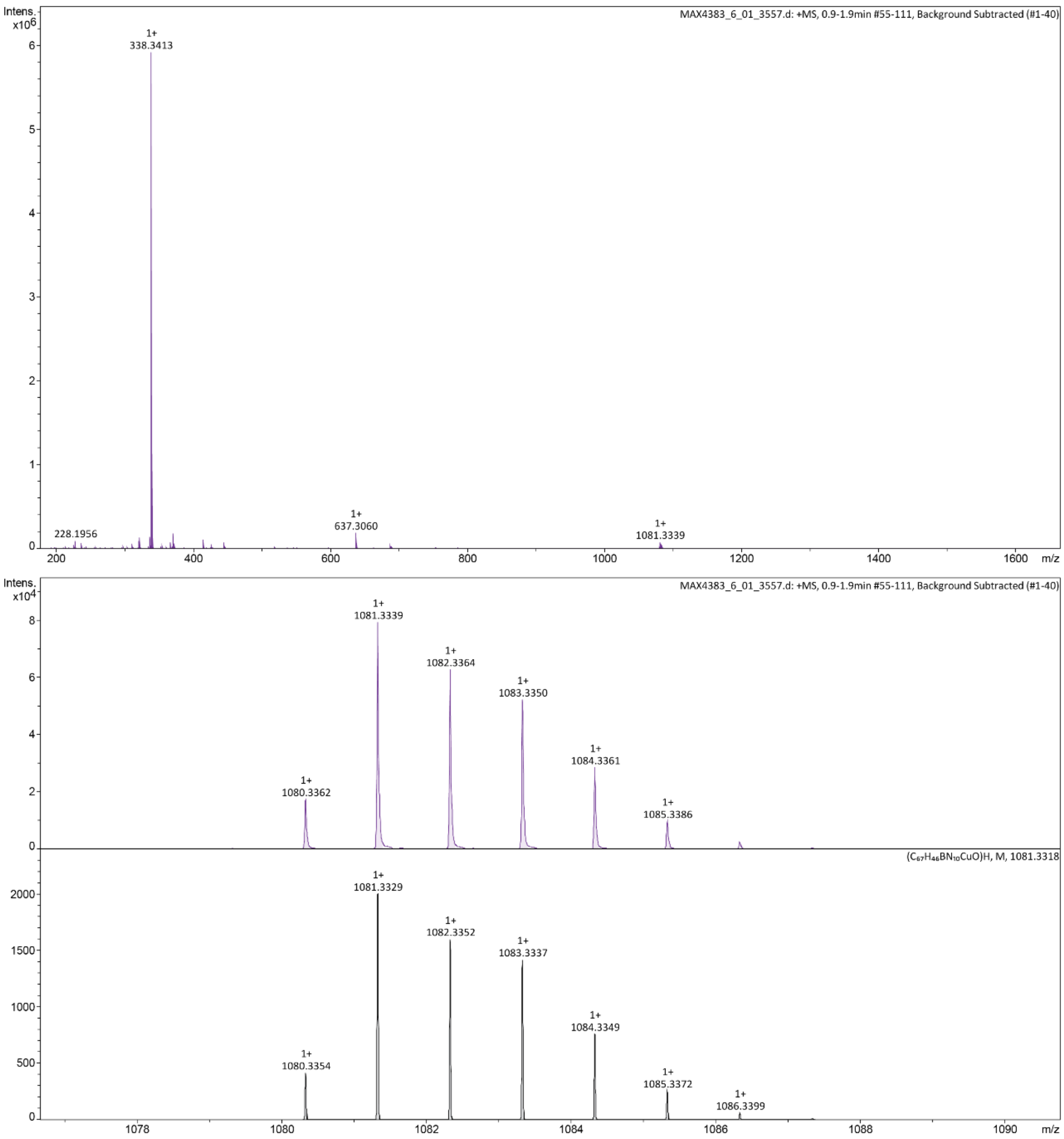

Figure S3.6. Mass spectra of **8**.

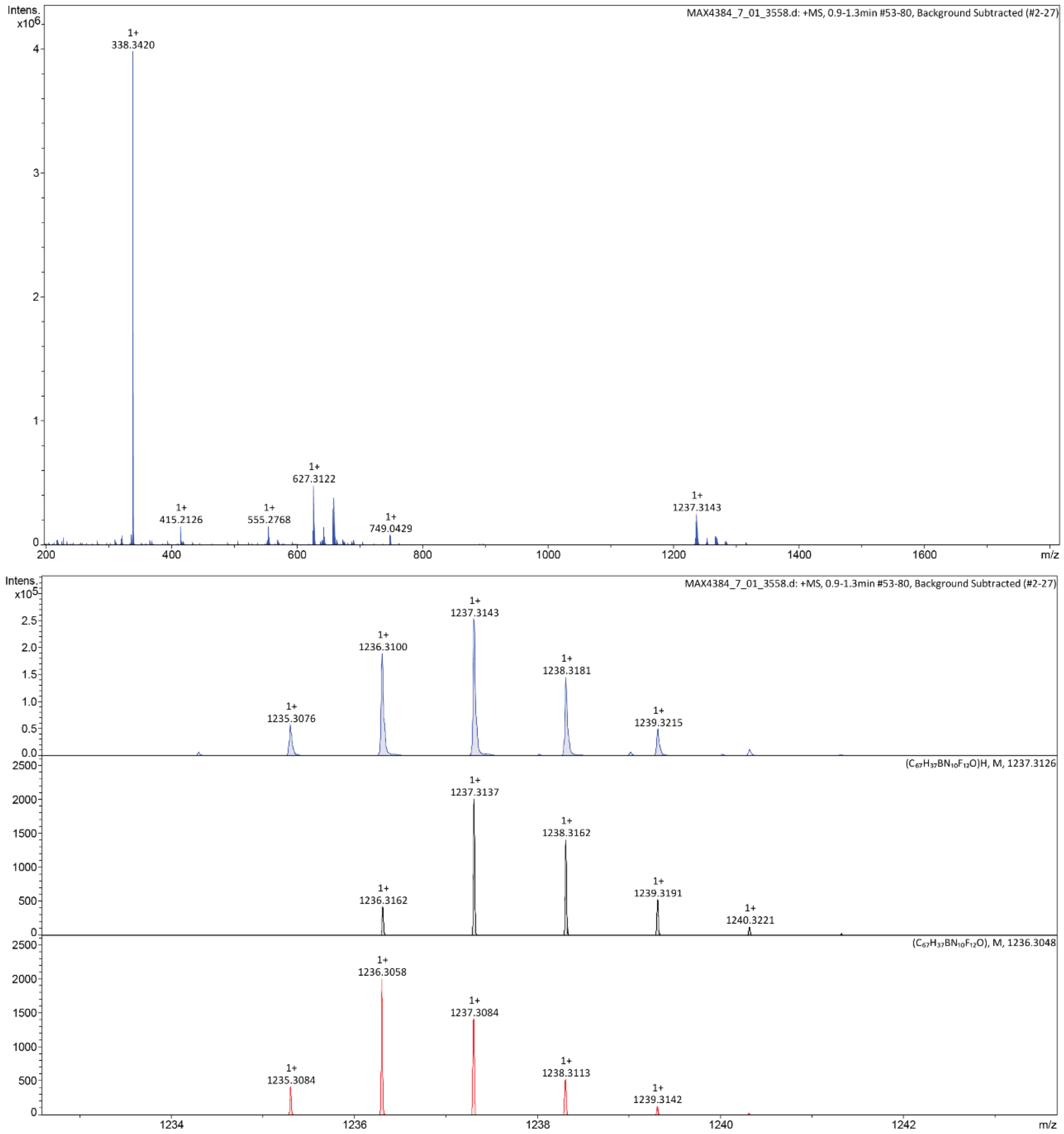

Figure S3.7. Mass spectra of **9**.

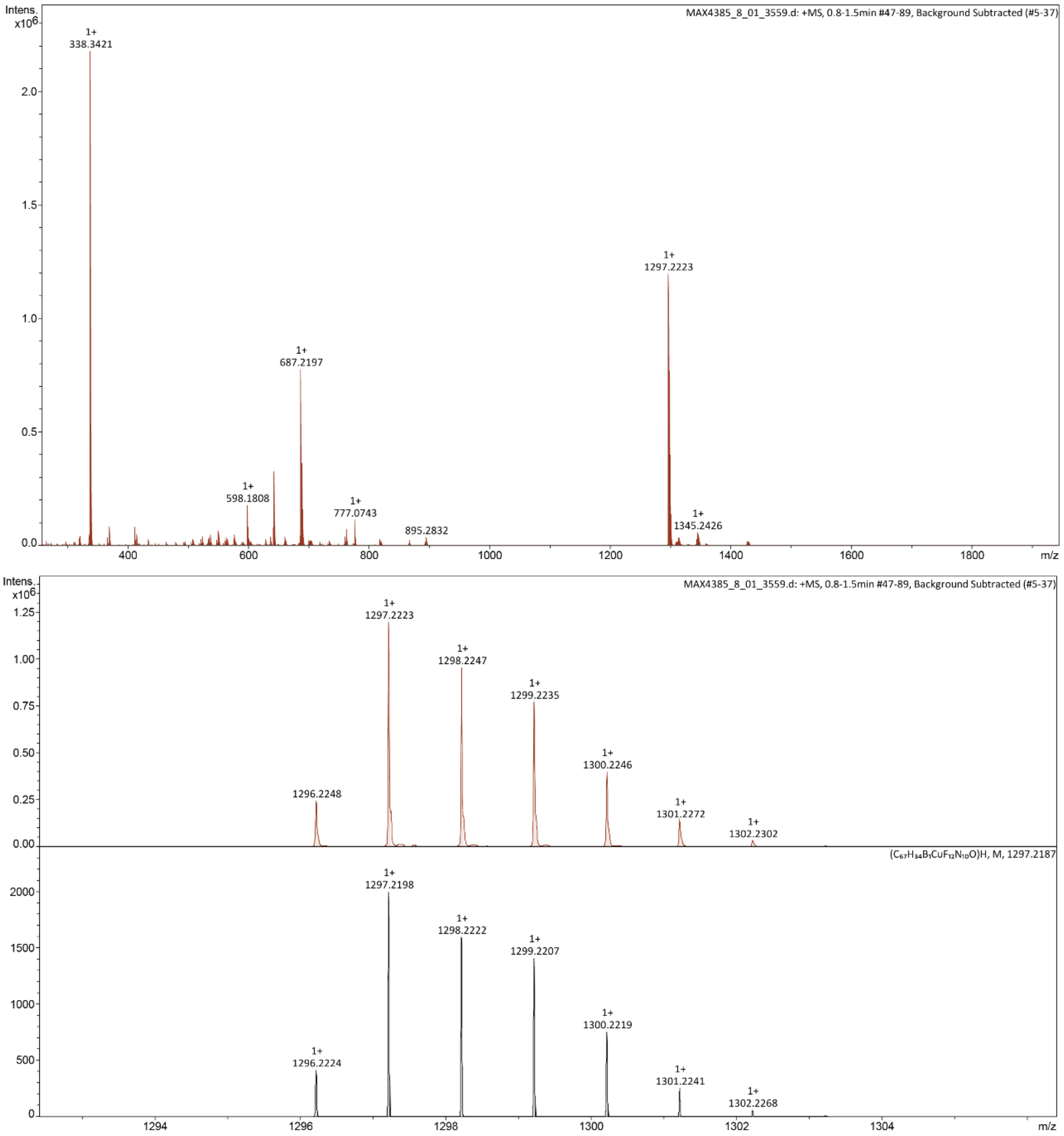

Figure S3.8. Mass spectra of **10**.

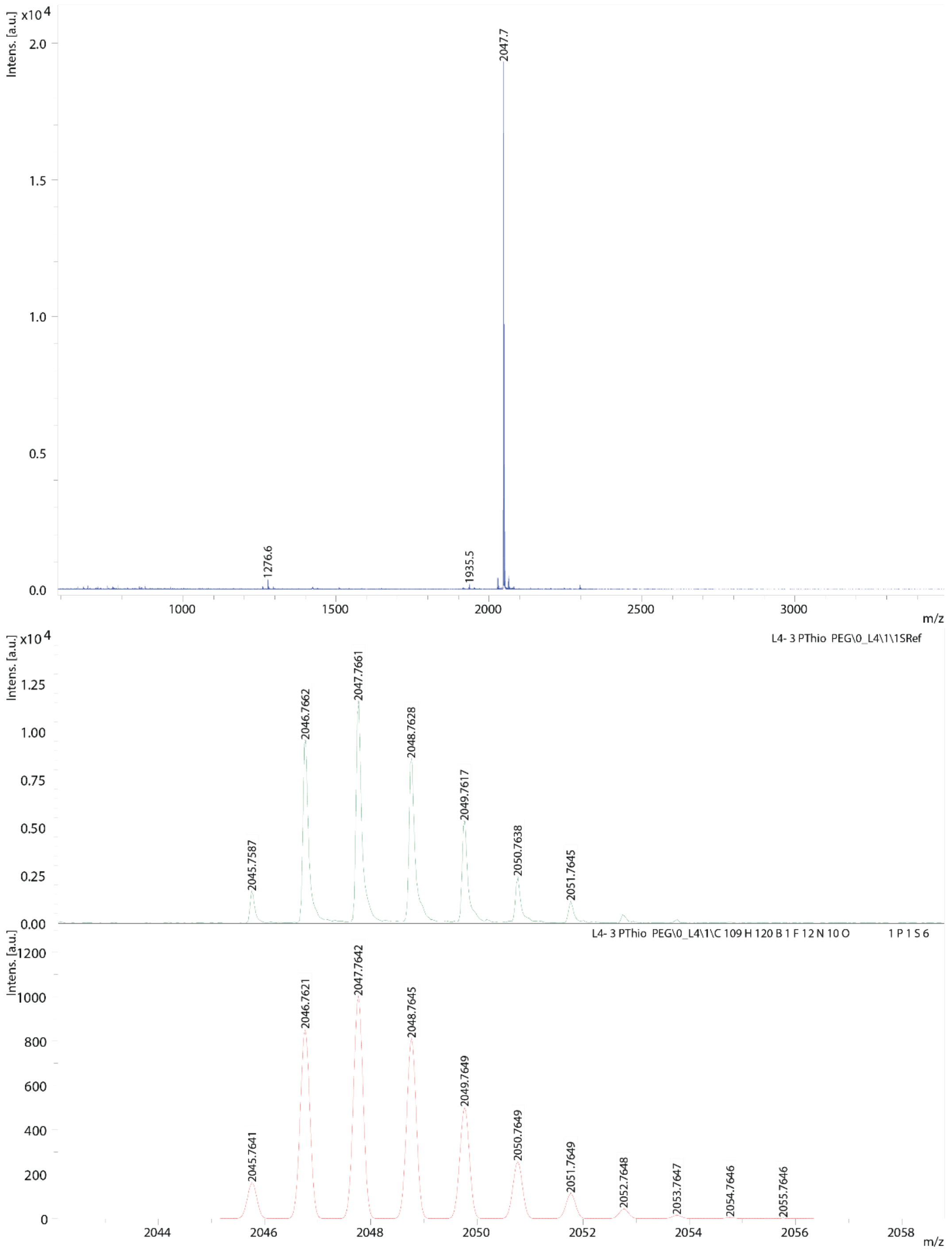

Figure S3.9. Mass spectra of **11**.

4. Purity analysis by HPLC of dyads 7-11

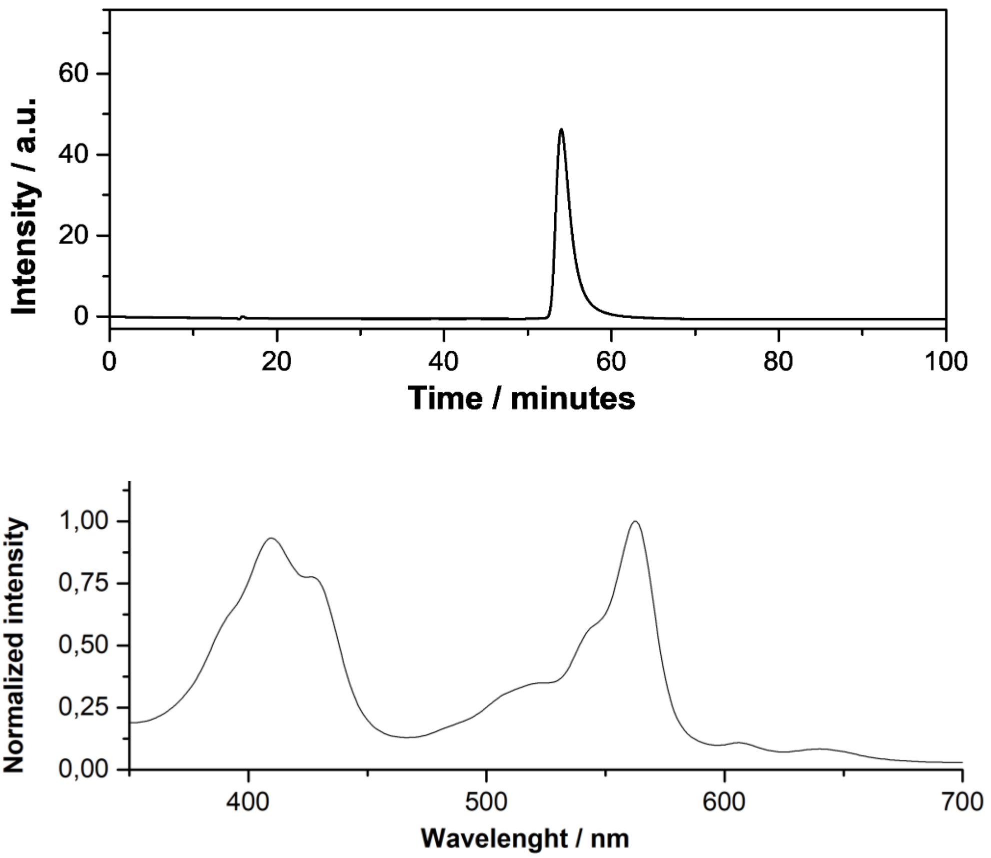

Figure S4.1. HPLC chromatogram (top) and UV-Vis spectrum (bottom) of **7**. Eluting solvents = toluene; flow rate = 1.2 mL min^{-1}; temperature = 20 ºC, detection wavelength = 400, 500 and 570 nm.

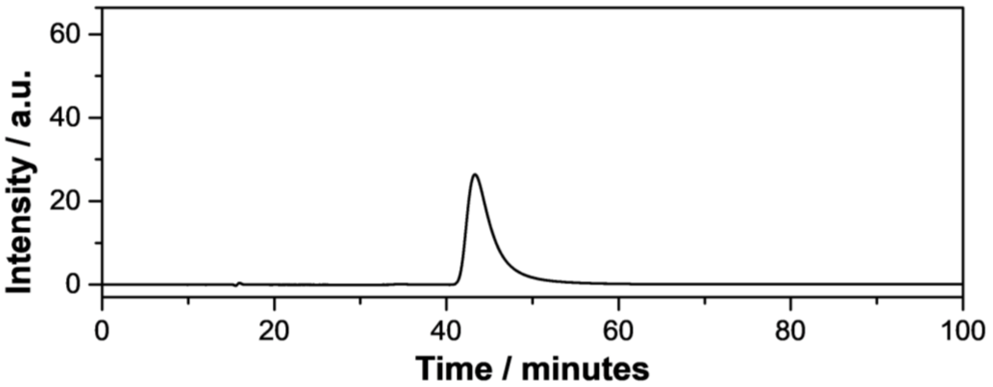

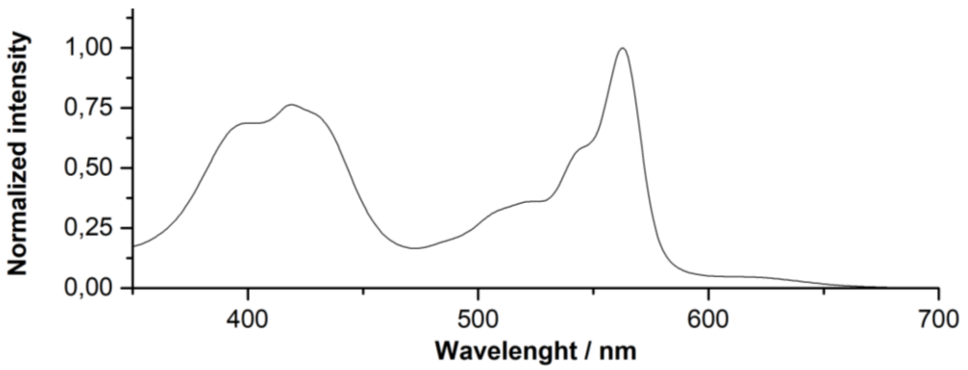

Figure S4.2. HPLC chromatogram (top) and UV-Vis spectrum (bottom) of **8**. Eluting solvents = toluene/AcOEt 50:1; flow rate = 1.0 mL min^{-1}; temperature = 20 ºC, detection wavelength = 400, 500 and 570 nm.

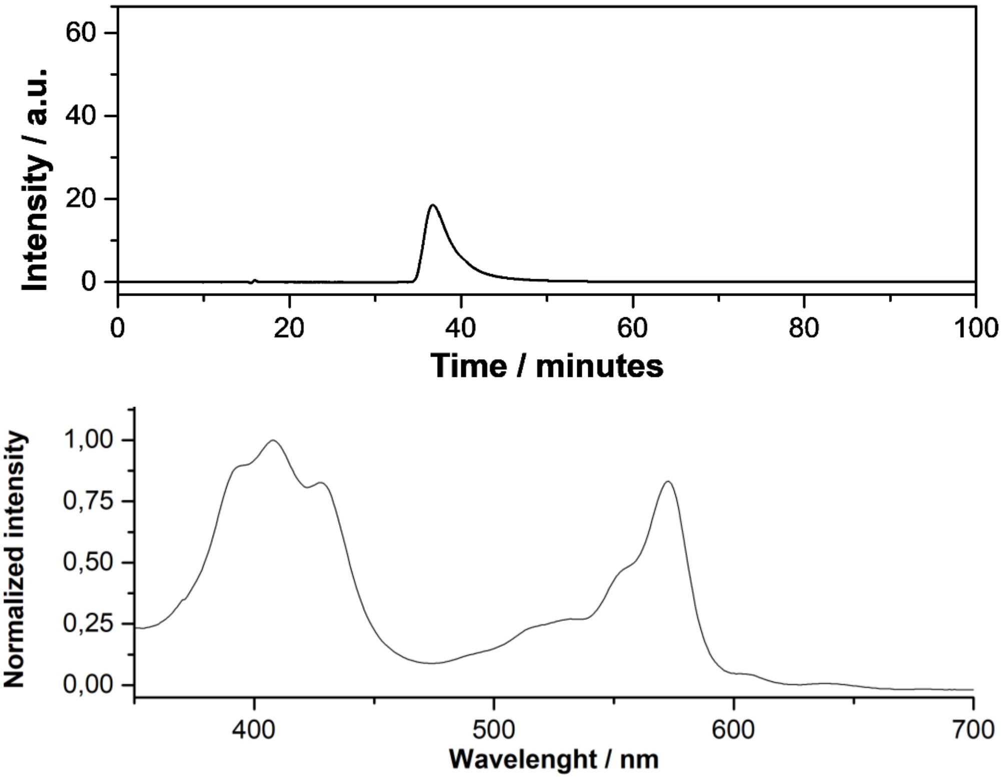

Figure S4.3. HPLC chromatogram (top) and UV-Vis spectrum (bottom) of **9**. Eluting solvents = toluene; flow rate = 1.2 mL min^{-1}; temperature = 20 ºC, detection wavelength = 400, 500 and 570 nm.

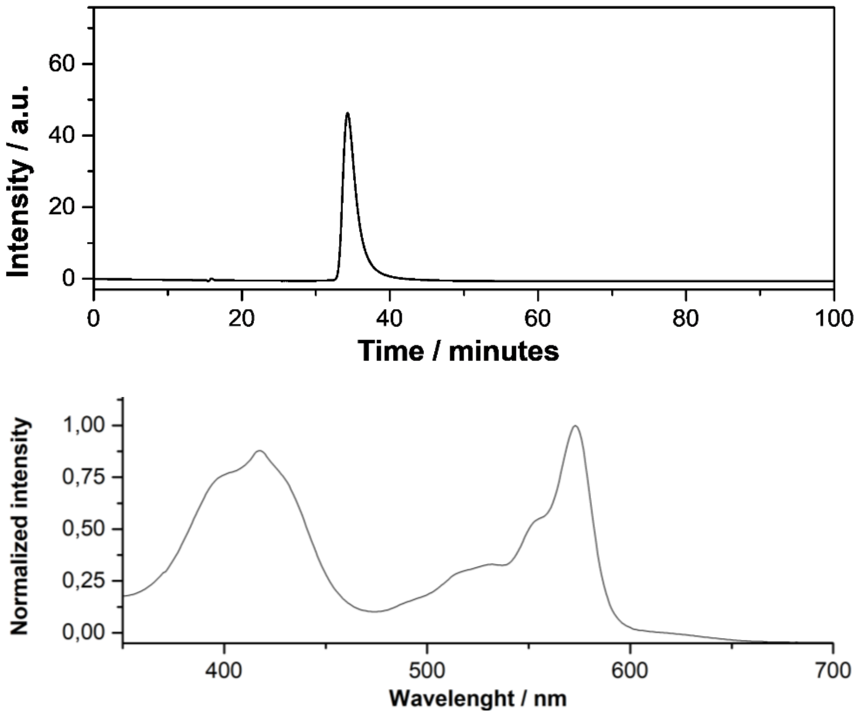

Figure S4.4. HPLC chromatogram (top) and UV-Vis spectrum (bottom) of **10**. Eluting solvents = toluene/AcOEt 50:1; flow rate = 1.2 mL min^{-1}; temperature = 20 ºC, detection wavelength = 400, 500 and 570 nm.

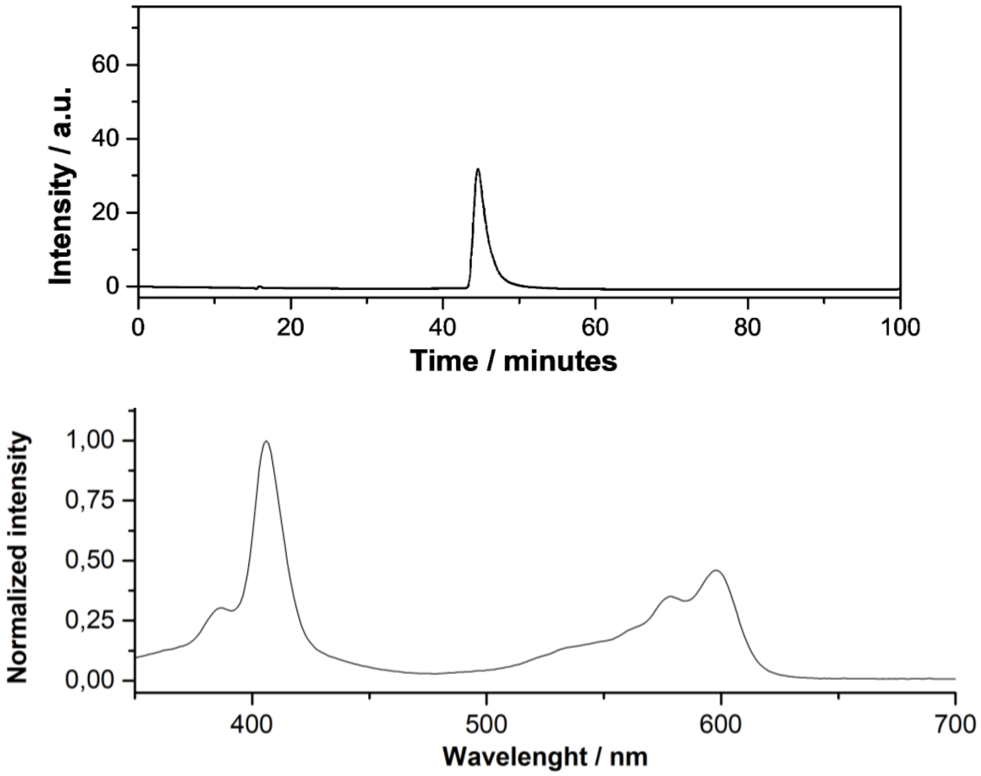

Figure S4.5. HPLC chromatogram (top) and UV-Vis spectrum (bottom) of **11**. Eluting solvents = toluene/hexane 70:30; flow rate = 1.0 mL min^{-1}; temperature = 20 ºC, detection wavelength = 400, 500 and 570 nm.

5. NMR Characterization

NMR spectra of *trans*-A_2B-Corrole precursors

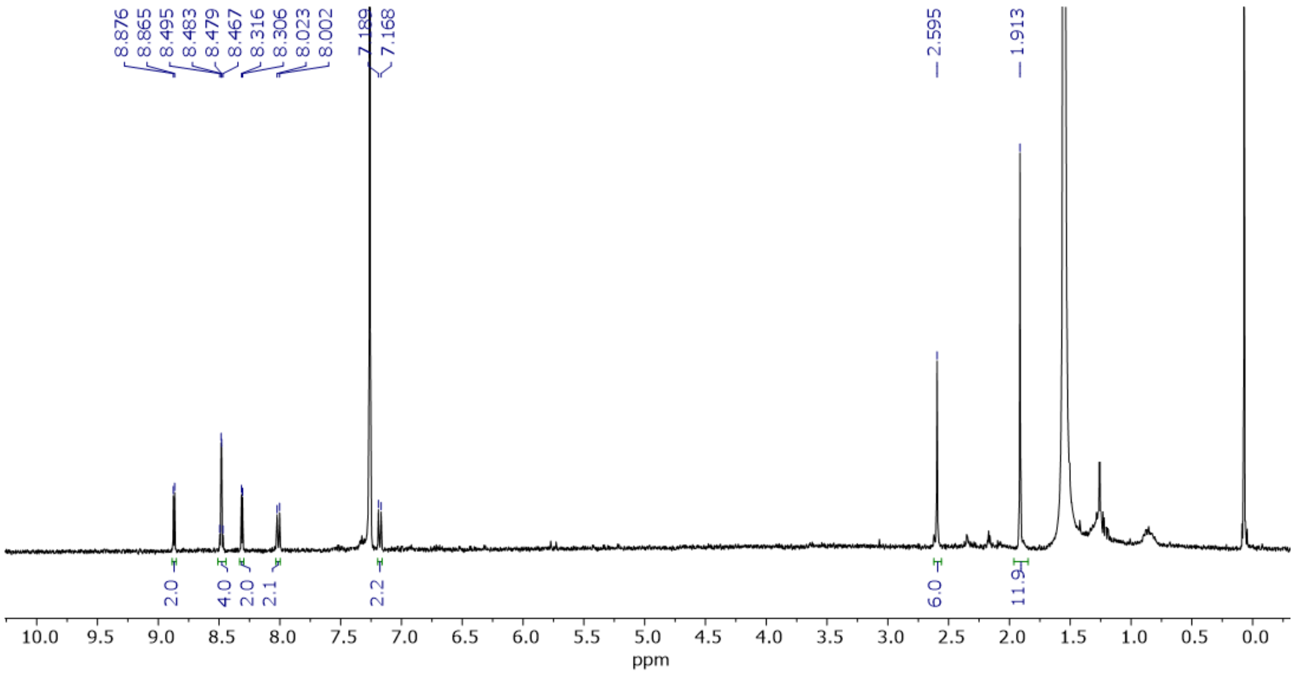

Figure S5.1. ^{1}H-NMR spectrum of ($CDCl_3$) **2a**.

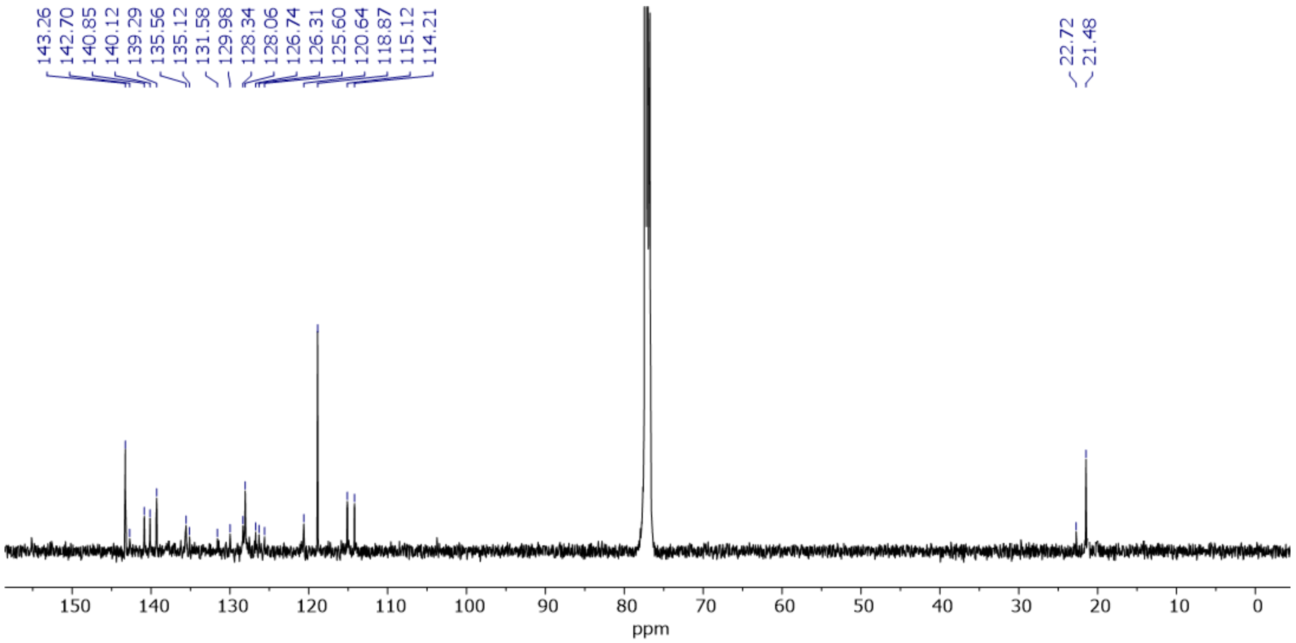

Figure S5.2. ^{13}C-NMR spectrum of ($CDCl_3$) **2a**.

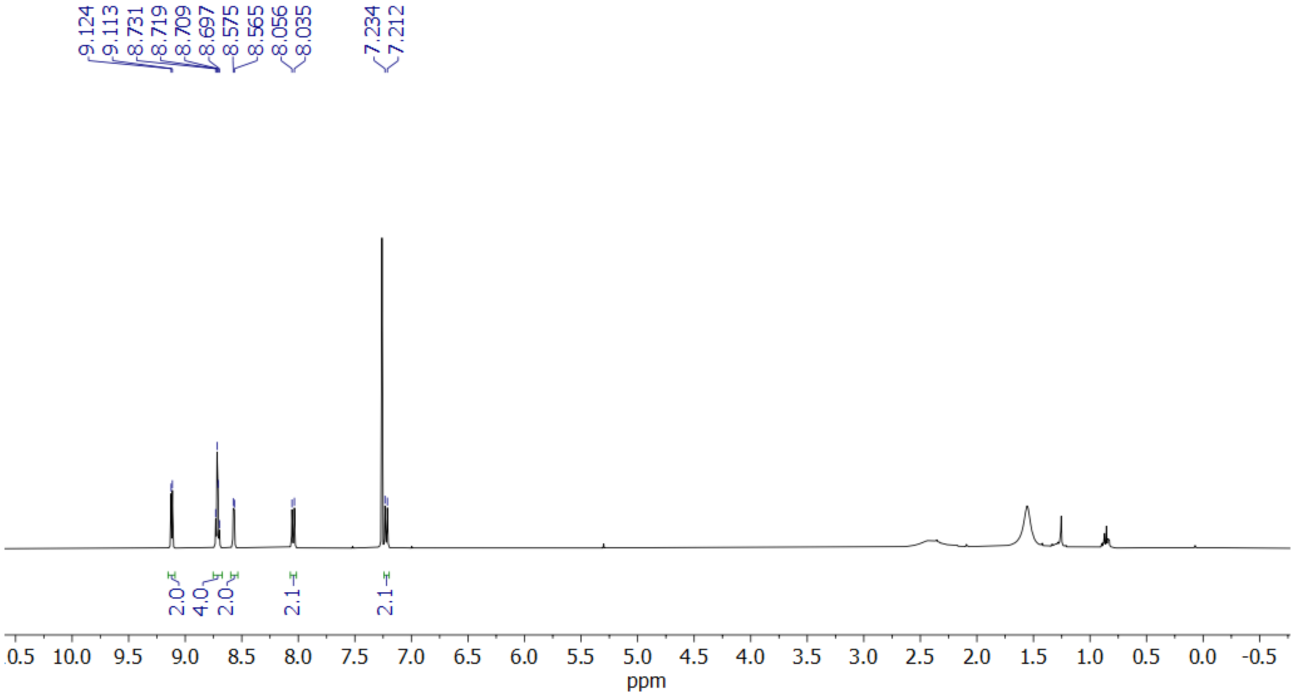

Figure S5.3. ^{1}H-NMR spectrum of ($CDCl_3$) **2b**.

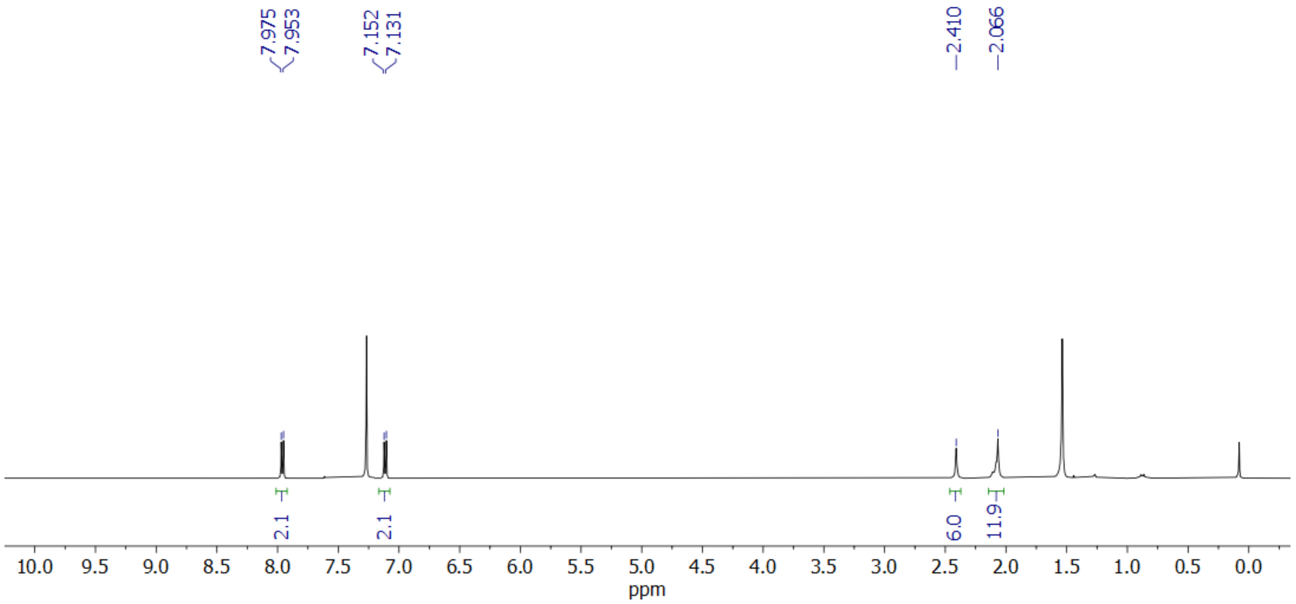

Figure S5.4. ^{1}H-NMR spectrum of ($CDCl_3$) **3a**.

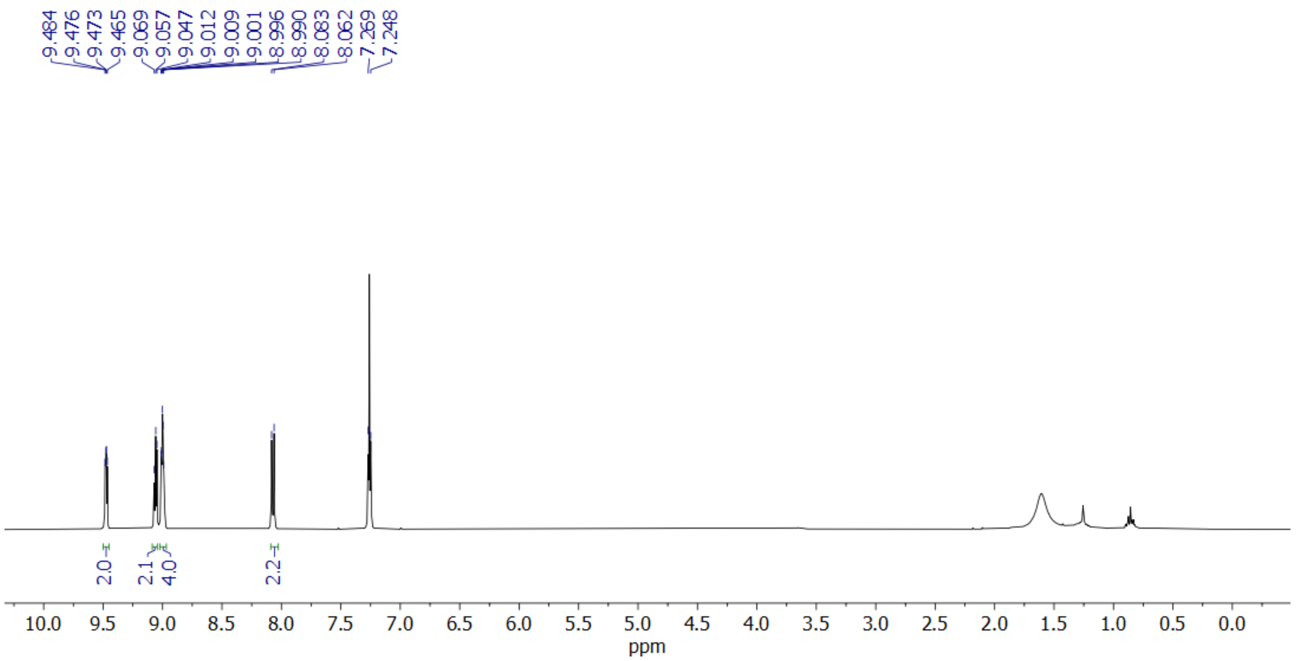

Figure S5.5. ^{1}H-NMR spectrum of (CDCl$_3$) **3b**.

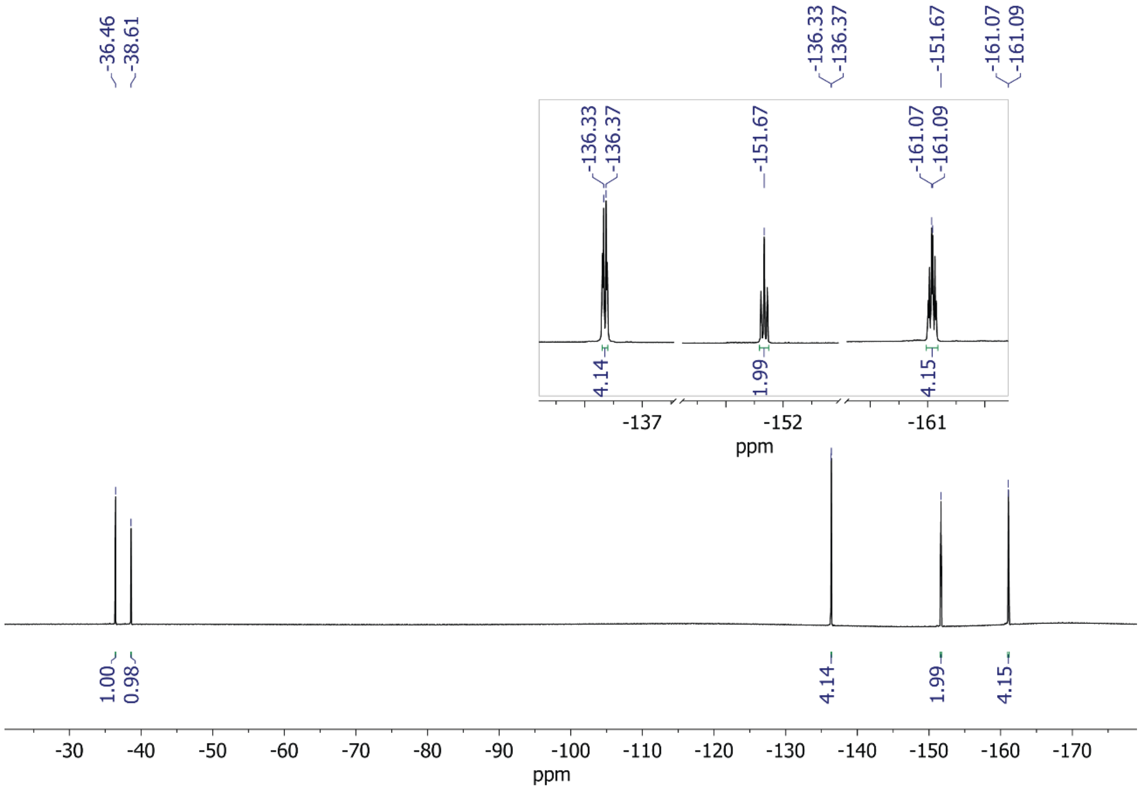

Figure S5.6. ^{19}F-NMR spectrum of (CDCl$_3$) **3b**.

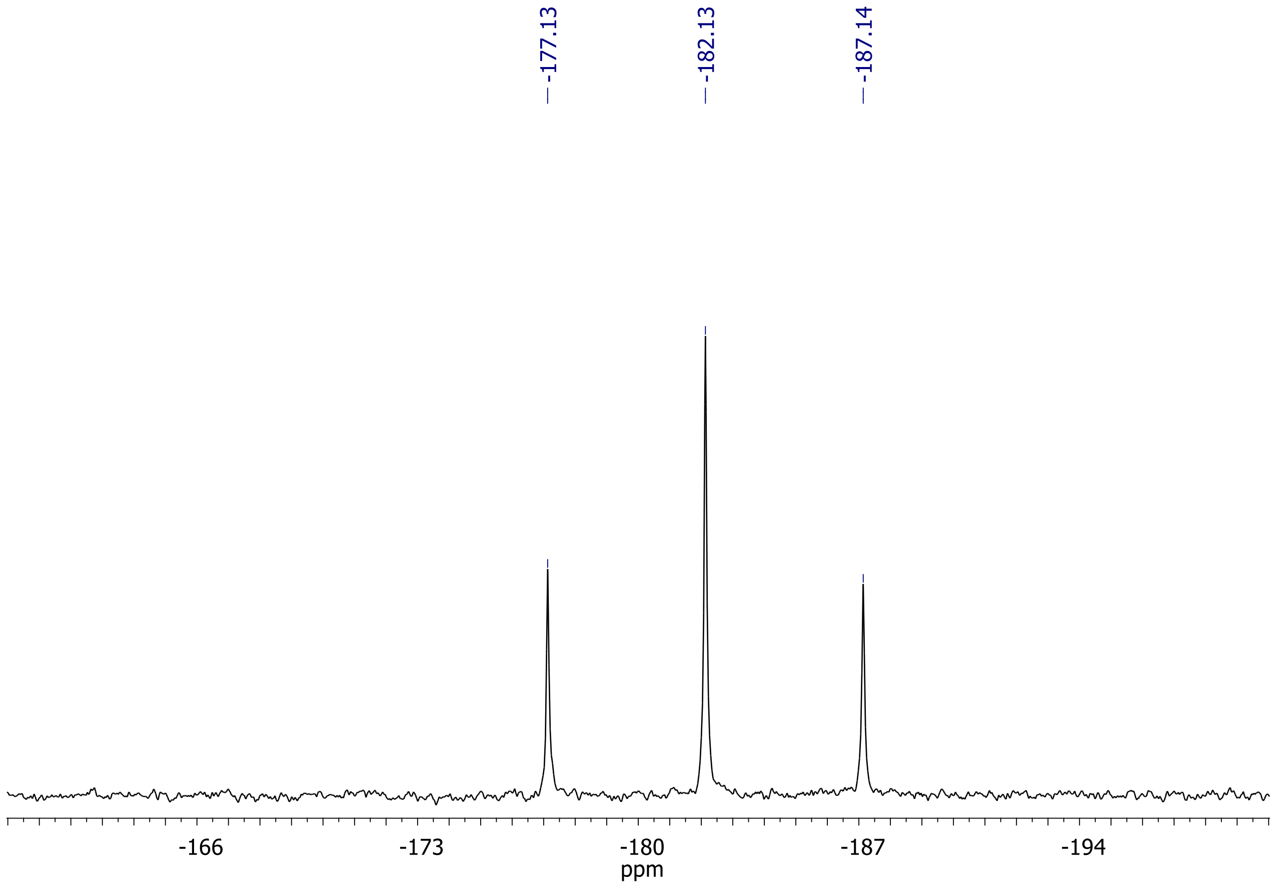

Figure S5.7. ^{31}P-NMR spectrum of ($CDCl_3$) **3b**.

NMR spectra of SubPc-Corrole dyads **7-11**:

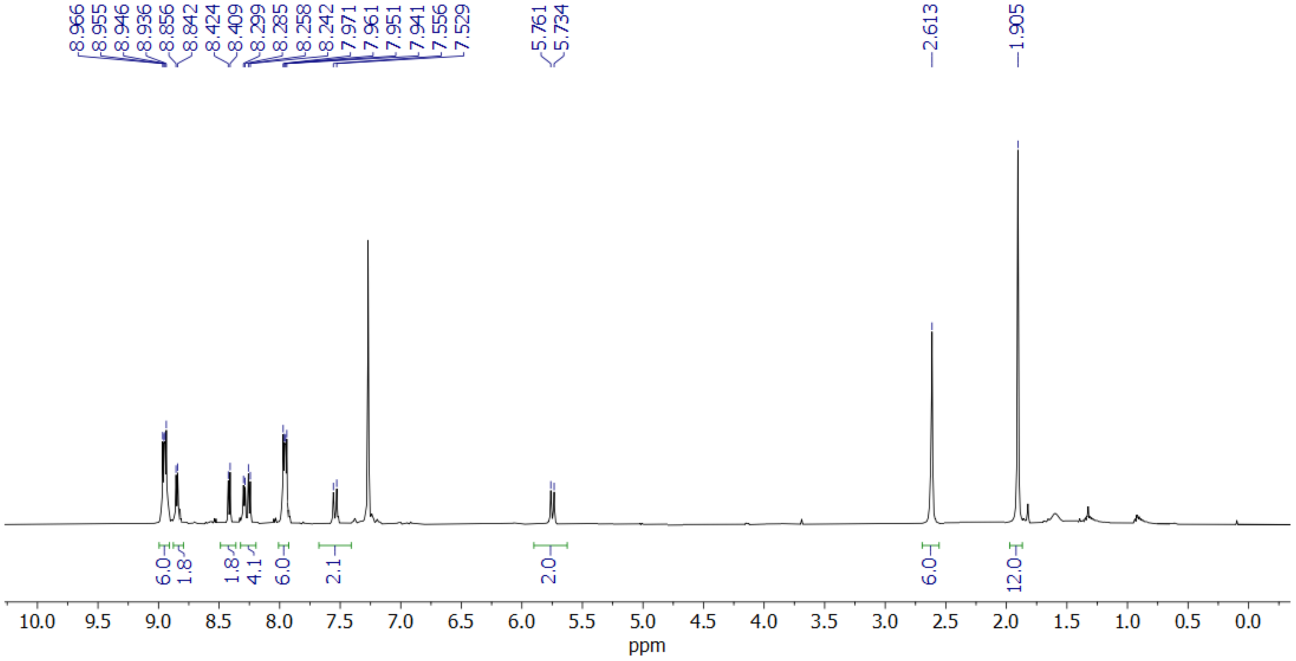

Figure S5.8. ^{1}H-NMR spectrum of ($CDCl_3$) **7**.

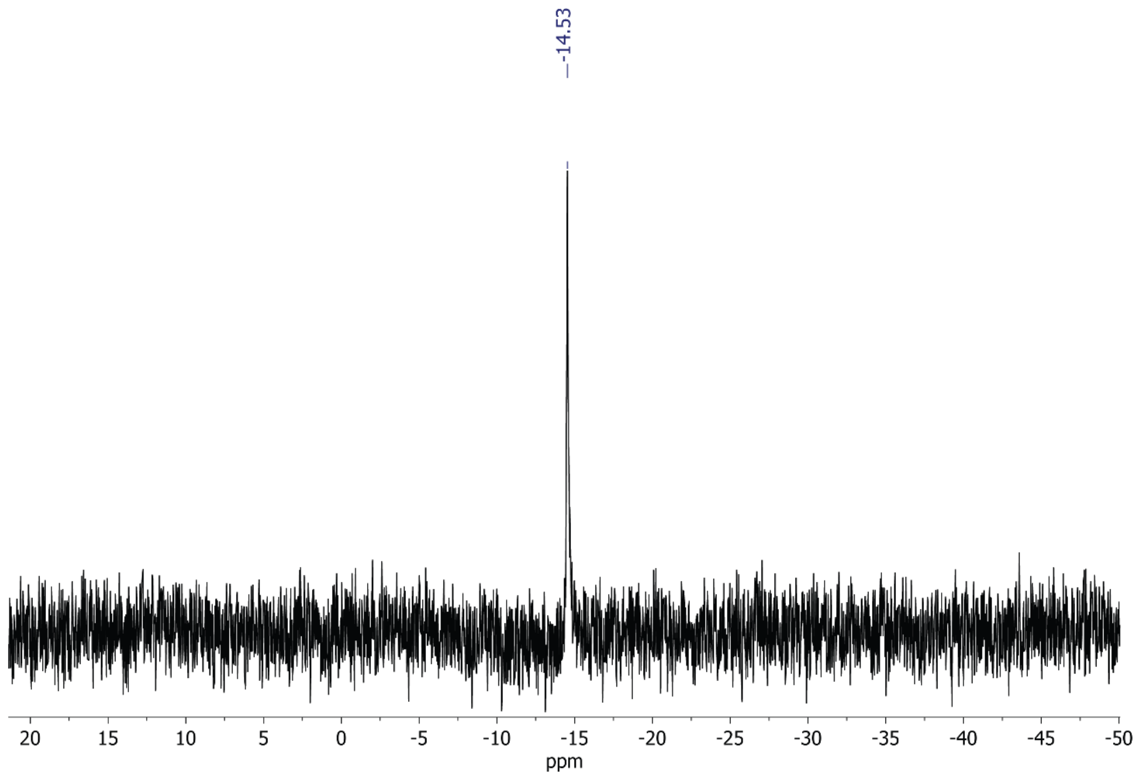

Figure S5.9. ^{11}B-NMR spectrum of ($CDCl_3$) **7**.

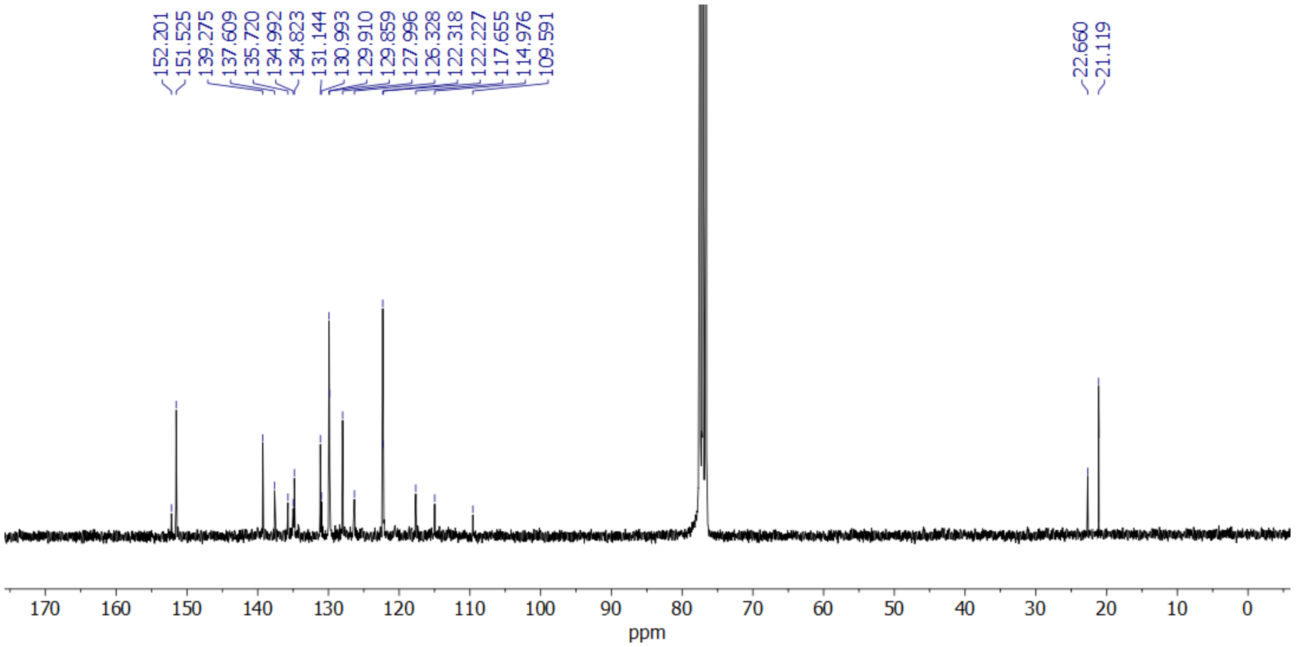

Figure S5.10. ^{13}C-NMR spectrum of ($CDCl_3$) **7**.

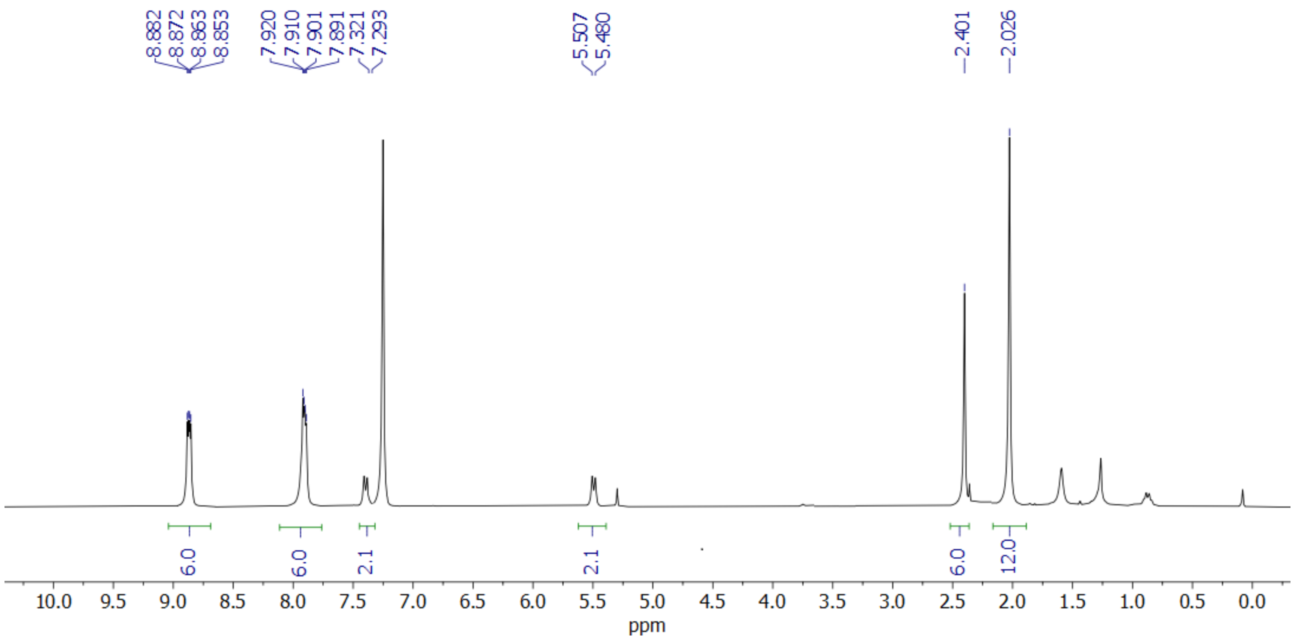

Figure S5.11. ^{1}H-NMR spectrum of ($CDCl_3$) **8**.

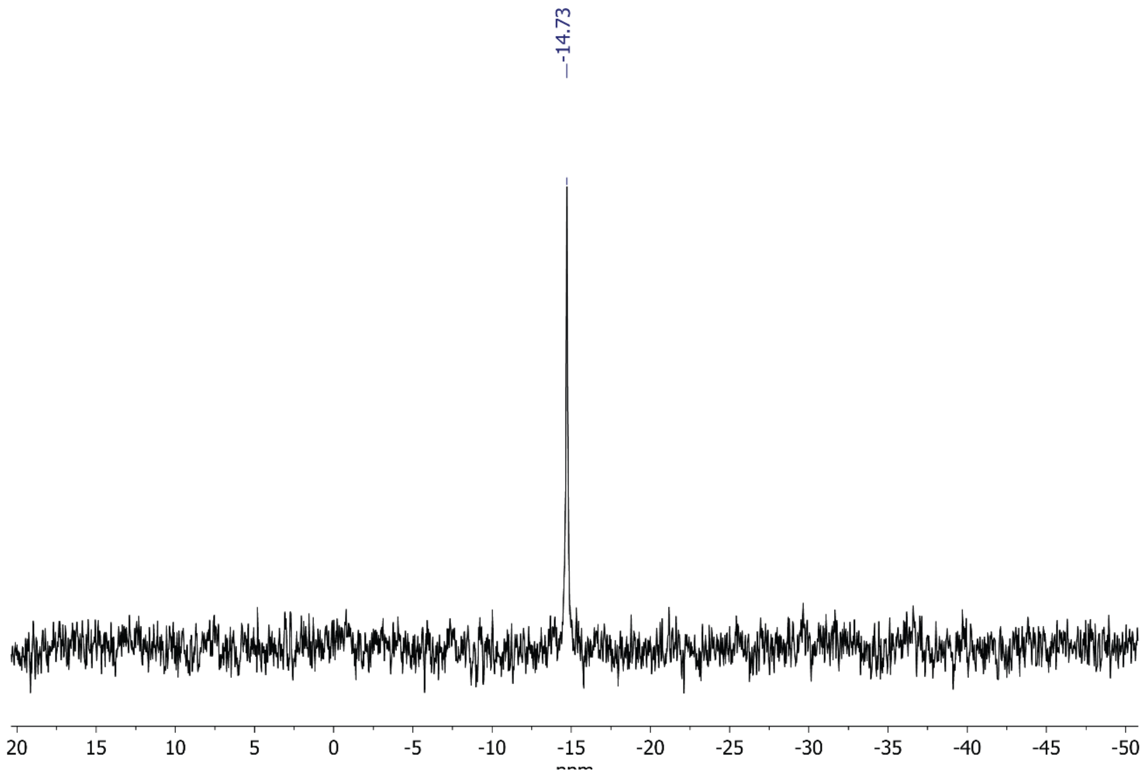

Figure S5.12. ^{11}B-NMR spectrum of ($CDCl_3$) **8**.

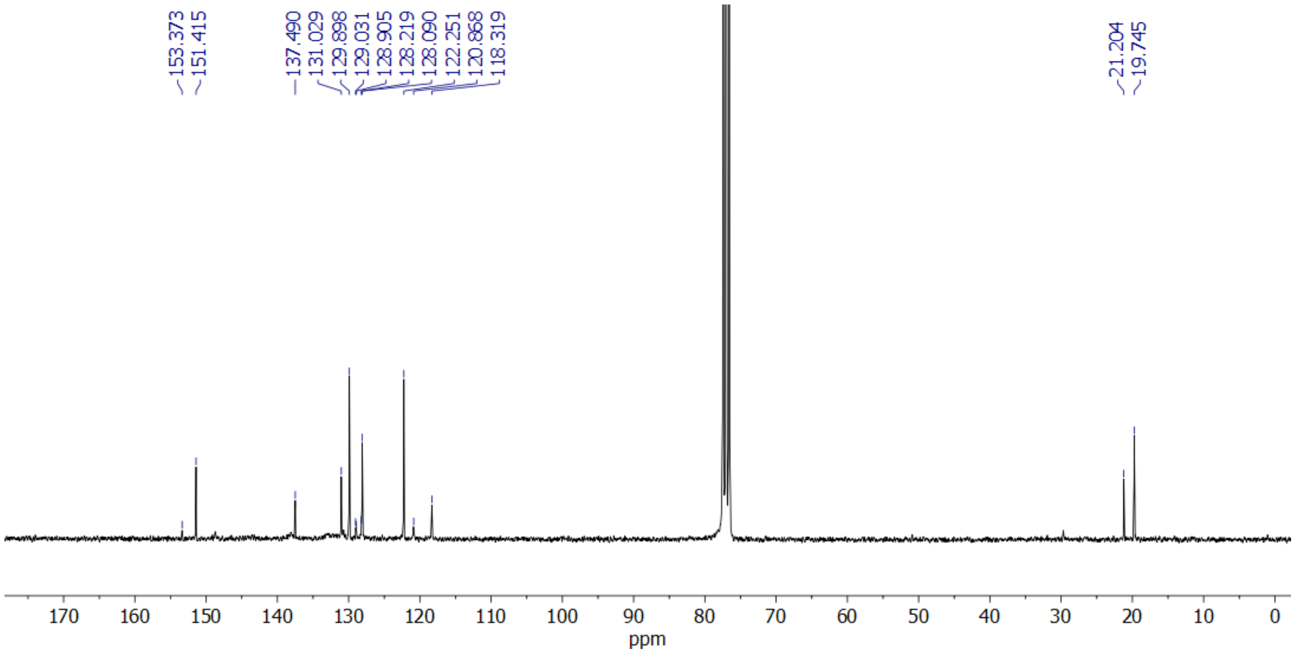

Figure S5.13. ^{13}C-NMR spectrum of ($CDCl_3$) **8**.

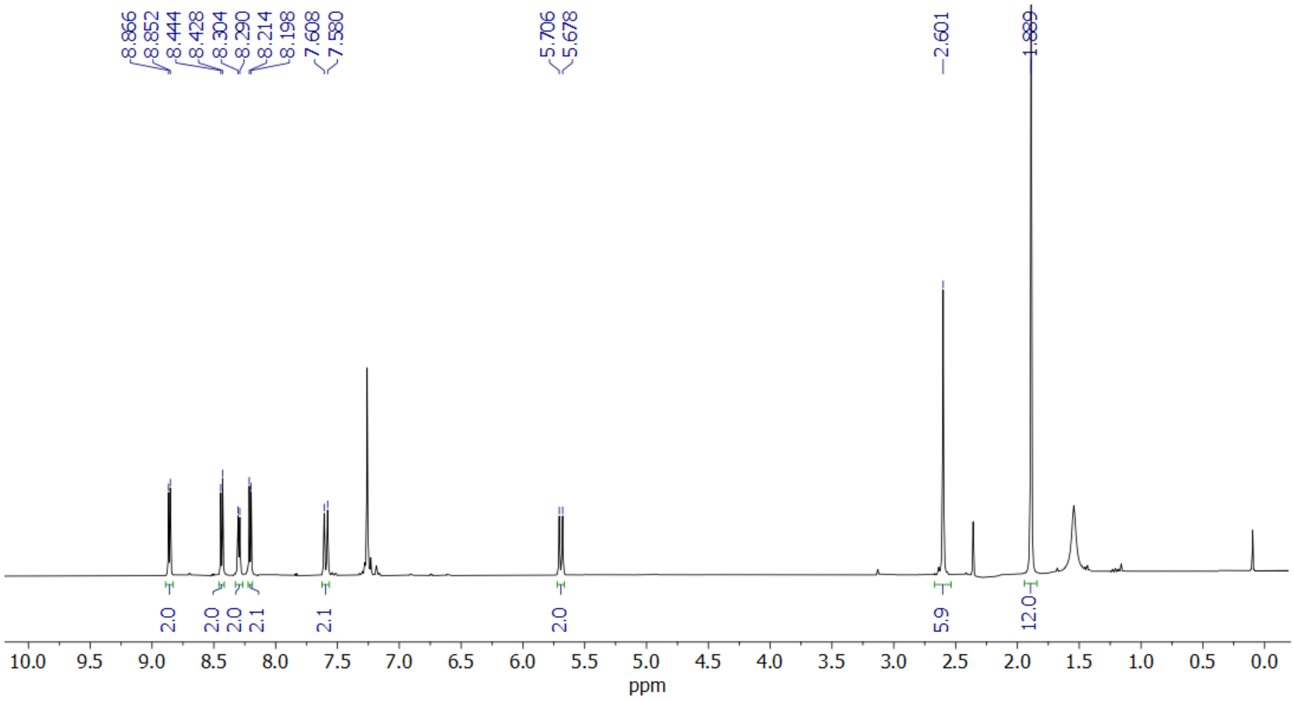

Figure S5.14. ^{1}H-NMR spectrum of ($CDCl_3$) **9**.

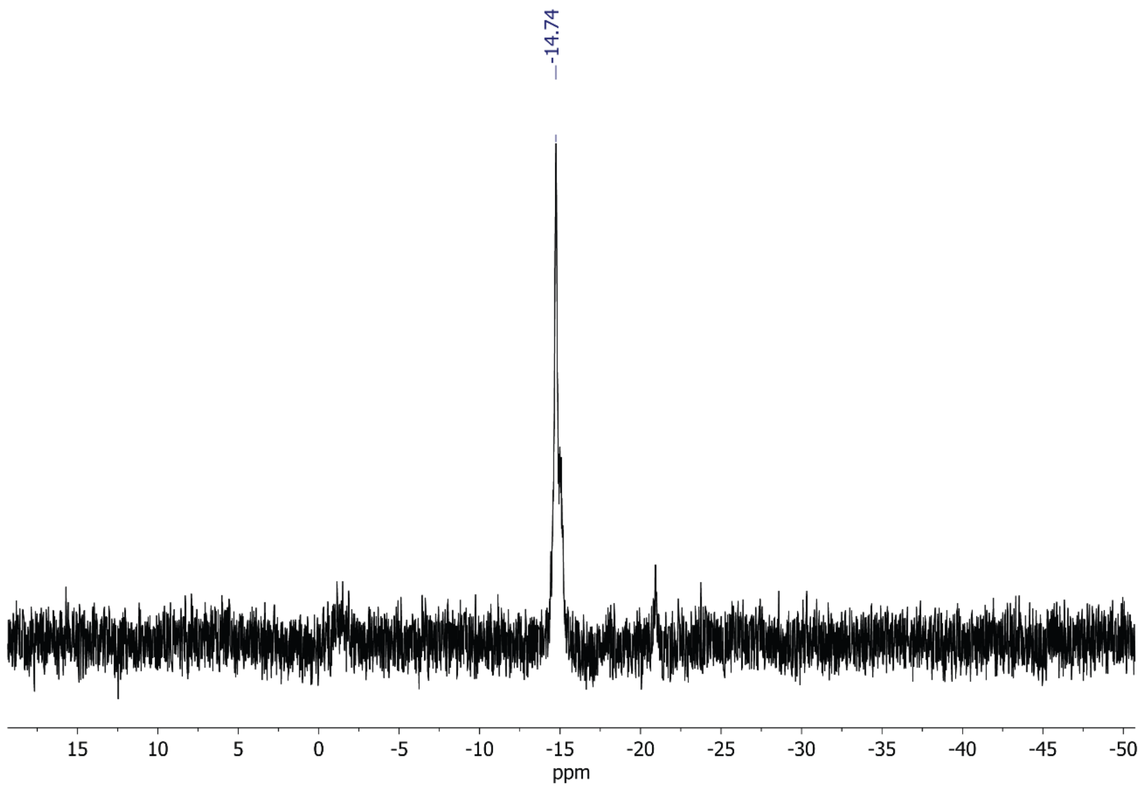

Figure S5.15. ^{11}B-NMR spectrum of ($CDCl_3$) **9**.

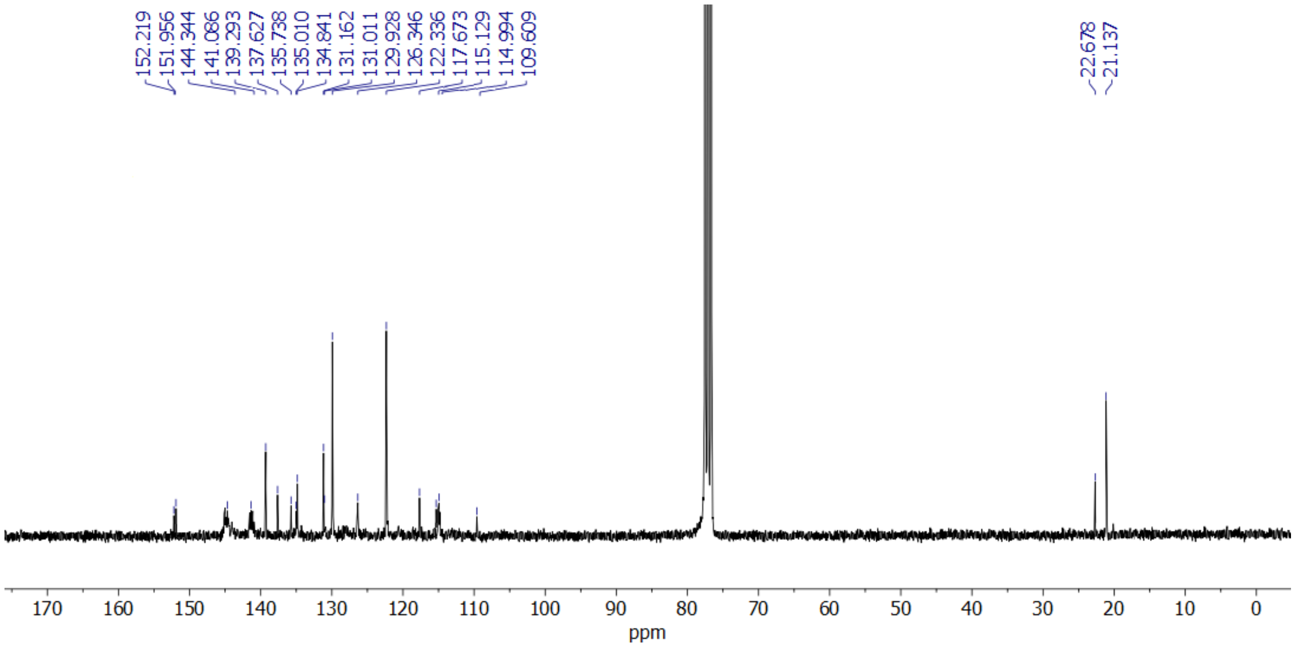

Figure S5.16. ^{13}C-NMR spectrum of ($CDCl_3$) **9**.

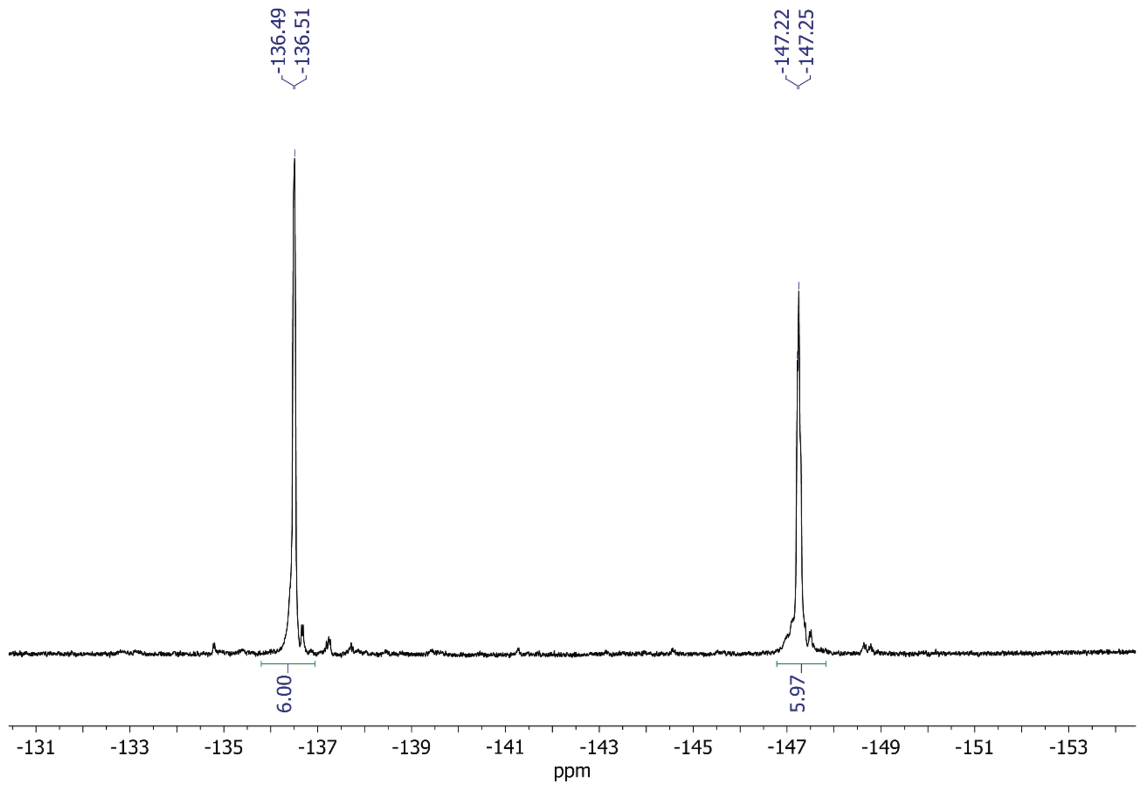

Figure S5.17. ^{19}F-NMR spectrum of ($CDCl_3$) **9**.

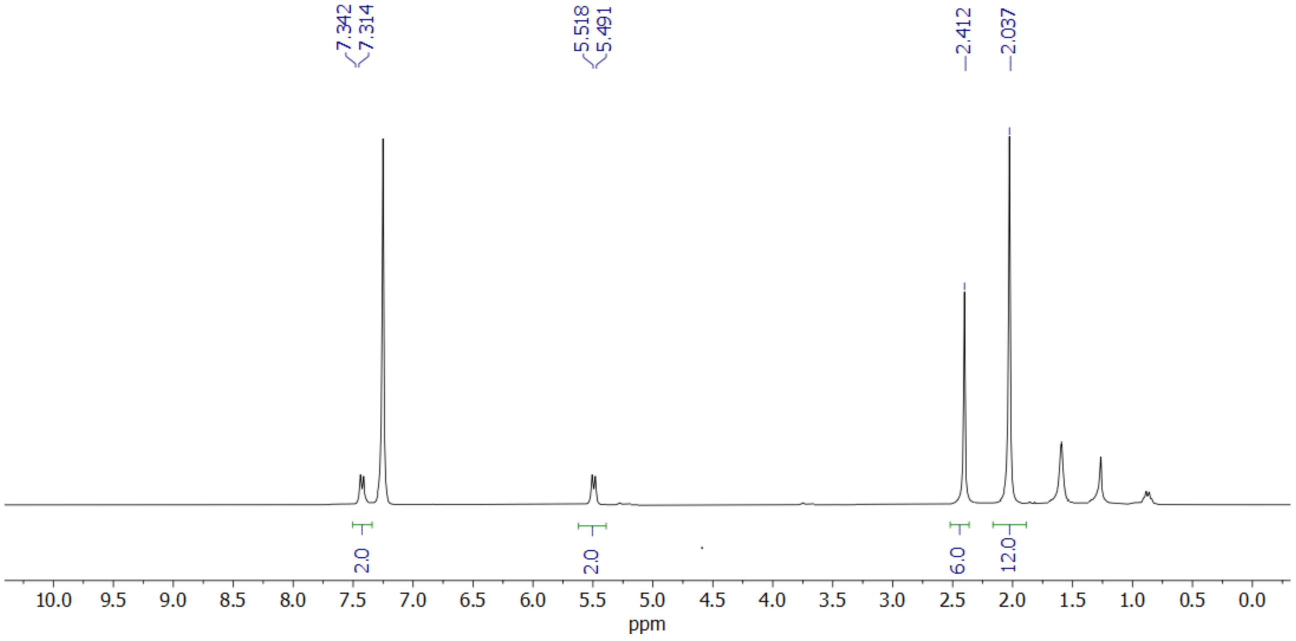

Figure S5.18. ^{1}H-NMR spectrum of ($CDCl_3$) **10**.

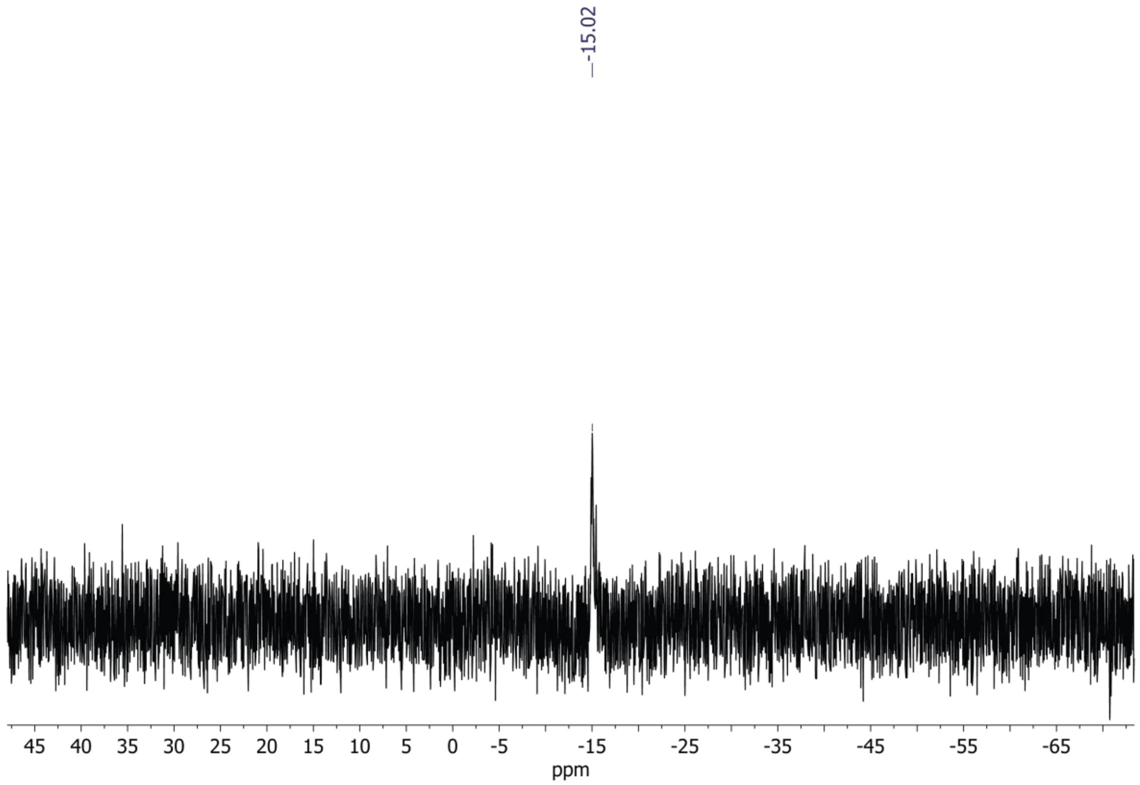

Figure S5.19. ^{11}B-NMR spectrum of ($CDCl_3$) **10**.

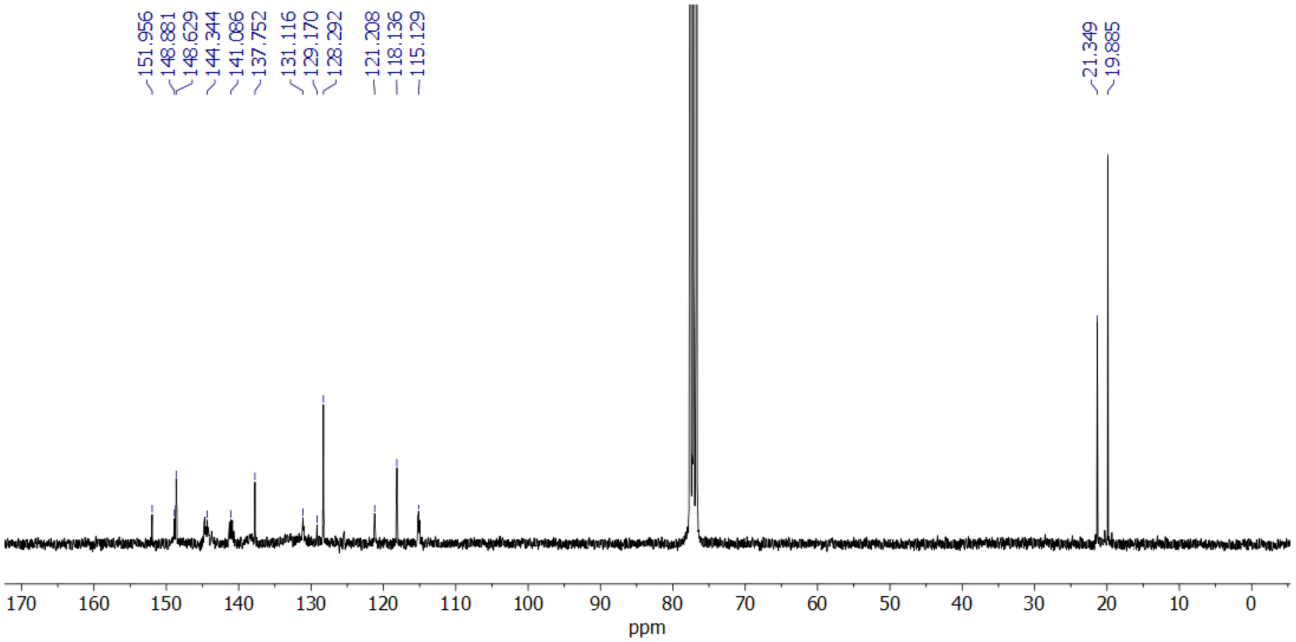

Figure S5.20. ^{13}C-NMR spectrum of ($CDCl_3$) **10**.

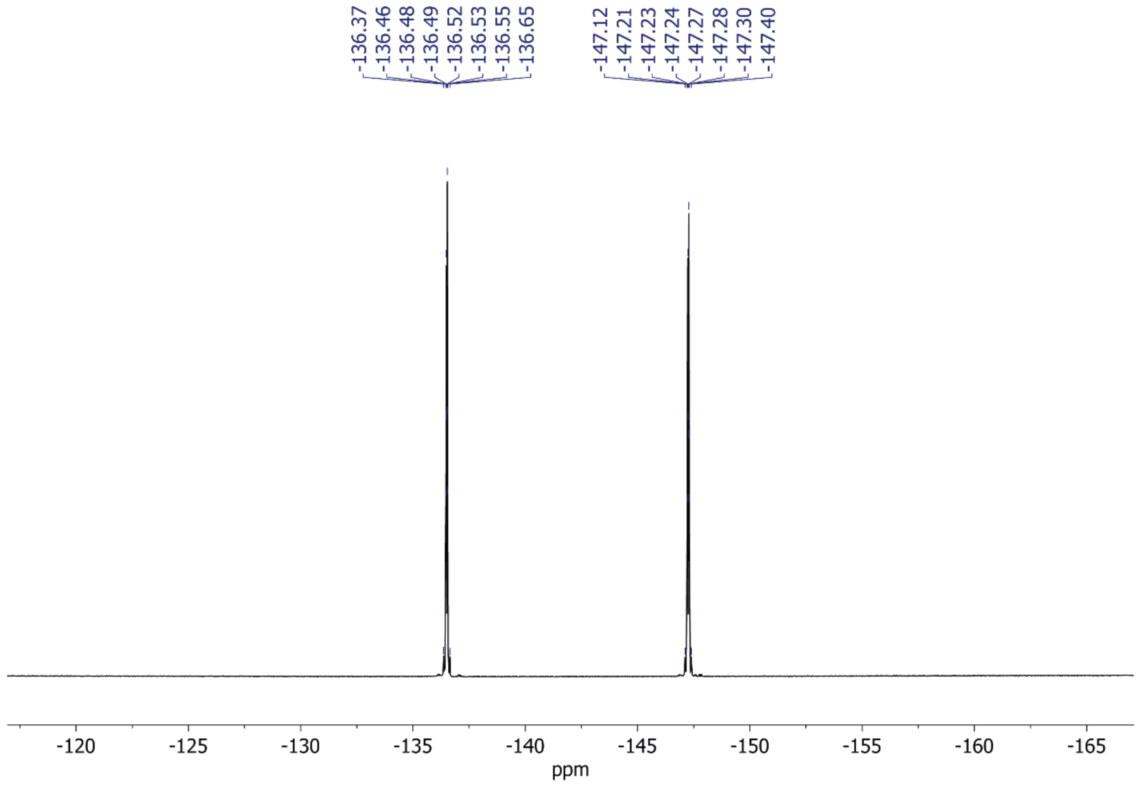

Figure S5.21. ^{19}F-NMR spectrum of ($CDCl_3$) **10**.

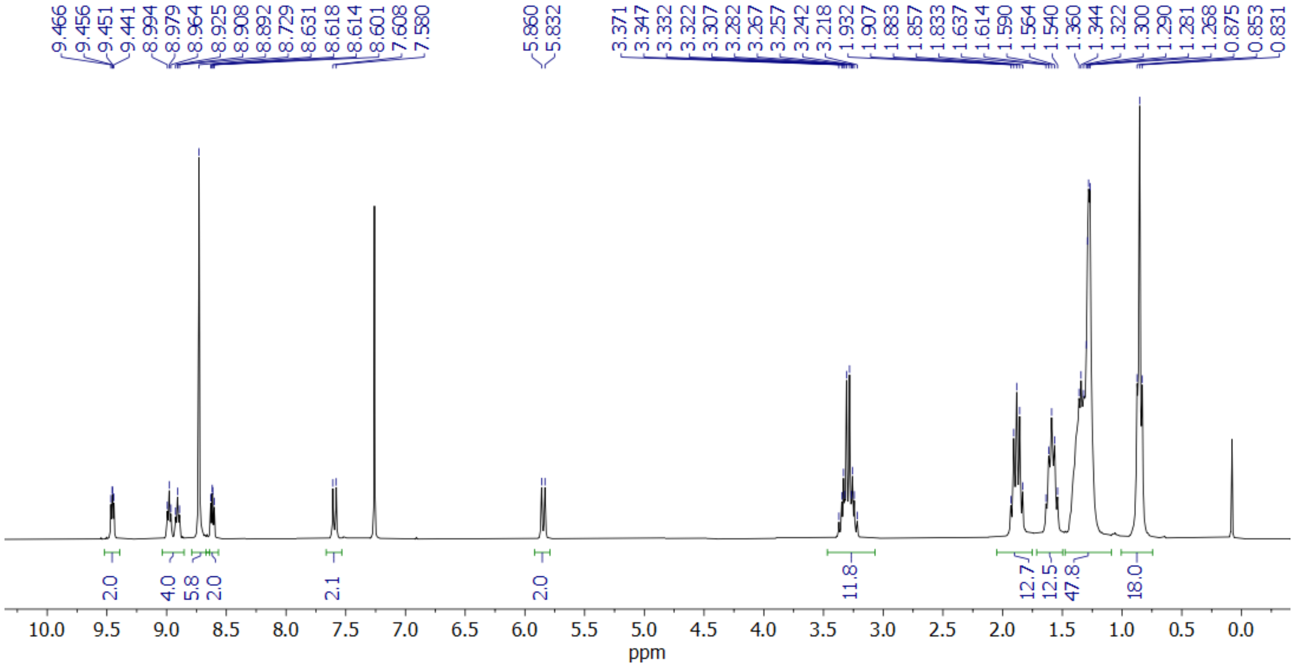

Figure S5.22. ^{1}H-NMR spectrum of ($CDCl_3$) **11**.

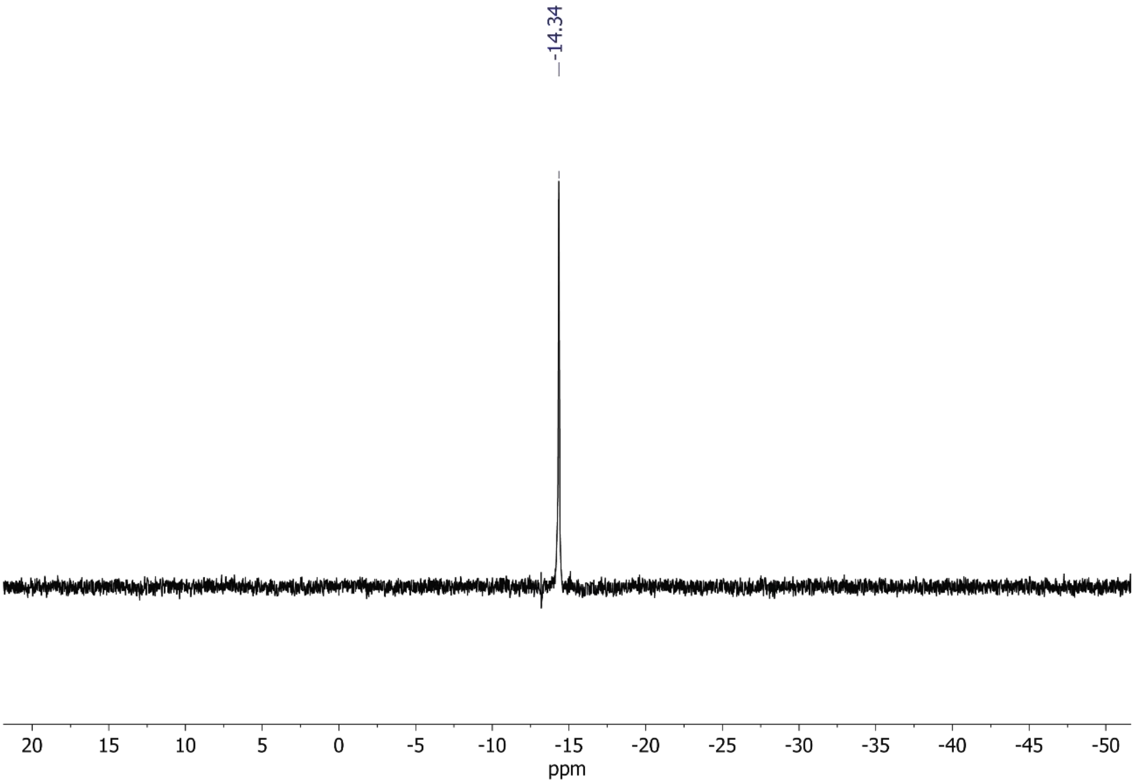

Figure S5.23. ^{11}B-NMR spectrum of ($CDCl_3$) **11**.

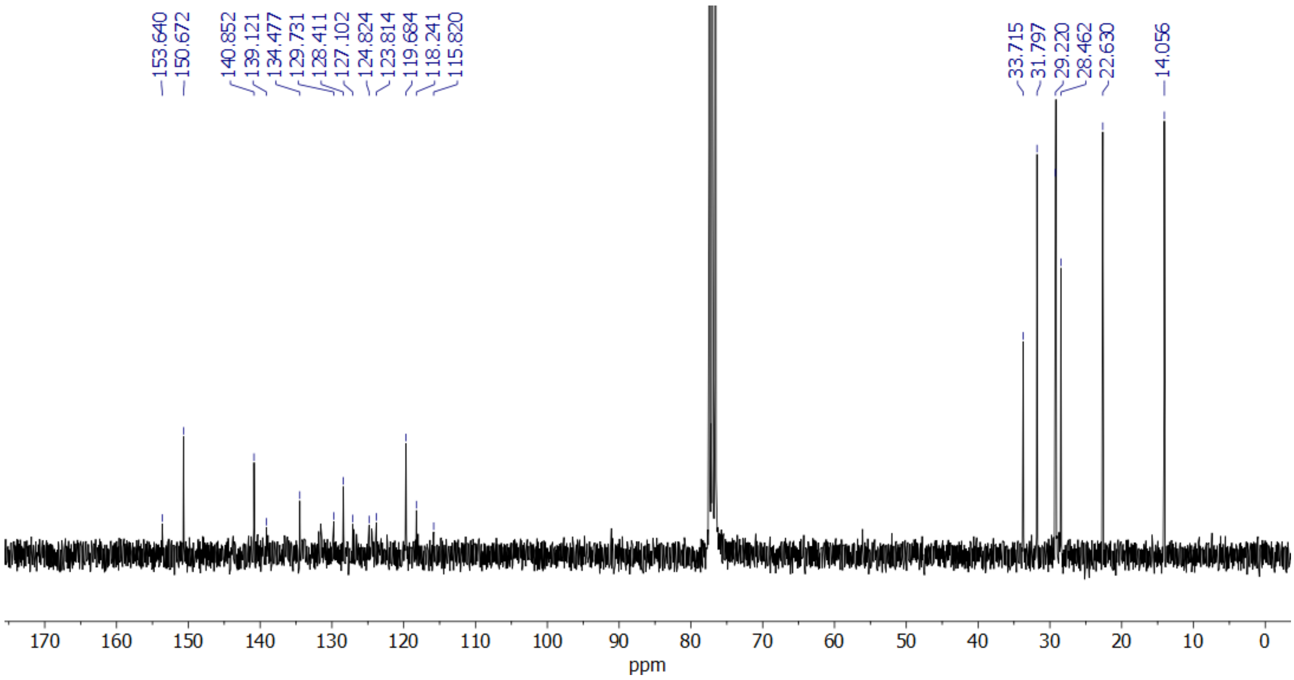

Figure S5.24. ^{13}C-NMR spectrum of ($CDCl_3$) **11**.

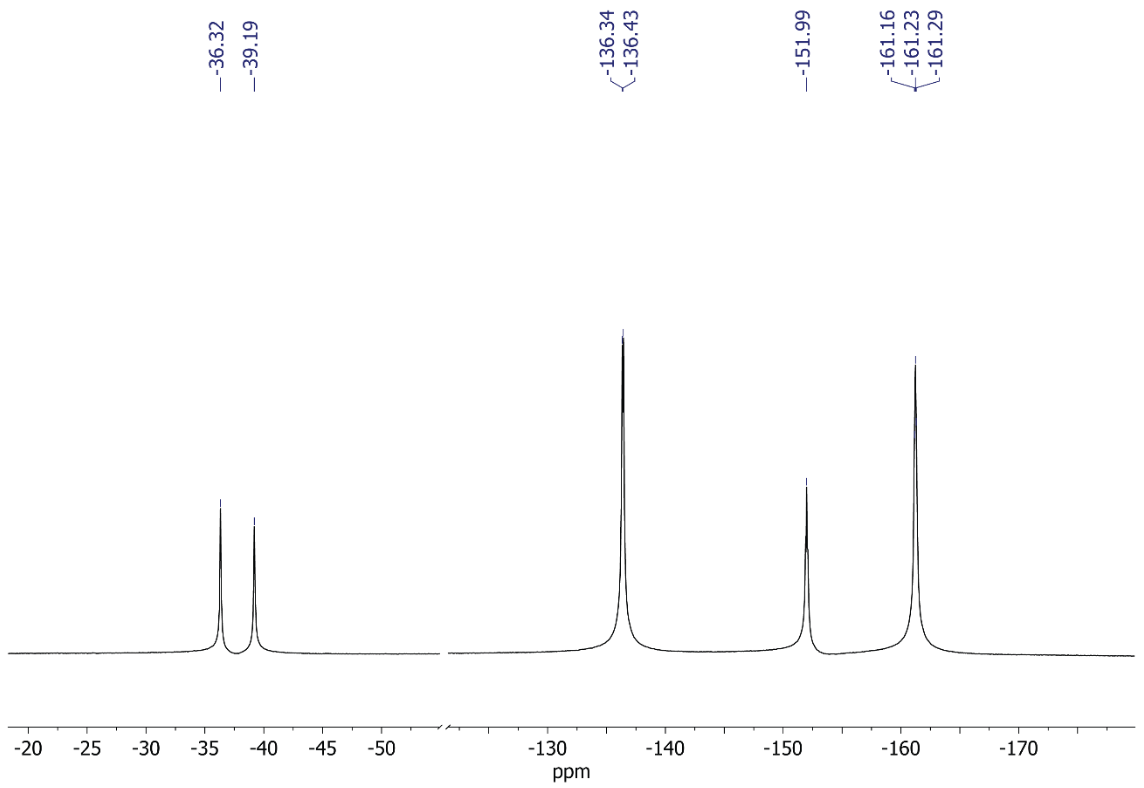

Figure S5.25. ^{19}F-NMR spectrum of ($CDCl_3$) **11**.

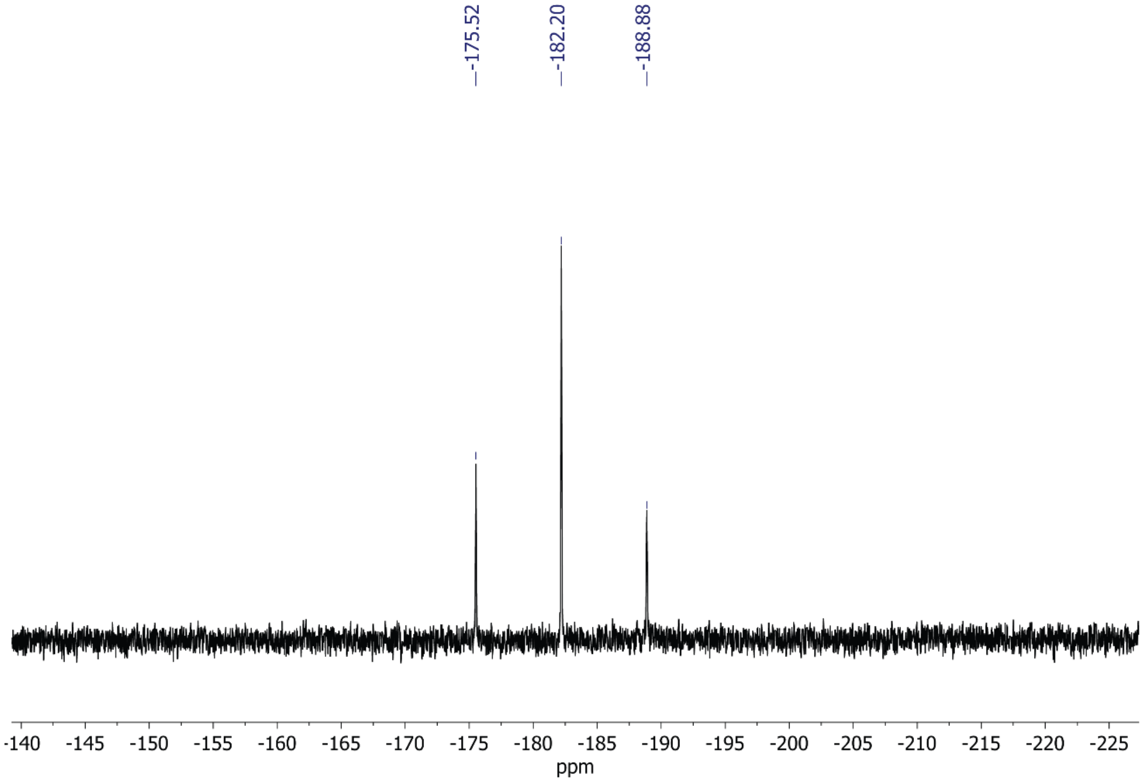

Figure S5.26. ^{31}P-NMR spectrum of ($CDCl_3$) **11**.

6. Electrochemistry & Spectroelectrochemistry

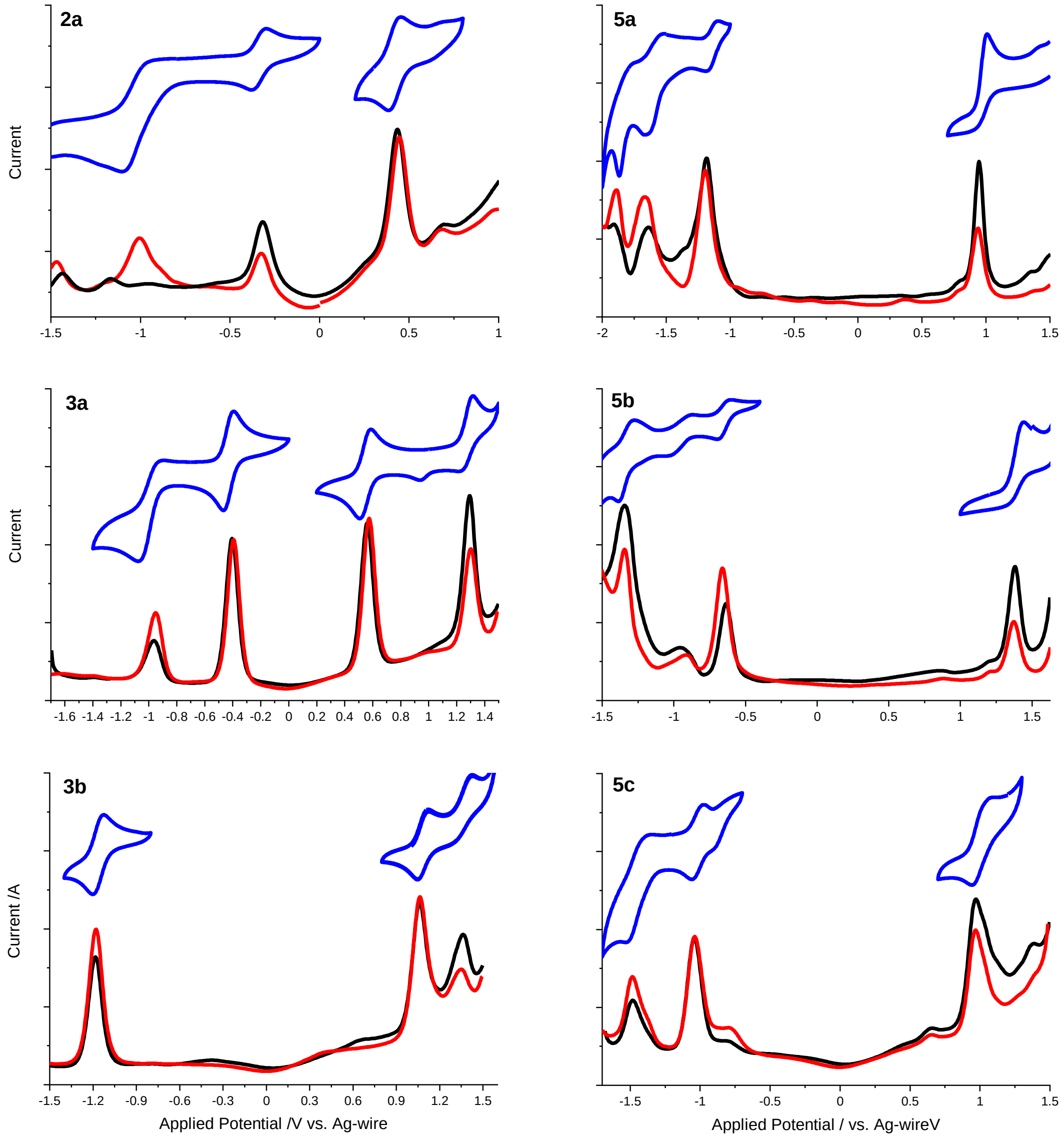

Figure S6.1. Differential pulse (upscan: black; downscan: red) and cyclic voltammograms (blue) of reference Corrs **2a**, **3a**, and **3b**, as well as SubPcs **5a-c** in de-aerated dichloromethane (0.1 M $TBAPF_6$ as electrolyte).

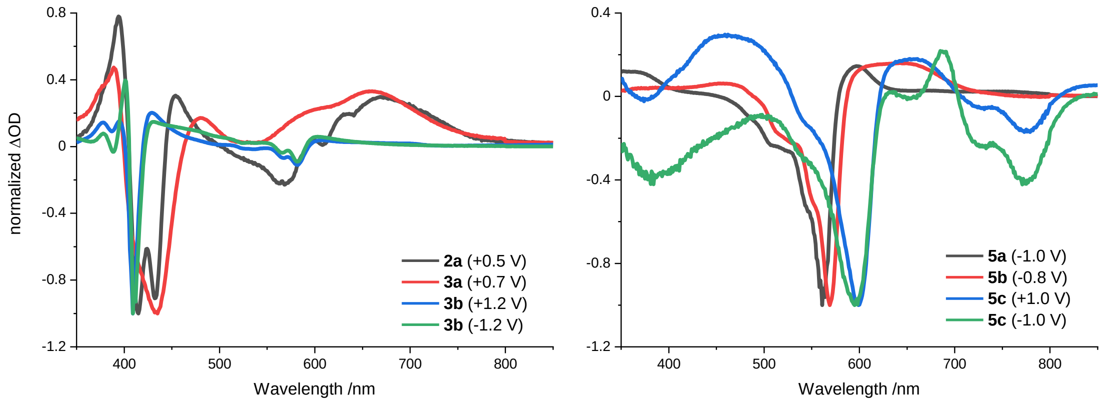

Figure S6.2. Differential absorption of one-electron oxidized and reduced Cors (left) and SubPcs (right) in dichloromethane (0.1 M $TBAPF_6$ as electrolyte) at room temperature.

7. Steady-State Absorption & Fluorescence Spectroscopy

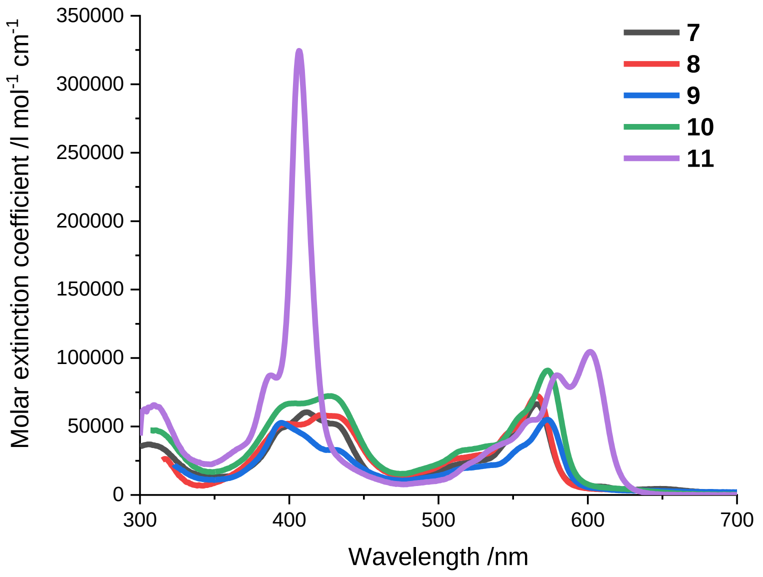

Figure S7.1. Steady-state absorption of Cor-SubPc conjugates in benzonitrile at room temperature.

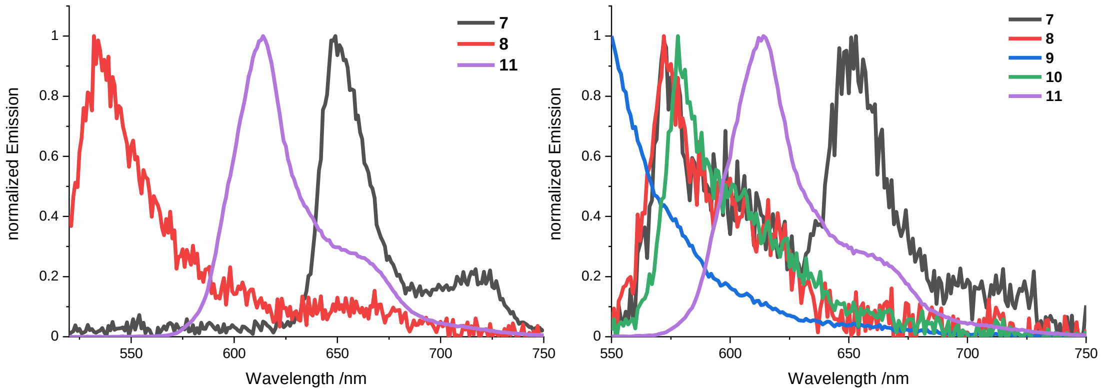

Figure S7.2. Normalized steady-state fluorescence of Cor-SubPc conjugates upon 420 nm (left) and 540 nm (right) excitation in benzonitrile at room temperature.

8. Femtosecond Transient Absorption Spectroscopy

Reference Cors and SubPcs:

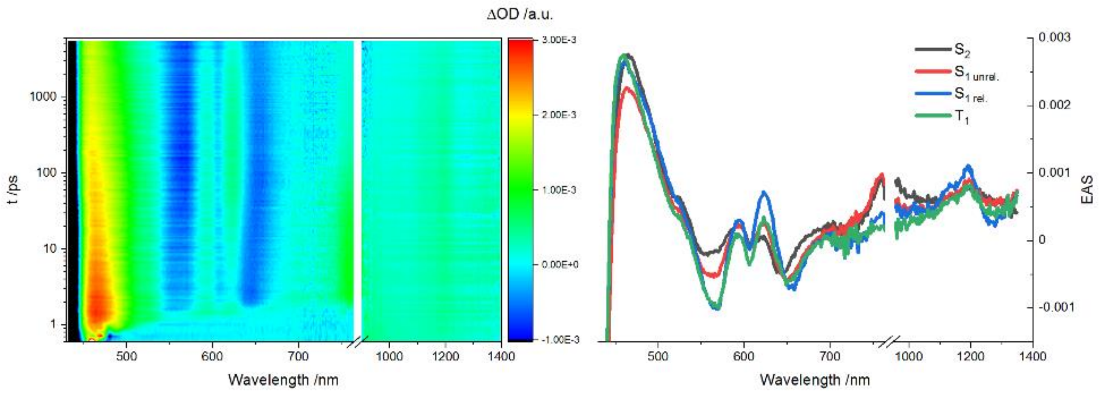

Figure S8.1. Differential absorption of **2a** upon femtosecond flash photolysis with excitation at 420 nm in toluene (left) and corresponding evolution associated spectra of the involved transient species (right).

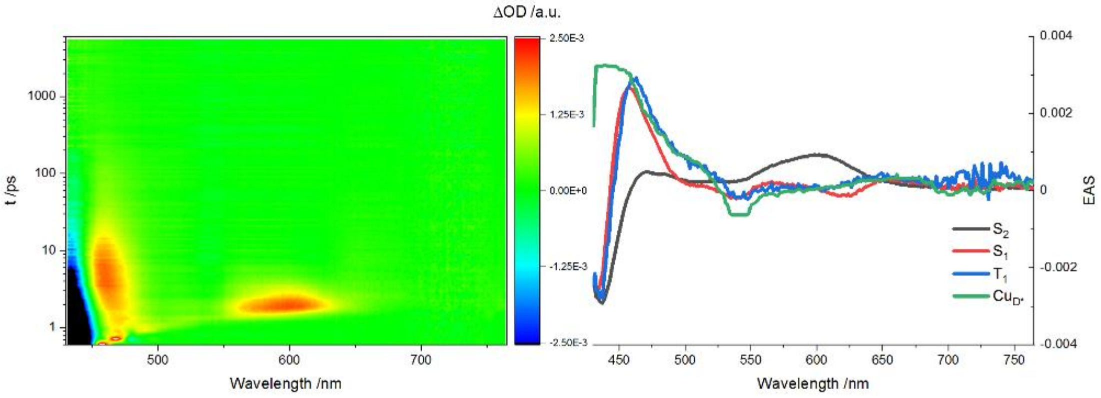

Figure S8.2. Differential absorption of **3a** upon femtosecond flash photolysis with excitation at 420 nm in toluene (left) and corresponding evolution associated spectra of the involved transient species (right).

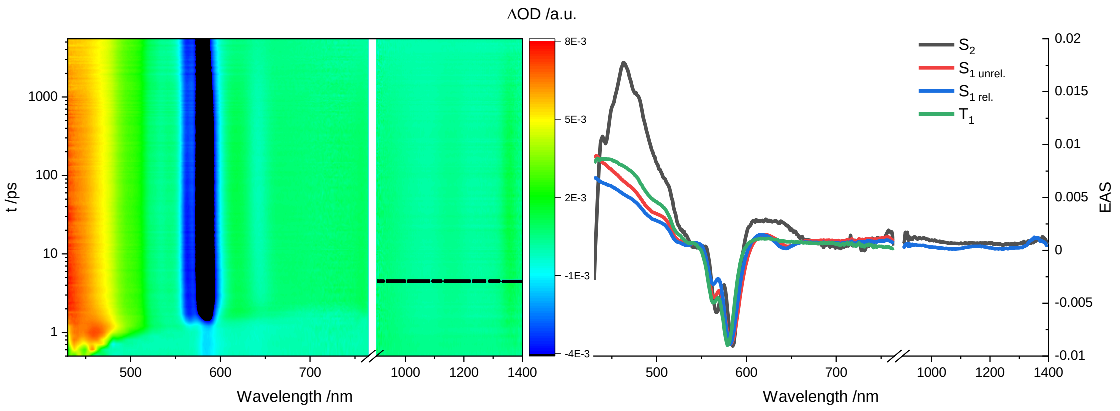

Figure S8.3. Differential absorption of **3b** upon femtosecond flash photolysis with excitation at 420 nm in toluene (left) and corresponding evolution associated spectra of the involved transient species (right).

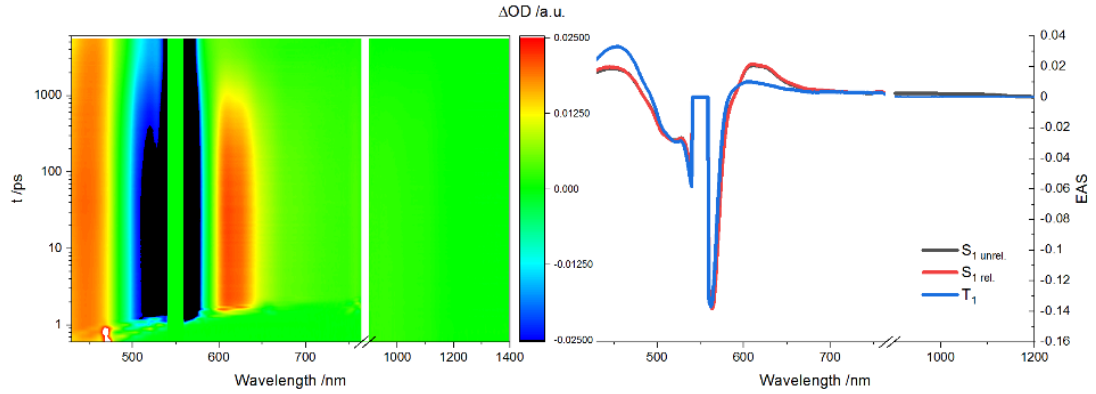

Figure S8.4. Differential absorption of **5a** upon femtosecond flash photolysis with excitation at 550 nm in toluene (left) and corresponding evolution associated spectra of the involved transient species (right).

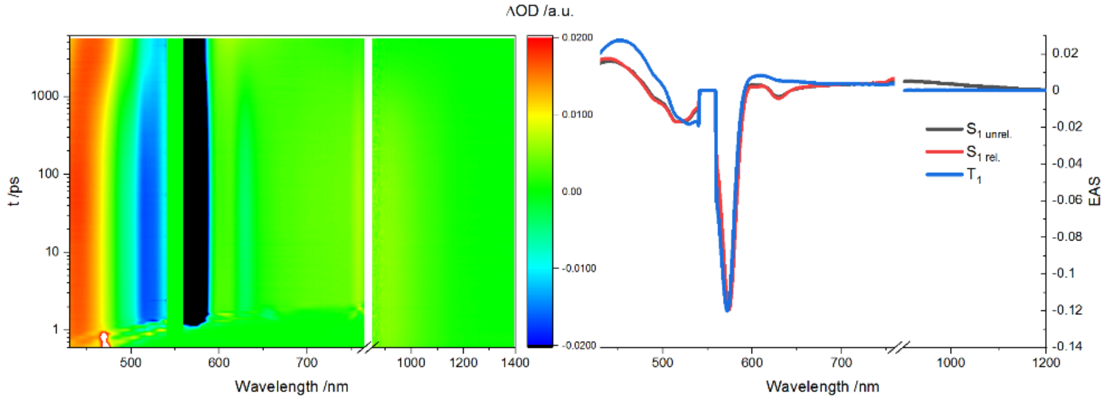

Figure S8.5. Differential absorption of **5b** upon femtosecond flash photolysis with excitation at 550 nm in toluene (left) and corresponding evolution associated spectra of the involved transient species (right).

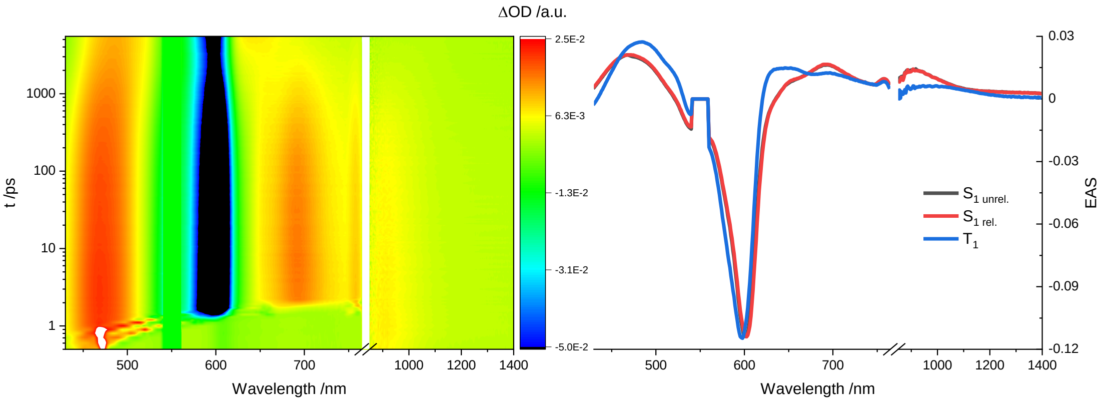

Figure S8.6. Differential absorption of **5c** upon femtosecond flash photolysis with excitation at 550 nm in toluene (left) and corresponding evolution associated spectra of the involved transient species (right).

SubPc-Corrole dyads **7-11**:

SubPc-Cor **7**

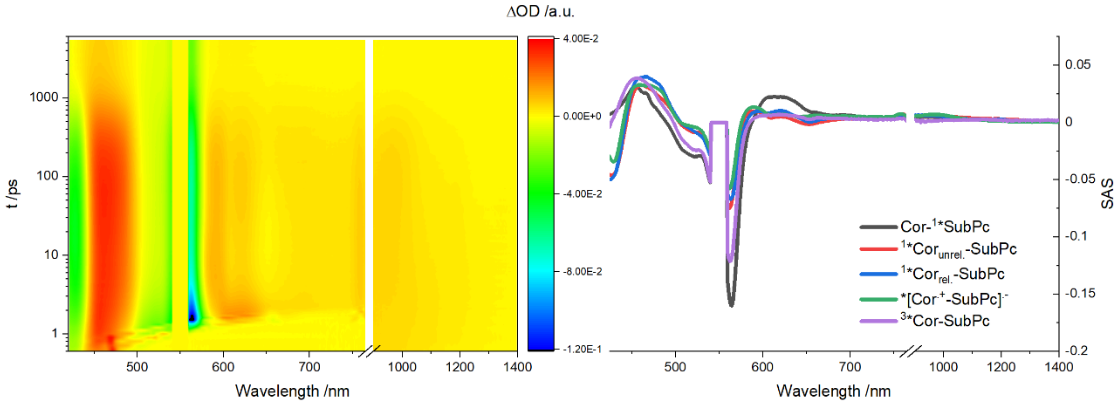

Figure S8.7. Differential absorption of **7** upon femtosecond flash photolysis with excitation at 550 nm in toluene (left) and corresponding species associated spectra of the involved transient species (right).

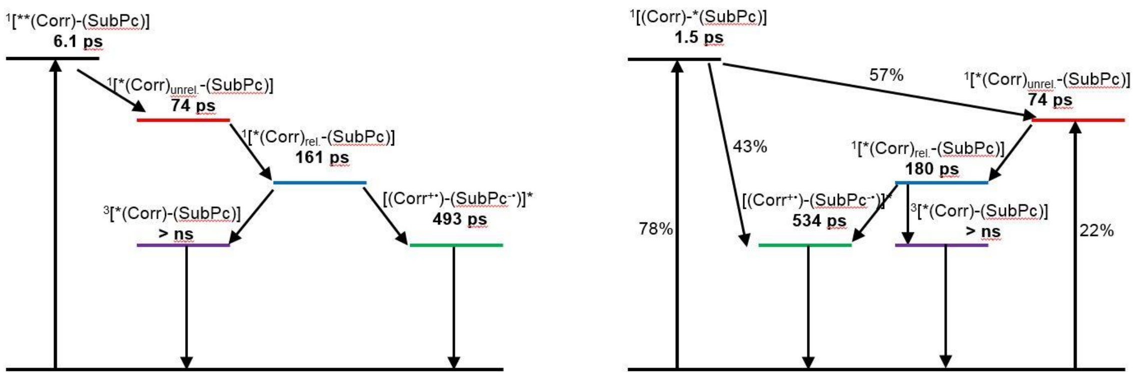

Figure S8.8. Kinetic models used for the analysis of femtosecond-resolved differential absorption of **7** upon excitation at 420 (left) and 550 nm (right) in toluene.

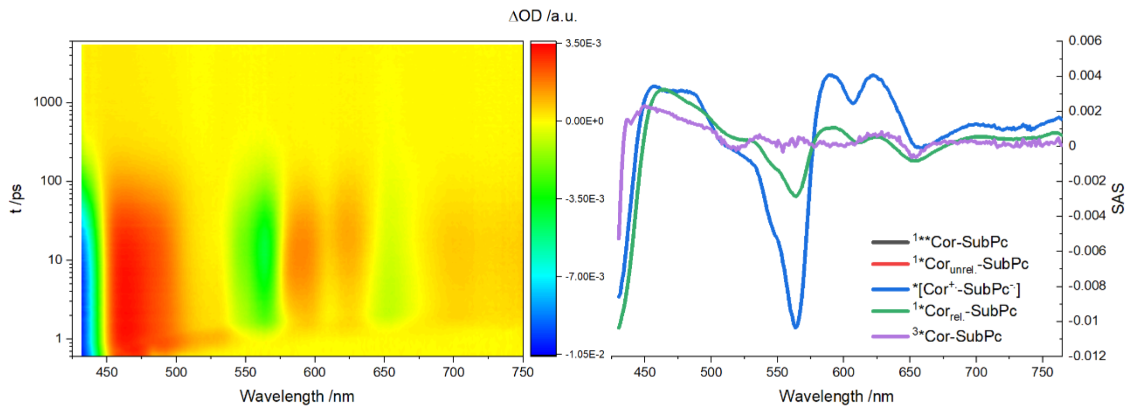

Figure S8.9. Differential absorption of **7** upon femtosecond flash photolysis with excitation at 420 nm in benzonitrile (left) and corresponding species associated spectra of the involved transient species (right).

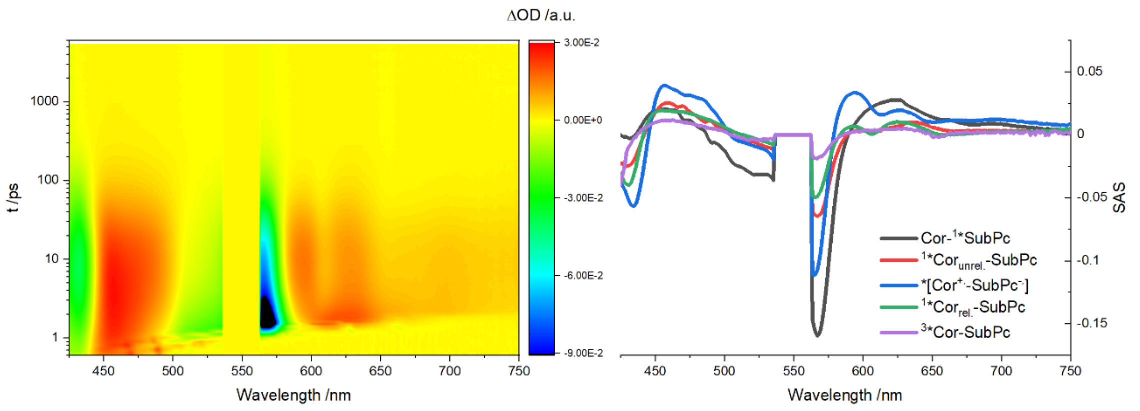

Figure S8.10. Differential absorption of **7** upon femtosecond flash photolysis with excitation at 550 nm in benzonitrile (left) and corresponding species associated spectra of the involved transient species (right).

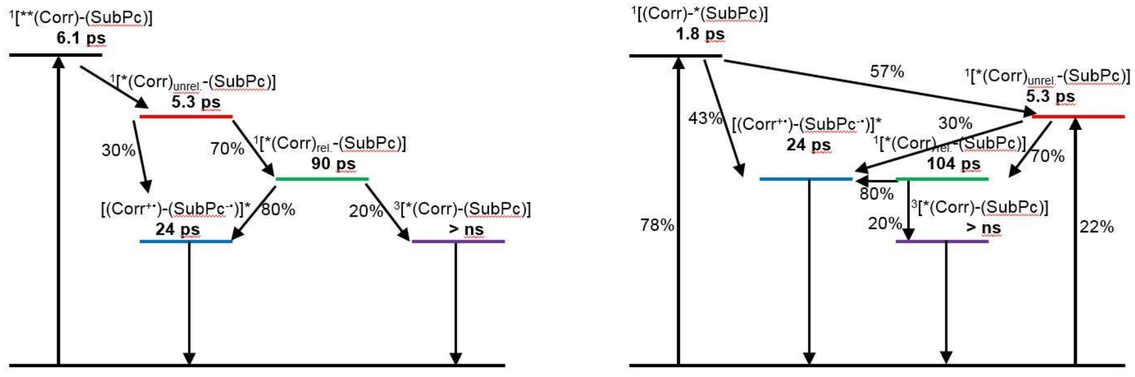

Figure S8.11. Kinetic models used for the analysis of femtosecond-resolved differential absorption of **7** upon excitation at 420 (left) and 550 nm (right) in benzonitrile.

SubPc-Cor **9**

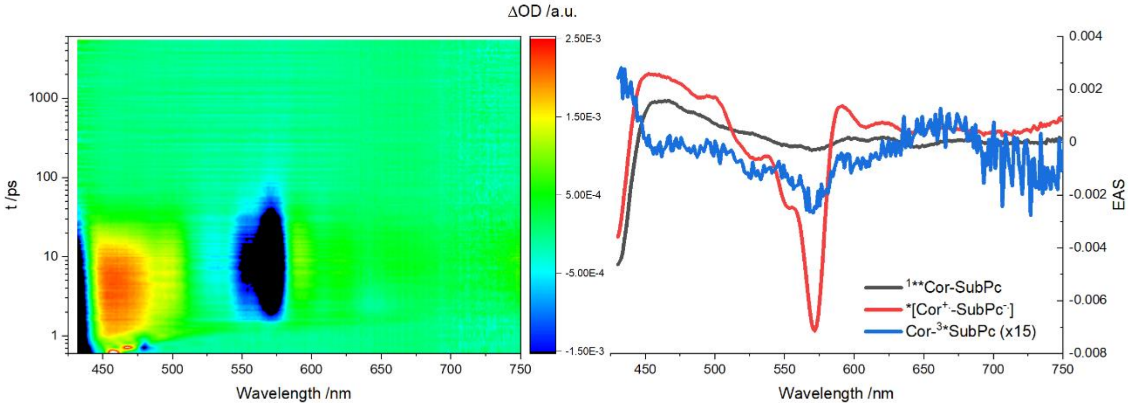

Figure S8.12. Differential absorption of **9** upon femtosecond flash photolysis with excitation at 420 nm in toluene (left) and corresponding evolution associated spectra of the involved transient species (right).

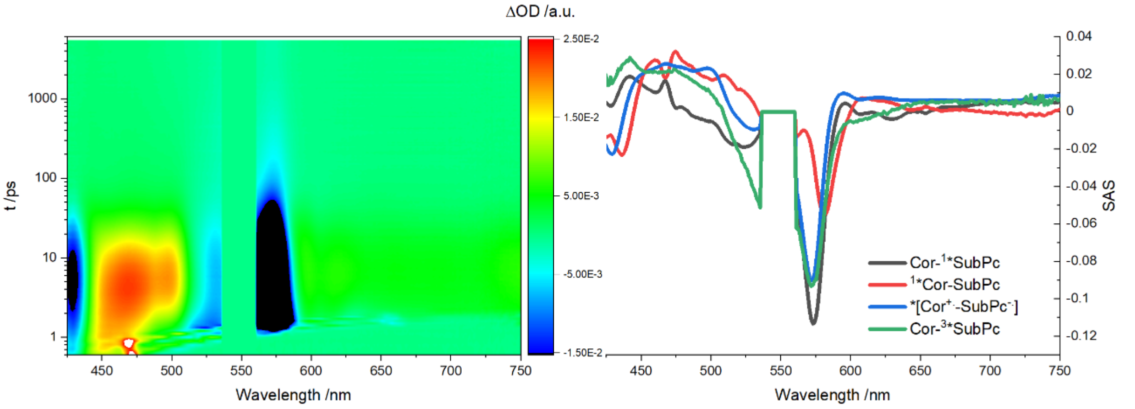

Figure S8.13. Differential absorption of **9** upon femtosecond flash photolysis with excitation at 550 nm in toluene (left) and corresponding species associated spectra of the involved transient species (right).

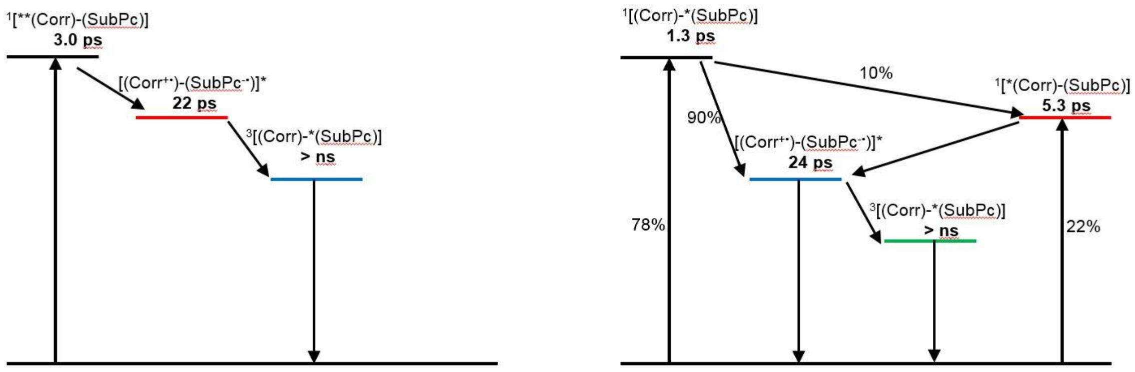

Figure S8.14. Kinetic models used for the analysis of femtosecond-resolved differential absorption of **9** upon excitation at 420 (left) and 550 nm (right) in toluene.

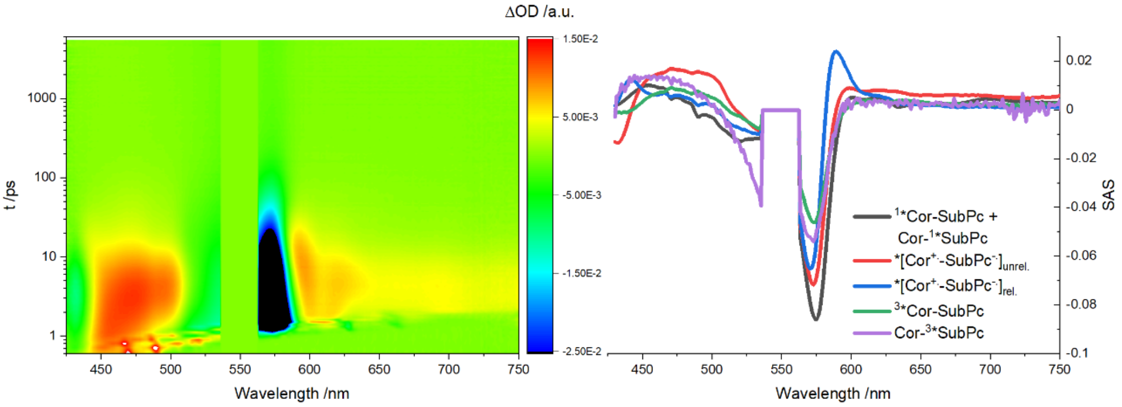

Figure S8.15. Differential absorption of **9** upon femtosecond flash photolysis with excitation at 550 nm in benzonitrile (left) and corresponding species associated spectra of the involved transient species (right).

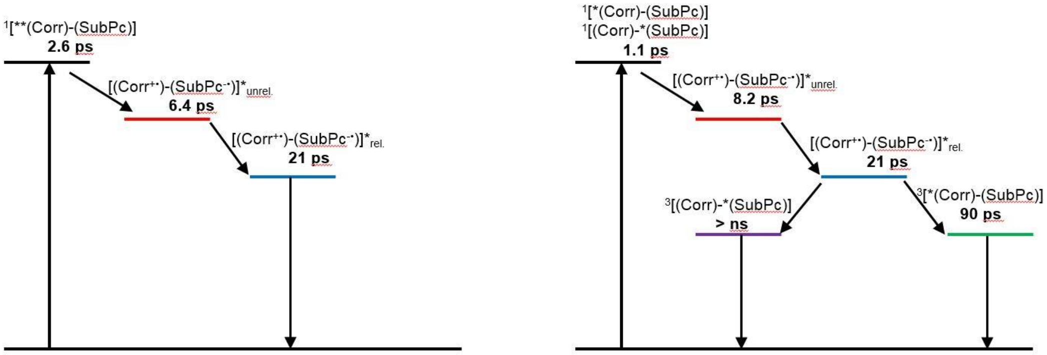

Figure S8.16. Kinetic models used for the analysis of femtosecond-resolved differential absorption of **9** upon excitation at 420 (left) and 550 nm (right) in benzonitrile.

SubPc-Cor **8**

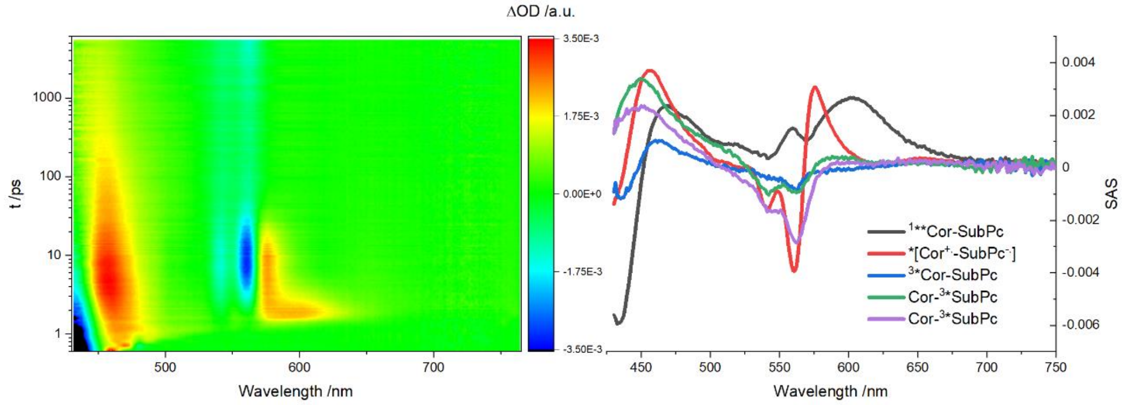

Figure S8.17. Differential absorption of **8** upon femtosecond flash photolysis with excitation at 420 nm in toluene (left) and corresponding species associated spectra of the involved transient species (right).

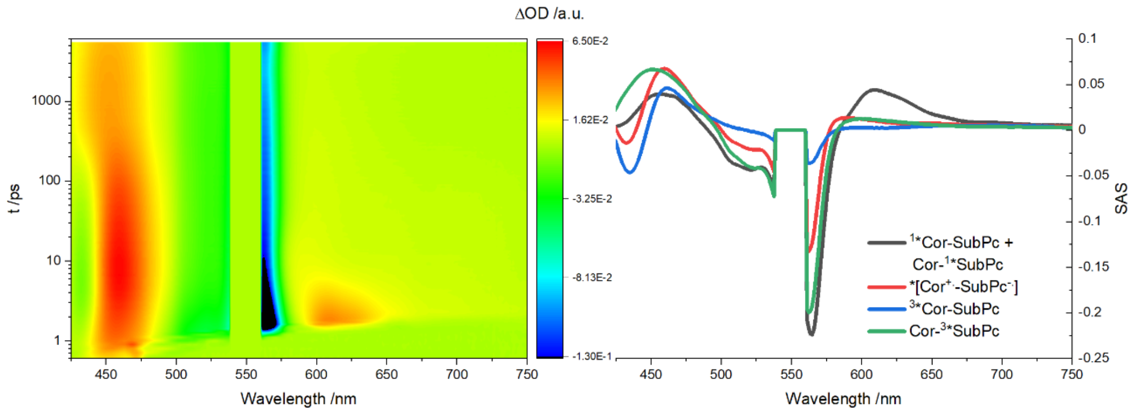

Figure S8.18. Differential absorption of **8** upon femtosecond flash photolysis with excitation at 550 nm in toluene (left) and corresponding species associated spectra of the involved transient species (right).

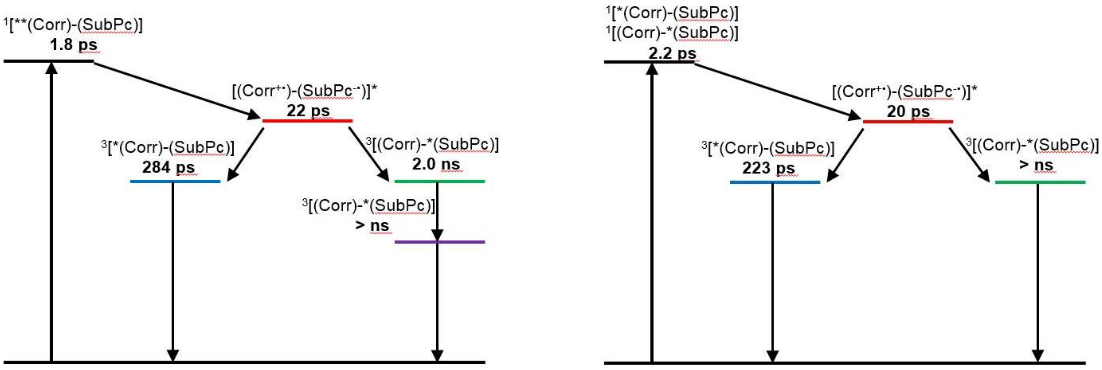

Figure S8.19. Kinetic models used for the analysis of femtosecond-resolved differential absorption of **8** upon excitation at 420 (left) and 550 nm (right) in toluene.

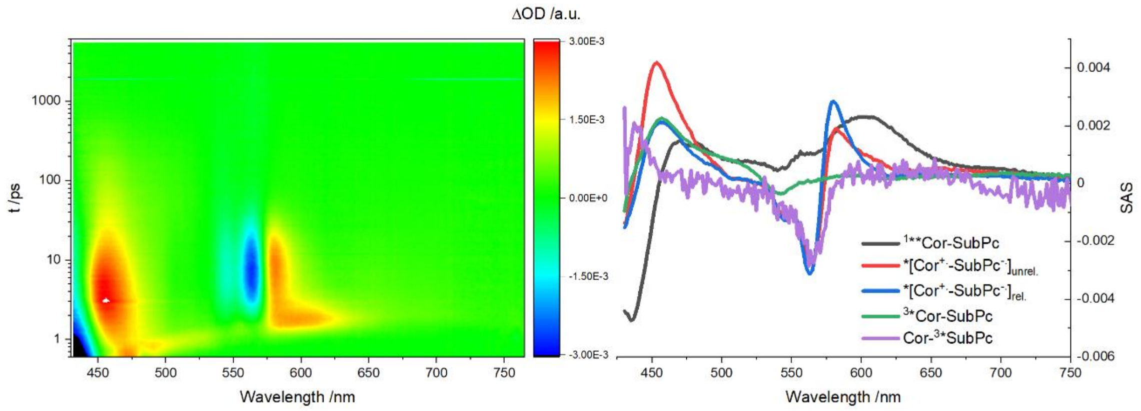

Figure S8.20. Differential absorption of **8** upon femtosecond flash photolysis with excitation at 420 nm in benzonitrile (left) and corresponding species associated spectra of the involved transient species (right).

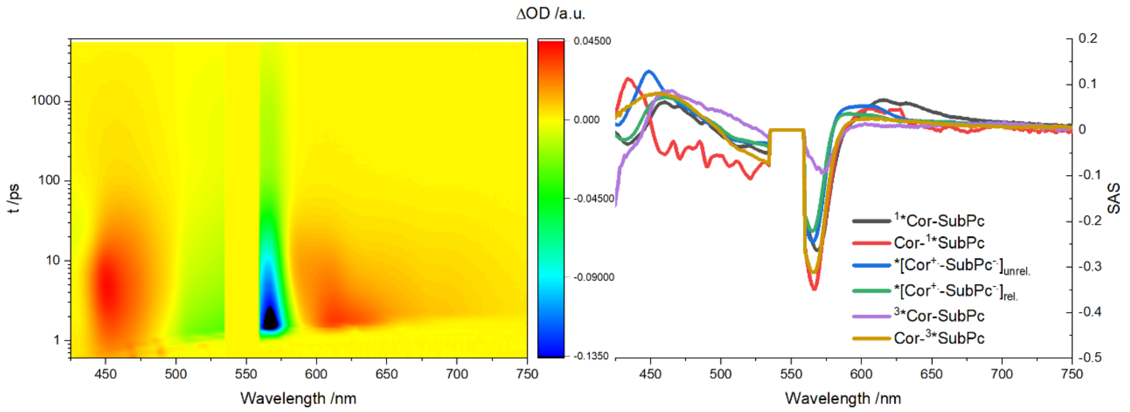

Figure S8.21. Differential absorption of **8** upon femtosecond flash photolysis with excitation at 550 nm in benzonitrile (left) and corresponding species associated spectra of the involved transient species (right).

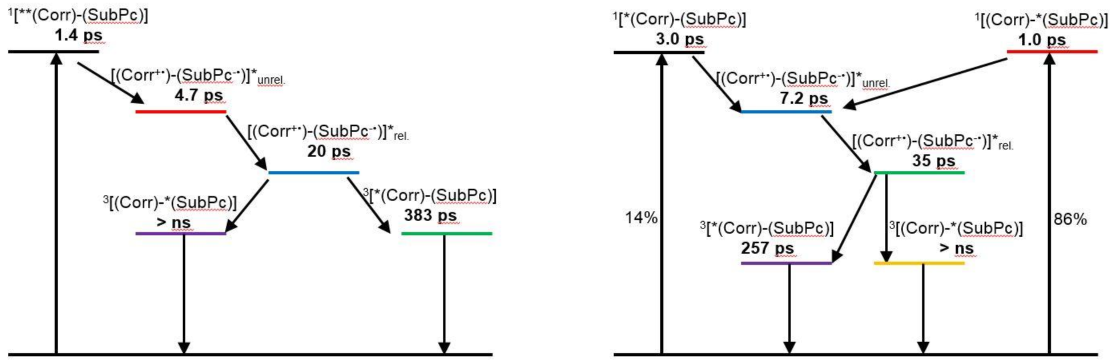

Figure S8.22. Kinetic models used for the analysis of femtosecond-resolved differential absorption of **8** upon excitation at 420 (left) and 550 nm (right) in benzonitrile.

SubPc-Cor **10**

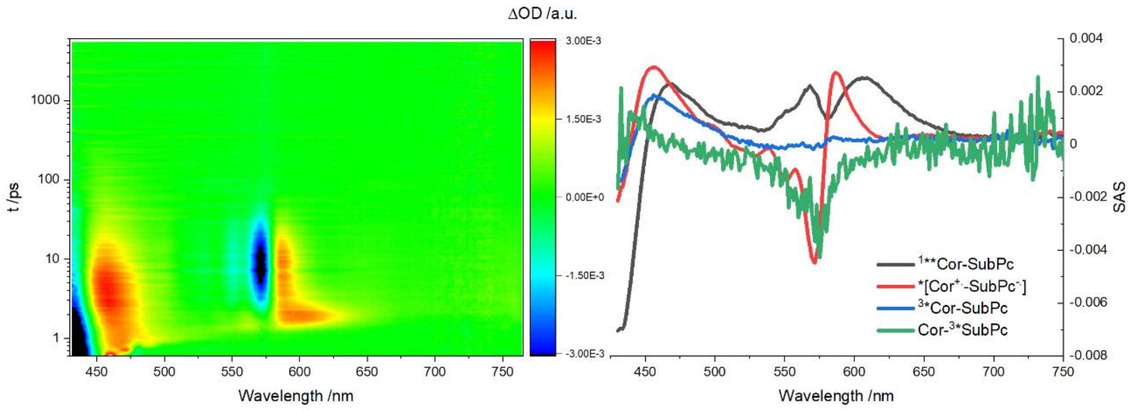

Figure S8.23. Differential absorption of **10** upon femtosecond flash photolysis with excitation at 420 nm in toluene (left) and corresponding species associated spectra of the involved transient species (right).

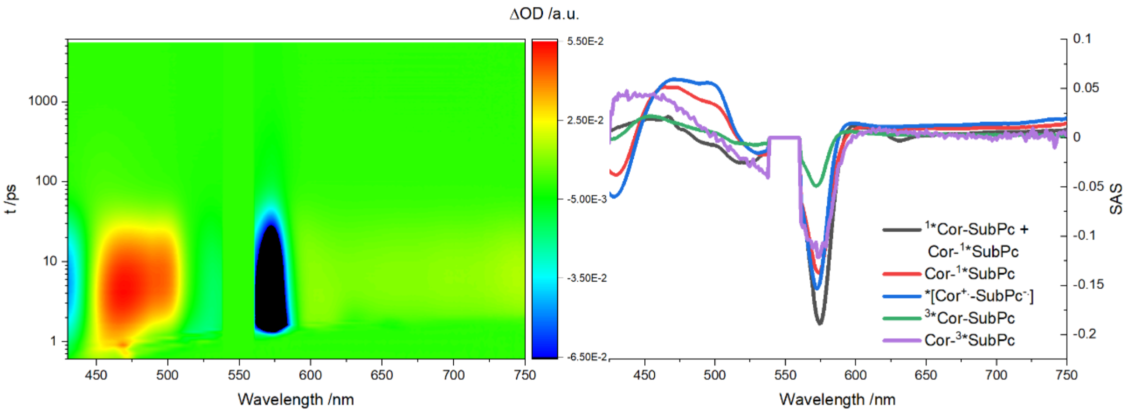

Figure S8.24. Differential absorption of **10** upon femtosecond flash photolysis with excitation at 550 nm in toluene (left)and corresponding species associated spectra of the involved transient species (right).

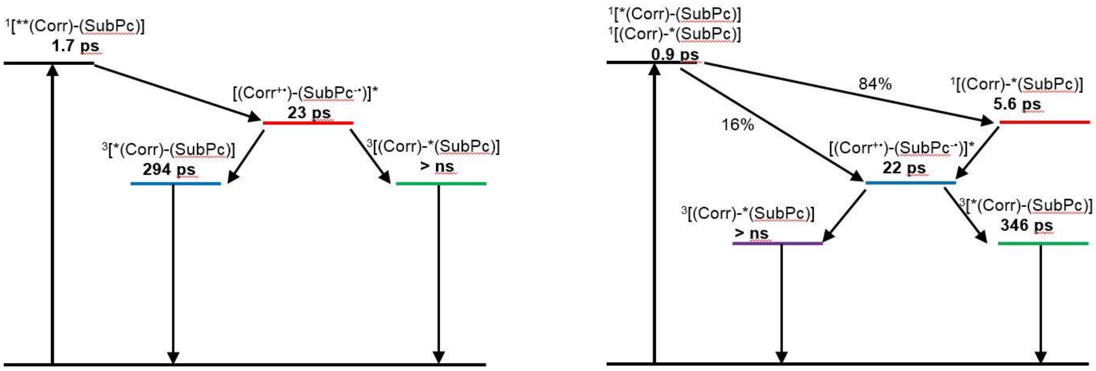

Figure S8.25. Kinetic models used for the analysis of femtosecond-resolved differential absorption of **10** upon excitation at 420 (left) and 550 nm (right) in toluene.

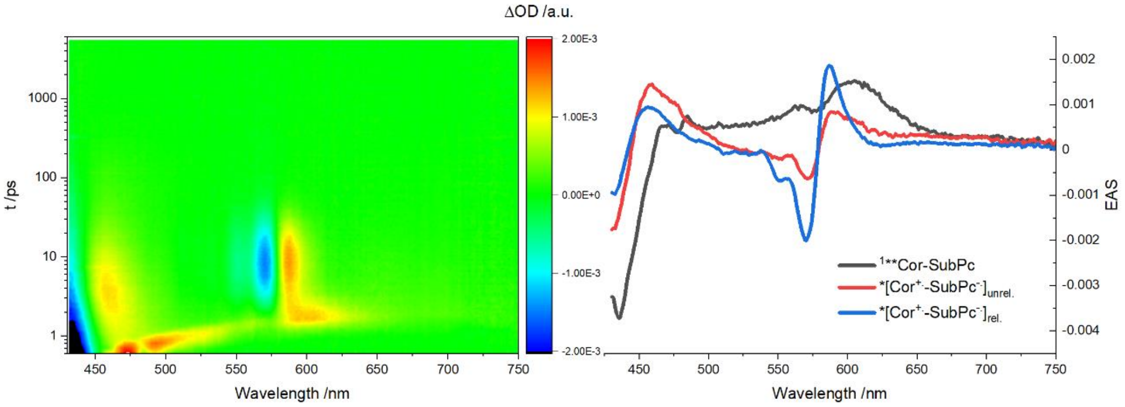

Figure S8.26. Differential absorption of **10** upon femtosecond flash photolysis with excitation at 420 nm in benzonitrile (left) and corresponding evolution associated spectra of the involved transient species (right).

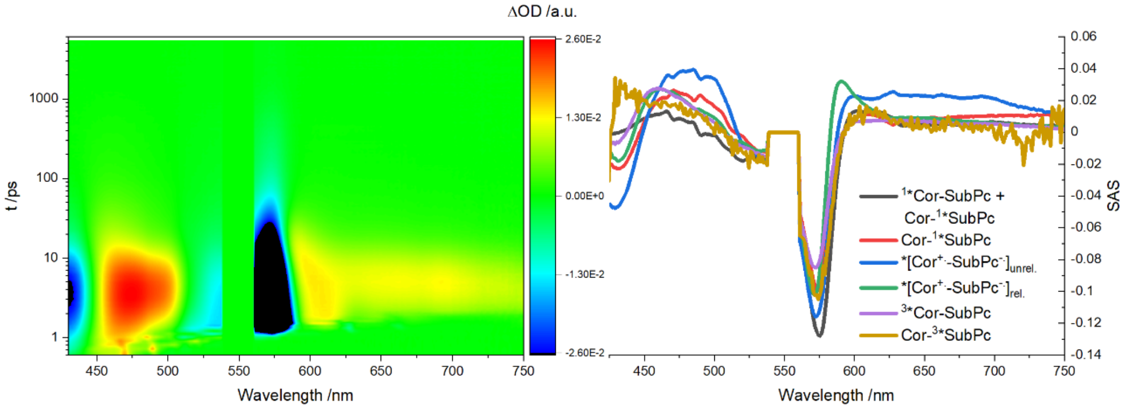

Figure S8.27. Differential absorption of **10** upon femtosecond flash photolysis with excitation at 550 nm in benzonitrile (left) and corresponding species associated spectra of the involved transient species (right).

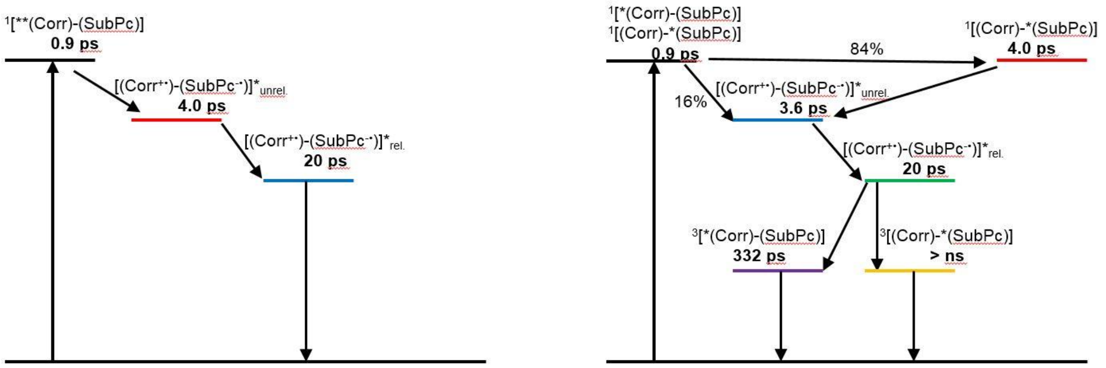

Figure S8.28. Kinetic models used for the analysis of femtosecond-resolved differential absorption of **10** upon excitation at 420 (left) and 550 nm (right) in benzonitrile.

SubPc-Cor **11**

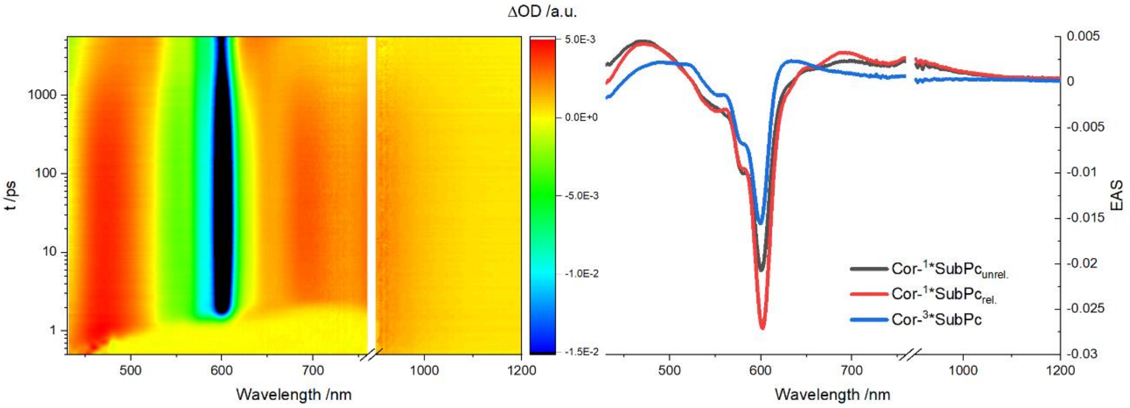

Figure S8.29. Differential absorption of **11** upon femtosecond flash photolysis with excitation at 420 nm in toluene (left) and corresponding evolution associated spectra of the involved transient species (right).

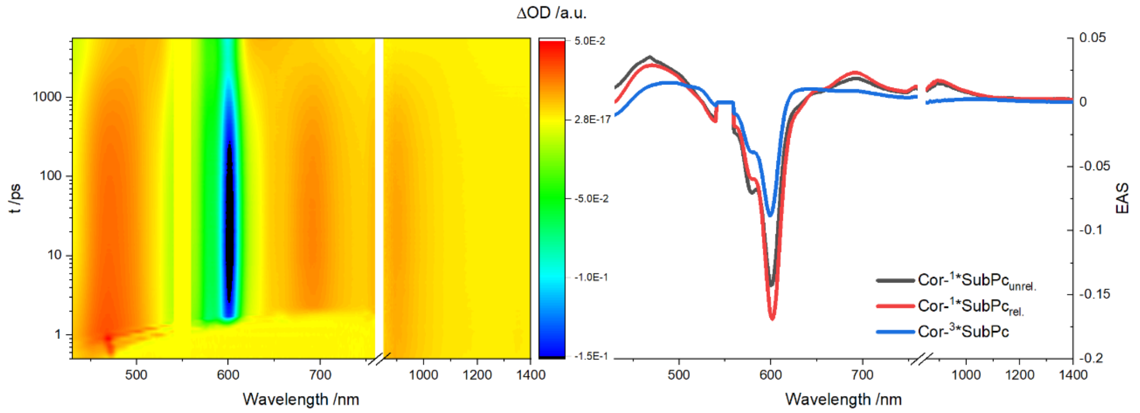

Figure S8.30. Differential absorption of **11** upon femtosecond flash photolysis with excitation at 550 nm in toluene (left) and corresponding evolution associated spectra of the involved transient species (right).

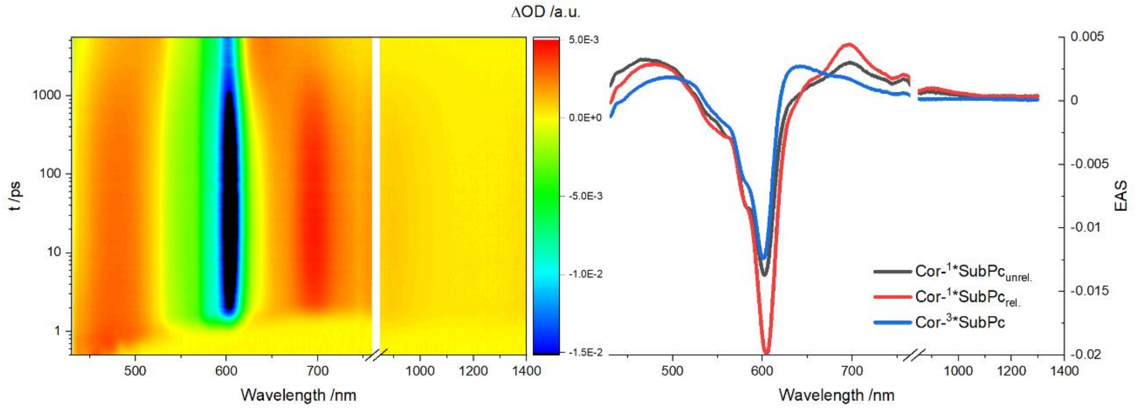

Figure S8.31. Differential absorption of **11** upon femtosecond flash photolysis with excitation at 420 nm in benzonitrile (left) and corresponding evolution associated spectra of the involved transient species (right).

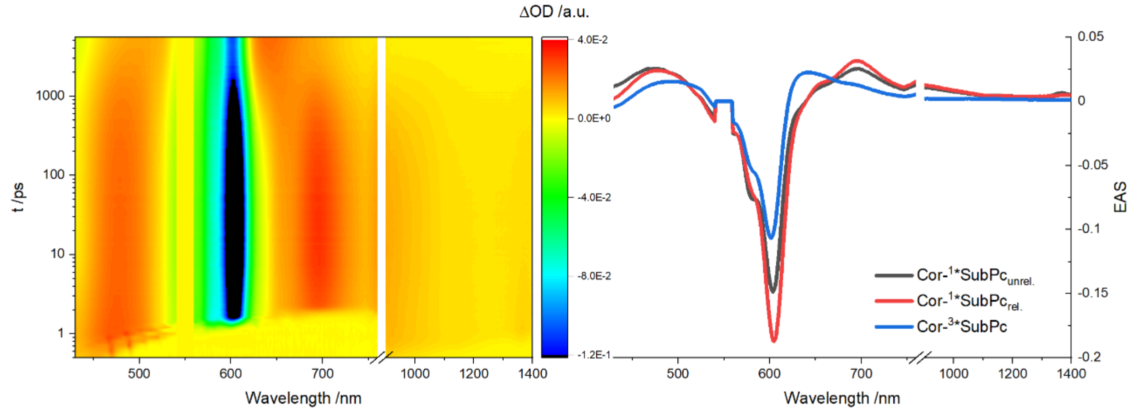

Figure S8.32. Differential absorption of **11** upon femtosecond flash photolysis with excitation at 550 nm in benzonitrile (left) and corresponding evolution associated spectra of the involved transient species (right).

[1] V. Král, P. Vašek, B. Dolenský, *Collect. Czech. Chem. Commun.* **2004**, *69*, 1126-1136.

[2] B. del Rey, U. Keller, T. Torres, G. Rojo, F. Agulló-López, S. Nonell, C. Marti, S. Brasselet, I. Ledoux, J. Zyss, *J. Am. Chem. Soc.* **1998**, *120*, 12808-12817.
[3] C. G. Claessens, D. González-Rodríguez, B. del Rey. T. Torres, G. Mark, H. Schuchmann, C. von Sonntag, J. G. MacDonald, R. S. Nohr, *Eur. J. Org. Chem.* **2003**, 2547-2551
[4] B. Koszarna, D. T. Gryko, *J. Org. Chem*. **2006**, *71*, 3707-3717.
[5] J. Vestfrid, R. Kothari, A. Kostenko, I. Goldberg, B. Tumanskii, Z.Gross, *Inorg. Chem.* **2016**, *55*, 6061-6067.
[6] R. Paolesse, T. Boschi, S. Licoccia, R. G. Khoury, K.M. Smith, *Chem. Commun*, **1998**, 1119–1120.
[7] N. R. Babij, E. O. McCusker, G. T. Whiteker, B. Canturk, N. Choy, L. C. Creemer, C. V. D. Amicis, N. M. Hewlett, P. L. Johnson, J. A. Knobelsdorf, F. Li, B. A. Lorsbach, B. M. Nugent, S. J. Ryan, M. R. Smith and Q. Yang, *Org. Process Res. Dev*., 2016, **20**, 661.
